# Unit-cell parameters determination from a set of independent electron diffraction zonal patterns

**DOI:** 10.1107/S2053273325000300

**Published:** 2025-01-31

**Authors:** Tatiana E. Gorelik, Gerhard Miehe, Robert Bücker, Kaname Yoshida

**Affiliations:** a Ernst Ruska-Center (ERC-1) Forschungszentrum Jülich Wilhelm-Johnen-Straße Jülich 52428 Germany; b Department of the Structure and Function of Proteins, Helmholtz Centre for Infection Research, Inhoffenstraße 7, Braunschweig, 38124, Germany; c Darmstadt Technical University, Schnittspahnstraße 9, Darmstadt, 64287, Germany; d Rigaku Corporation, 3-9-12 Matsubara-cho, Akishima-shi, Tokyo, 196-8666, Japan; e Rigaku Europe SE, Hugenottenallee 167, Neu-Isenburg, 63263, Germany; f Nanostructures Research Laboratory Japan Fine Ceramics Center 2-4-11 Mustuno, Atsuta-ku Nagoya 456-8587 Japan; Czech Academy of Sciences, Czechia

**Keywords:** electron crystallography, electron diffraction, 3D ED, microED, serial electron diffraction, unit-cell parameters determination

## Abstract

Due to the short de Broglie wavelength of electrons compared with X-rays, the curvature of their Ewald sphere is low, and individual electron diffraction patterns are nearly flat in reciprocal space. As a result, a reliable unit-cell determination from a set of randomly oriented electron diffraction patterns, an essential step in serial electron diffraction, becomes a non-trivial task. Here we describe an algorithm for unit-cell determination from a set of independent electron diffraction patterns, as implemented in the program *PIEP* (Program for Interpreting Electron diffraction Patterns), written in the early 1990s. We evaluate the performance of the algorithm by unit-cell determination of two known structures – copper perchlorophthalocyanine (CuPcCl_16_) and lysozyme, challenging the algorithm by high-index zone patterns and long crystallographic axes. Finally, we apply the procedure to a new, structurally uncharacterized five amino acid peptide.

## Abstract 2

We present an algorithm for unit-cell determination from a set of randomly oriented electron diffraction patterns and demonstrate its performance for two known structures (copper perchlorophthalocyanine and lysozyme) and one as-yet uncharacterized structure.

## 1. Introduction

All crystallographic analyses rely on the knowledge of unit-cell parameters. For single-crystal X-ray data, unit-cell determination usually requires one or more diffraction patterns and a geometrical description of the experimental conditions, such as radiation wavelength, camera length *etc*. The wavelength of X-rays is of the order of typical diffraction data resolution, such that the radius of the Ewald sphere is comparable with the size of the entire reciprocal lattice accessible in an experiment. As a result, each recorded pattern is a strongly curved slice through reciprocal space, giving rise to a 3D geometry of scattering vectors encoded in measured spot positions. Then, either using 3D difference vectors or Fourier transformation, the primitive lattice basis vectors can be extracted (Powell *et al.*, 2013 ▸).

The situation is different for electron diffraction (ED). While the exact de Broglie wavelength of electrons depends on the acceleration voltage, with typical values between 0.0335 Å (120 kV) and 0.0197 Å (300 kV) in transmission electron microscopes, those are two orders of magnitude smaller than wavelengths used in an X-ray experiment. As a result, the part of the Ewald sphere accessible via diffraction spots recorded in a single ED pattern at typical resolution is effectively flat, and essentially no 3D lattice information can be extracted. While reflections from higher-order Laue zones can be seen and used for the 3D lattice determination (Morniroli & Steeds, 1992 ▸; Shi, 2022 ▸), weakly scattering organic materials rarely show those reflections, making unit-cell determination from a single zonal pattern impossible.

Vainshtein (1964 ▸) proposed a simple 2D lattice reconstruction method based on a tilt series of ED patterns. These patterns were collected through a crystal tilt around a selected crystallographic axis, usually a low-index or main axis. For the lattice reconstruction it was essential to know the angular relationship between separated ED patterns. The method was effectively used for the unit-cell determination from ED patterns of many nanocrystalline materials (Wu & Spence, 2003 ▸; Kolb & Matveeva, 2003 ▸; Dorset *et al.*, 2005 ▸; Gorelik *et al.*, 2009 ▸; Sun *et al.*, 2010 ▸). One of the highlights of the method was the discovery of quasicrystals in the 1980s (Shechtman *et al.*, 1984 ▸).

Following this approach, a so-called Vainshtein plot could be constructed in 3D, mapping out a sufficient section of the 3D reciprocal space for lattice parameter determination. The procedure included two steps: (i) the reduction of experimental ED patterns into sets of reflection coordinates, and (ii) the reconstruction of 3D coordinates of these reflections based on the known angular relationship between the patterns. This was a relatively simple geometrical task; several home-written packages were used for this purpose, and even a commercial software (*TRICE*) was created (Zou *et al.*, 2004 ▸).

The idea of using a defined angular relationship between patterns was initially the basis for the development of 3D ED (also known as microED) techniques pioneered by the Mainz group (Kolb *et al.*, 2007 ▸; Kolb *et al.*, 2008 ▸). Later, the idea of zone pattern collection was abandoned in favour of a method involving the tilting of crystals around an *arbitrary* crystallographic axis in small, regular goniometer tilt steps or during continuous tilting (continuous rotation) (Nederlof *et al.*, 2013 ▸; Nannenga *et al.*, 2014 ▸), resulting in the collection of off-axis patterns, in analogy to a typical single-crystal X-ray diffraction experiment. By projecting the reflection positions onto 3D reciprocal space and analysing their coordinates, the 3D lattice vectors can be extracted. The 3D ED/microED method has fast gained popularity and is now an established technique of structure analysis, applied to diverse materials (Gemmi *et al.*, 2019 ▸) using both electron microscopes and dedicated diffractometers (Ito *et al.*, 2021 ▸; Simoncic *et al.*, 2023 ▸).

Application of 3D ED to extremely electron beam sensitive materials is still a challenging task. For relatively large crystals, so-called ‘helical’ data collection can be used – with a small electron beam moving along the crystal as it is being tilted (Brázda *et al.*, 2019 ▸). In this way, all patterns are collected from a fresh, previously unexposed area of the sample, while retaining a known relative orientation. Still, in certain cases, the beam sensitivity of a crystal does not allow collection of even a short tilt sequence, which would allow the lattice basis vectors to be obtained. Furthermore, large, thin crystals are often bent, or deform under irradiation. As a result, the 3D diffraction volume is highly distorted and difficult to analyse.

The problem of small, beam-sensitive crystals is well known in protein X-ray crystallography and led to the development of the serial crystallography approach (Chapman *et al.*, 2011 ▸), where single diffraction snapshots of a large number of randomly oriented crystals are collected, using X-ray free-electron lasers (XFELs) or microfocus synchrotron beamlines (Stellato *et al.*, 2014 ▸). For XFELs, the irradiation time on the femtosecond scale is deemed shorter than relevant damage processes, whereas at synchrotrons radiation doses per snapshots are matched to the damage threshold of the crystal. A single diffraction pattern is collected from an individual crystal, effectively distributing radiation damage over many crystals. Indexing of each diffraction pattern and subsequent merging of data from all crystals creates high-quality datasets, able to provide a reliable structure solution. Mature packages for snapshot processing are now available (White *et al.*, 2012 ▸, 2016 ▸; Brewster *et al.*, 2018 ▸; Kabsch, 2014 ▸).

The attractive idea of automatic single diffraction pattern collection from individual nanocrystals was readily taken up by electron crystallographers (Smeets *et al.*, 2018 ▸; Bücker *et al.*, 2020 ▸; Hogan-Lamarre *et al.*, 2024 ▸). The application of serial electron diffraction was successfully demonstrated for different zeolites (Smeets *et al.*, 2018 ▸), hen egg-white lysozyme and crystalline granulovirus shells (Bücker *et al.*, 2020 ▸). Reflection indexing in separate ED patterns was done based on the prior knowledge of the lattice parameters.

The need for prior knowledge of the unit-cell metric restricts enormously the use of serial electron diffraction. As mentioned above, ED patterns are essentially ‘flat’ and do not contain 3D information. However, a suitable mathematical treatment of a combined set of ED patterns should provide information on all three lattice vectors.

An algorithm for unit-cell determination from randomly oriented ED patterns was proposed by Jiang *et al.* (2009 ▸). In the first step, similarly to Vainshtein’s method, information in diffraction patterns was reduced to the coordinates of Bragg reflections. In each pattern two shortest pattern basis vectors were defined. Autocorrelation of the whole diffraction pattern was used to assist the vector determination. From these pairs of vectors, triangular facets were constructed, characterized by the lengths of the two vectors and the angle between them. Assuming the Ewald sphere is essentially flat, the following holds: intersection of a 3D reciprocal lattice with a diffraction plane generates a 2D lattice (zone pattern), thereby defining a facet. In the second step, a list of potential 3D unit cells was generated by a grid search, and principal facets were calculated for each cell. The match of experimental and simulated facets was characterized by a figure of merit (FOM). The lowest FOM provided the correct unit cell. This algorithm was implemented in the software package *EDIFF* (Jiang *et al.*, 2011 ▸) and was validated by unit-cell determination of mayenite, potassium penicillin G, sodium oxacillin and the orthogonal morphology nanocrystals of hen egg-white lysozyme (Jiang *et al.*, 2009 ▸).

A few decades earlier, a similar program was developed by Miehe (1997 ▸), which contains a set of simple and powerful algorithms that hitherto were unpublished. The program was named *PIEP* (Program for Interpreting Electron diffraction Patterns) and included various options for ED data processing, such as navigation in reciprocal space, determination of unit-cell parameters for indexing of X-ray powder diffraction data, searching for a crystalline phase in subsets of the Inorganic Crystal Structure Database (ICSD) (https://icsd.products.fiz-karlsruhe.de/), and more. Among other functions, determination of the unit-cell basis vectors from a set of randomly oriented ED patterns was implemented, successfully used in many structural studies (Horvath-Bordon *et al.*, 2007 ▸; Schmitt *et al.*, 2010 ▸; Liu *et al.*, 2016 ▸).

**Figure 1:**
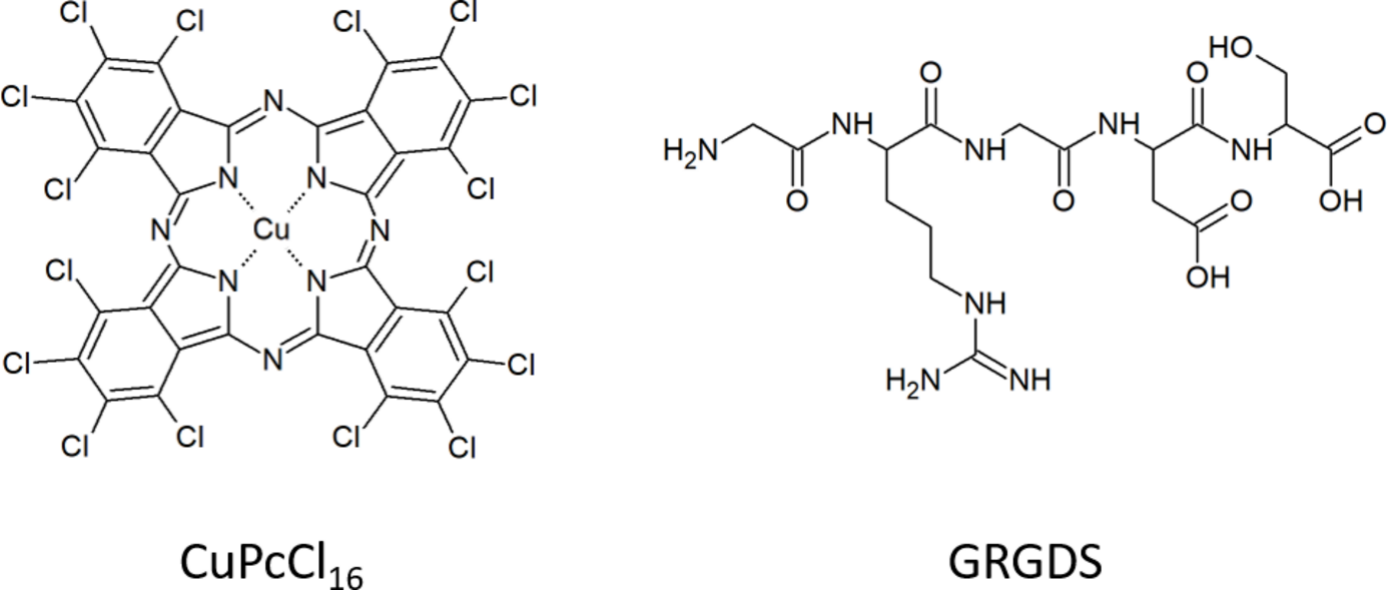
Molecular schemes of the studied compounds: chlorinated copper phthalocyanine and the peptide GRGDS.

With the recent developments in the field of serial ED (Bücker *et al.*, 2020 ▸), we foresee the need for a robust algorithm for unit-cell determination from a set of diffraction patterns with uncorrelated orientations. Therefore, we decided to test the GM (Gerhard Miehe) algorithm as implemented in *PIEP* for cell metric determination for several materials. Three different materials were used for the study, two with known structures (chlorinated copper phthalocyanine and lysozyme), and a crystalline peptide GRGDS, which has not yet been structurally characterized. The molecular structures of the chlorinated copper phthalocyanine and GRGDS peptide are shown in Fig. 1 ▸. In this work we describe the algorithm for unit-cell parameter determination used in *PIEP*, discuss its strengths and limitations, and demonstrate the use of *PIEP* for unit-cell determination from a set of experimental randomly oriented ED zonal patterns for three different compounds.

## 2. Methods

### 2.1. Materials

#### 2.1.1. Copper perchlorophthalocyanine

The copper perchlorophthalocyanine (CuPcCl_16_) nanocrystals were prepared directly on a TEM (transmission electron microscopy) grid as described by Gorelik *et al.* (2021 ▸). Bright-field scanning transmission electron microscopy (STEM) images were taken with a ThermoFisher Scientific TALOS transmission electron microscope.

**Figure 2:**
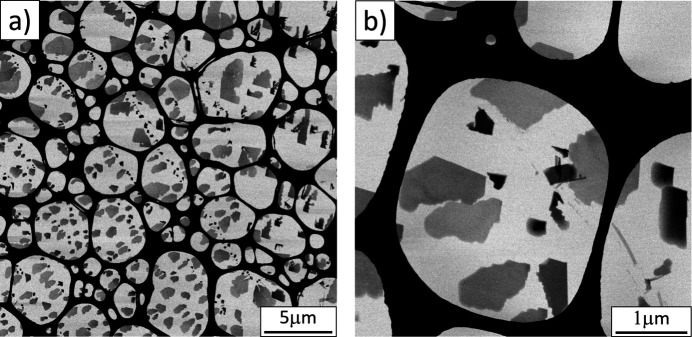
Bright-field STEM images of CuPcCl_16_ crystals recorded at different magnification.

The obtained crystals were platelets with a very distinct morphology (Fig. 2 ▸) with an approximate lateral size of 0.5 µm. Occasionally, needle-like crystals were found. Despite the difference in morphology, the needle-like crystals had the same crystalline structure as the platelets. Platelets had a wedge-like shape; their thickness varied from a few nm to 30 nm (Yoshida *et al.*, 2015 ▸).

#### 2.1.2. Lysozyme data

**Figure 3:**
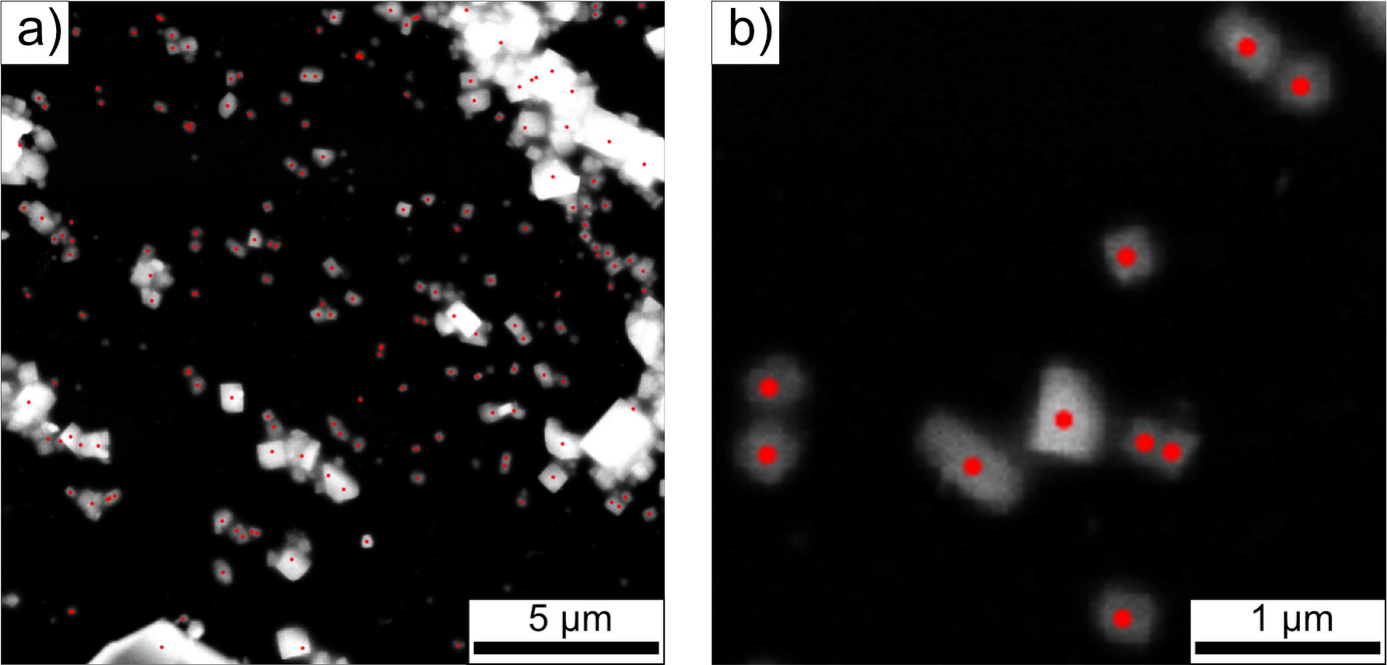
Typical dark-field STEM image of vitrified lysozyme crystals. (*a*) Grid region from which serial ED data were collected within a single run, by sequentially moving the beam on the crystals after automatic selection using image processing (red dots). (*b*) Close-up of a sub-region with single sub-micrometre lysozyme crystals.

Lysozyme is a single-chain polypeptide of 129 amino acids with a molecular weight of 14307 Da (Jollès, 1969 ▸). Different polymorphs of lysozyme have been reported in the Protein Data Bank (PDB) (Bernstein *et al.*, 1977 ▸); here ED data of a tetragonal form, crystallizing in the space group *P*4_3_2_1_2, were used (Weiss *et al.*, 2000 ▸). The data analysis carried out in this work uses the serial electron crystallography data as presented by Bücker *et al.* (2020 ▸). In brief, lysozyme crystals were crushed by vortexing to obtain sub-micrometre-sized crystallites which were plunge-frozen on a TEM grid in liquid ethane. In Fig. 3 ▸, a typical dark-field STEM image of a grid region is shown. After automatic identification of crystals in the images, ≃1300 diffraction patterns were collected from a few dozen regions.

#### 2.1.3. GRGDS

**Figure 4:**
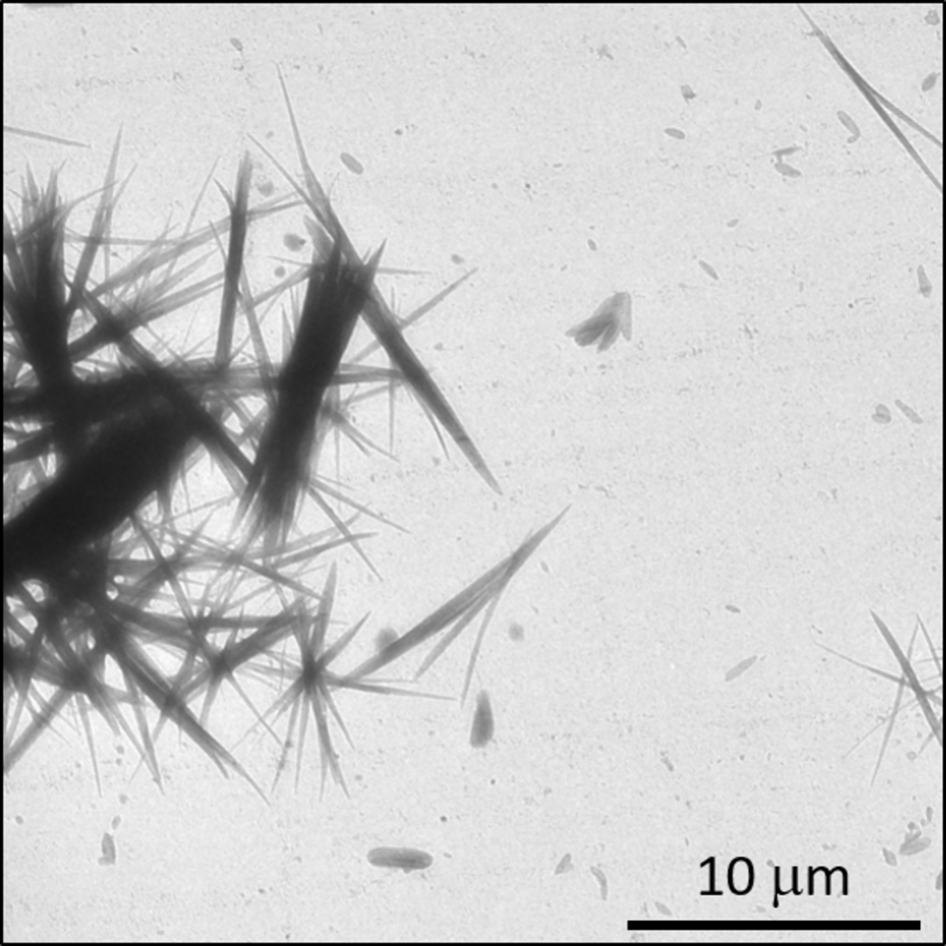
TEM image of GRGDS crystals.

GRGDS is a five amino acid peptide. The crystal structure of the material is unknown. GRGDS peptide was purchased from GenScript Biotech (Leiden, Netherlands). After several attempts to obtain nanocrystals suitable for ED structure analysis, crystals of GRGDS were eventually grown from methanol directly on TEM grids using the following procedure: a drop of the solution was placed onto a carbon-coated copper TEM grid and slowly dried in a vessel with a volume less than 1 cm^3^, closed by a piece of preparative glass. These crystals were then used for the unit-cell determination. GRGDS crystals grew as agglomerates of needles with a width of less than 0.5 µm and a length of around 10 µm (Fig. 4 ▸).

The molecular volume estimated from the molecular structure (Hofmann, 2002 ▸) is 585 Å^3^.

We recently determined the crystal structure of GRGDS from 3D ED data. The structure was revealed to be a co-crystal of GRGDS with tri­fluoro­acetic acid (TFA), crystallizing in the *C*2 space group with lattice parameters *a* = 29.231, *b* = 4.546, *c* = 19.640 Å and β = 106.70°. The original diffraction data for the GRGDS–TFA co-crystal can be accessed at DOI: 10.5281/zenodo.13938422, and the CSD (Cambridge Structural Database) deposition code for the structure is 2391399. Further details regarding the structure determination will be published elsewhere.

### 2.2. Electron diffraction data collection

For CuPcCl_16_ and GRGDS samples the TEM experiments were performed using a ThermoFisher Titan transmission electron microscope operated at 300 kV and equipped with an objective Cs corrector. The data were collected using a Fischione Advanced Tomo Holder 2020 at room temperature. In TEM mode the beam-forming optics were set to nanodiffraction mode with the C2 aperture of 50 µm. Diffraction patterns were recorded with an effective beam diameter on the sample that varied between 100 and 500 nm. The samples were randomly tilted in order to access mostly different zone axes. Diffraction data were recorded with a 2k Gatan UltraScan CCD.

For lysozyme, data were collected on a Philips Tecnai F20 scanning/transmission electron microscope equipped with an X-Spectrum Lambda 750k pixel array detector in defocused nanoprobe STEM mode with a C2 aperture of 5 µm, resulting in a collimated beam of approximately 110 nm. Crystals previously found in dark-field STEM images of grid regions were addressed by the beam using direct control of the STEM deflectors, synchronized to detector read-out. The data were pre-processed using the package *diffractem* (Bücker *et al.*, 2021 ▸) assuming lattice constants of *a* = *b* = 79.1, *c* = 38 Å, which led to successful indexing of ≃1050 patterns using the *pinkIndexer* grid-search algorithm (Gevorkov *et al.*, 2020 ▸). The raw diffraction data and data processing results are available from the Max Planck Digital Library EDMOND data repository at https://doi.org/10.17617/3.53; the resultant protein structure is available from the wwPDB using the code 6S2N.

### 2.3. Characteristic electron dose

For CuPcCl_16_ and GRGDS, series of ED patterns were sequentially collected from the same part of the crystals to quantify the electron radiation stability of the sample. The data were measured at room temperature. The characteristic electron dose is defined as the point at which the intensities of ED peaks are reduced to 1/e of the initial value (Kolb *et al.*, 2010 ▸). Reflections within different resolution shells showed slightly different decay profiles. An average value was used for the characteristic dose calculation. The characteristic electron dose at 300 kV and room temperature for CuPcCl_16_ was measured to be 7.6 × 10^3^ e Å^−2^, for GRGDS 0.5 e Å^−2^. For lysozyme, as discussed by Bücker *et al.* (2020 ▸), dose-fractionated diffraction exposures were used; optimum data quality was obtained for a dose of 2.6 e Å^−2^ per crystal.

### 2.4. Zone basis vectors extraction

From a pool of ED patterns, some particularly prominent ones, that is, with the shortest interplanar distances and with the highest symmetry, were selected by visual inspection. For these patterns, the basis vectors were calculated using two different approaches: based on manual selection of lattice basis vectors with subsequent least-squares refinement (Section S1.1, supporting information), and autocorrelation of diffraction patterns (Section S1.2, supporting information). For each pattern, the most confident solution was used.

### 2.5. The GM algorithm

In the following, the algorithm (here referred to as the GM algorithm, for the developer Gerhard Miehe) that underlies the determination of unit-cell parameters from a set of independent ED zonal patterns by *PIEP* will be presented. Like the algorithm of Jiang *et al.* (2009 ▸, 2011 ▸), the GM algorithm uses a trial-and-error approach. The applied strategy, however, is different. It has briefly been described by Miehe (1997 ▸) and will be detailed here.

The initial step in data processing involves the reduction of a 2D experimental ED pattern to a set of two basis vectors. The vectors can either be defined by the scalar lengths of two vectors, |**r**_1_| and |**r**_2_|, and an angle between them, φ, or as the scalar lengths of three vectors (|**r**_1_|, |**r**_2_|, vector |**r**_1_ − **r**_2_|, optionally |**r**_1_ + **r**_2_|).

**Figure 5:**
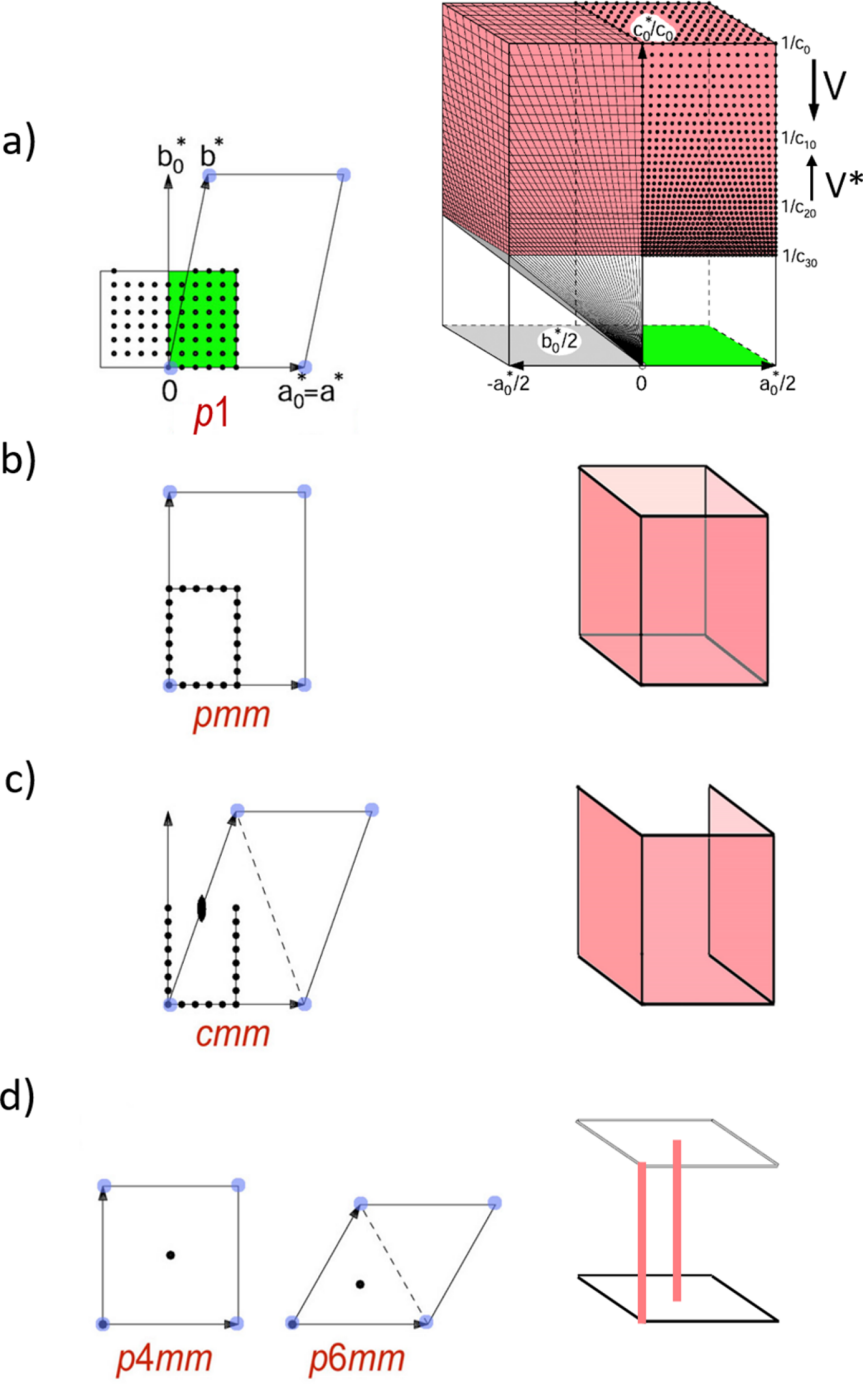
Schematic representation of the scan dimensionality for different symmetries: (*a*) a general case, initial pattern has symmetry *p*1, 3D cell search. Left side: the (*hk*0) plane showing the **a**_0_ and **b**_0_ vector definition and the base of the unit cell for the cell search; right side: a scheme for the grid search in reciprocal space. The *z* axis represents V*. Scans are performed in layers of constant V*, varying the x* and y* components of the vector **c**. (*b*) The initial pattern has symmetry *pmm*, reducing the search to a 2D search along four mirror planes; (*c*) initial pattern with symmetry *cmm* – 2D search along three mirror planes; (*d*) initial pattern corresponding to a mirror plane (*e.g.* monoclinic crystal system, initial pattern [010] zone) and higher symmetries – 1D scan.

Direct and reciprocal-lattice vectors are related by well known equations. Six cell parameters (lengths *a*, *b*, *c* and angles α, β, γ, or their reciprocal counterparts’ lengths a*, b*, c* and angles α*, β*, γ*) define a primitive unit cell. The key premise of the GM algorithm is the fact that *each of the N given ED patterns is suited to define zone [001] of one setting of the associated unit cell*. Hence, three of the six reciprocal cell parameters of that cell can be considered as known – say, a*, b* and γ*. The missing three cell parameters are found by scanning vector c*, defined by its three components x*, y*, z* on an appropriate grid, using an appropriate step width within a 3D vector space spanned by the orthogonal system:
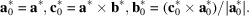
The grid scan is parametrized using scalar coordinates x*, y*, V*, where x* and y* are defined as components of c* along the *a*_0_* and *b*_0_* axes, respectively. To inherently optimize the domain and step size of the grid search for the problem at hand, the third scan coordinate is given by the reciprocal-lattice volume V*, implicitly defining the component of c* along the *c*_0_* axis. The 3D scan is performed in layers of constant V*:
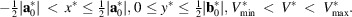


This range [Fig. 5 ▸(*a*)] delineates the primitive unit cell for the most general case, which will have symmetry *p*1. For the symmetry notation, we use the plane group of the pattern, disregarding the intensity of reflections.

Within this scan range, candidate cells are generated from each unique value of c* and used to attempt indexing of the remaining *N* − 1 patterns. If indexing fails for any of those, the current value of c* is discarded, and the procedure is repeated using the next candidate. If all patterns can be indexed within given tolerances, the cell is stored together with a reliability index *R*, which is derived from the individual indexing tolerances of the *N* − 1 patterns. These individual indexing tolerances, in turn, are formed from the sum of the weighted mismatches of (i) the ratio of basis vectors in the diffraction pattern |**r**_1_|/|**r**_2_|, (ii) the angle between the vectors in the pattern φ, and (iii) the overall pattern scaling factor (camera constant *C*, see the supporting information). The weighting parameters, *w*_1_, *w*_2_ and *w*_3_, are defined in the ASCII parameters file piep.par and can be modified as needed. The specific set of values: *w*_1_ = 0.008 (equivalent to 0.8% contribution from the mean |**r**_1_|/|**r**_2_| mismatch), *w*_2_ = 0.006 (0.6% contribution from the φ mismatch) and *w*_3_ = 0.003 (0.3% contribution from the pattern scaling mismatch) was empirically determined to produce stable runs and was used in all calculations,
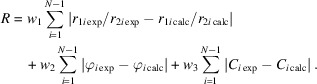


If possible, higher Laue zones should be evaluated to determine rough limits for the expected volume of the reduced cell and to recognize possible mirror planes (see below) in the structure (Shi & Li, 2021 ▸).

*PIEP* uses a non-equidistant set of *V* values, with a constant fractional increment *f*, so that 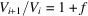. The default value of *f* is 0.025.

After the last layer (V* = V*_max_) has been processed, the reduced settings of the stored cells are displayed, sorted by the *R* indices. The correct cell should be found at the very beginning of the list. Optionally, a Delaunay reduction (Patterson & Love, 1957 ▸) is performed to determine the conventional settings of cells. For this purpose, the slightly modified code of the program *DELOS* (Zimmerman, 1985 ▸) is integrated in *PIEP*.

For this most general strategy, the handling of triclinic symmetry is intrinsic. The accuracy of results depends mainly on the accuracy of input data. In test runs with a set of simulated diffraction patterns, assuming perfect measurement, the accuracy of the found unit cell depends solely on the step size of the scan, which can be minimized at will.

If a diffraction pattern displays a ‘true’ mirror plane (see below), either *pmm* or *cmm*, the symmetry of the associated cell will be higher than triclinic. Such patterns are particularly suited for defining the initial zone [001].

A mirror plane within the plane *hk*0 is ‘*true*’ (not accidental) if it acts also on plane *hk*1, the trace of which is the first-order Laue zone (FOLZ). If FOLZ reflections are visible, this condition can be verified. If extinctions due to a screw axis are present, it is surely fulfilled. Any reflection in plane *hk*1 may serve as a reflection 001. Therefore, the search can be confined to the four potential mirror planes: *x* = 0, *x* = ½|**a**_0_*|, *y* = 0 and *y* = ½|**b**_0_*| (for the *pmm* case). The asymmetric units within these mirror planes are given below. This way, the scan becomes 2D [Fig. 5 ▸(*b*)],
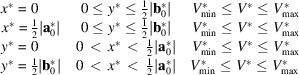
(for *pmm* only).

This condition can be easily explained for a monoclinic structure. If a zone pattern displays symmetry 2*mm*, the unique monoclinic axis must be one of the orthogonal basis vectors of this zone – either **a**_0_* or **b**_0_*. The other basis vector will correspond to one of the *h*0*l* reflections. The third, unknown basis vector of the lattice, c*, must be orthogonal to the unique monoclinic axis and thus must lie in a vertical plane, either orthogonal to **a**_0_* or **b**_0_*. Consequently, the possible c* vectors span these vertical planes [Fig. 5 ▸(*b*)], with the a*–b*-defining plane being any of [*h*0*l*]. For a monoclinic-centred lattice, the situation is somewhat different. The *cmm* pattern can have any [*h*0*l*] index with *l* ≠ 0. This condition further restricts the number of solutions [Fig. 5 ▸(*c*)].

The distribution of solutions can be visualized by plotting an ‘*inverse FOM*’ *F*_inv_ = 1/*R* (maximum for best fit) in these four planes: *F*_inv_ = *F*_inv_ (y*, V*) and *F*_inv_ = *F*_inv_ (x*, V*). For special cases of *pmm* and *cmm*, namely *p*4 and *p*6, respectively, the range to be scanned can further be restricted; two linear scans of V* will be sufficient [Fig. 5 ▸(*d*)]. If the initial zone [001] displays symmetry *p*4*m*, the unit cell ought to be tetragonal. V* is scanned between V*_min_ and V*_max_, the reliability indexes becoming lines *R*(0,0,V*) and *R*(½,½,V*). For the symmetry *p*6*m* the corresponding 1D scans are *R*(0,0,V*) and *R*(⅓,⅓,V*), defined within the basis system **a**_0_*, **b**_0_*, **c**_0_*.

Larger unit cells are more likely to accommodate all experimental patterns, leading to a greater number of potential solutions with high unit-cell volumes. These solutions may also produce higher peaks on the 1/*R* surface. However, the correct solution should exhibit a sharp peak and correspond to a reasonably low volume on the 1/*R* surface.

The number of solutions depends strongly on (i) the error limits defined for the input data, (ii) the number of patterns and (iii) the prominence of zones recorded (how low the zone indices are). Typical numbers of patterns used for the unit-cell determination are: 4–6 for a 3D scan, 3–5 for 2D and 2–3 for 1D search. During a run, these numbers may dynamically be increased, and suspicious patterns may be excluded.

## 3. Results

### 3.1. CuPcCl_16_

Copper phthalocyanine is a highly polymorphic compound (Herbst & Hunger, 2004 ▸), yet only one crystalline phase has been observed for its chlorine derivative – copper perchloro-phthalocyanine CuPcCl_16_ (Gorelik *et al.*, 2021 ▸). The unit cell is *C*-centred monoclinic (*C*2/*m*, *Z* = 8) with the lattice parameters *a* = 17.685 (4), *b* = 25.918 (5), *c* = 3.8330 (8) Å, β = 95.05 (3)°, and the unit-cell volume is 1750.1 Å^3^. The unit cell contains two molecules, the asymmetric unit includes ¼ of the molecule, and thus the volume of a single molecule is 875.05 Å^3^ (a half of the unit-cell volume).

The primitive reduced cell corresponding to the structure has a metric of *a* = 3.833, *b* = 15.688, *c* = 15.688 Å, α = 111.39°, β = 92.84°, γ = 92.84°, and can be transferred back to the centred monoclinic cell using the transformation matrix [011; 011; 100].

**Figure 6:**
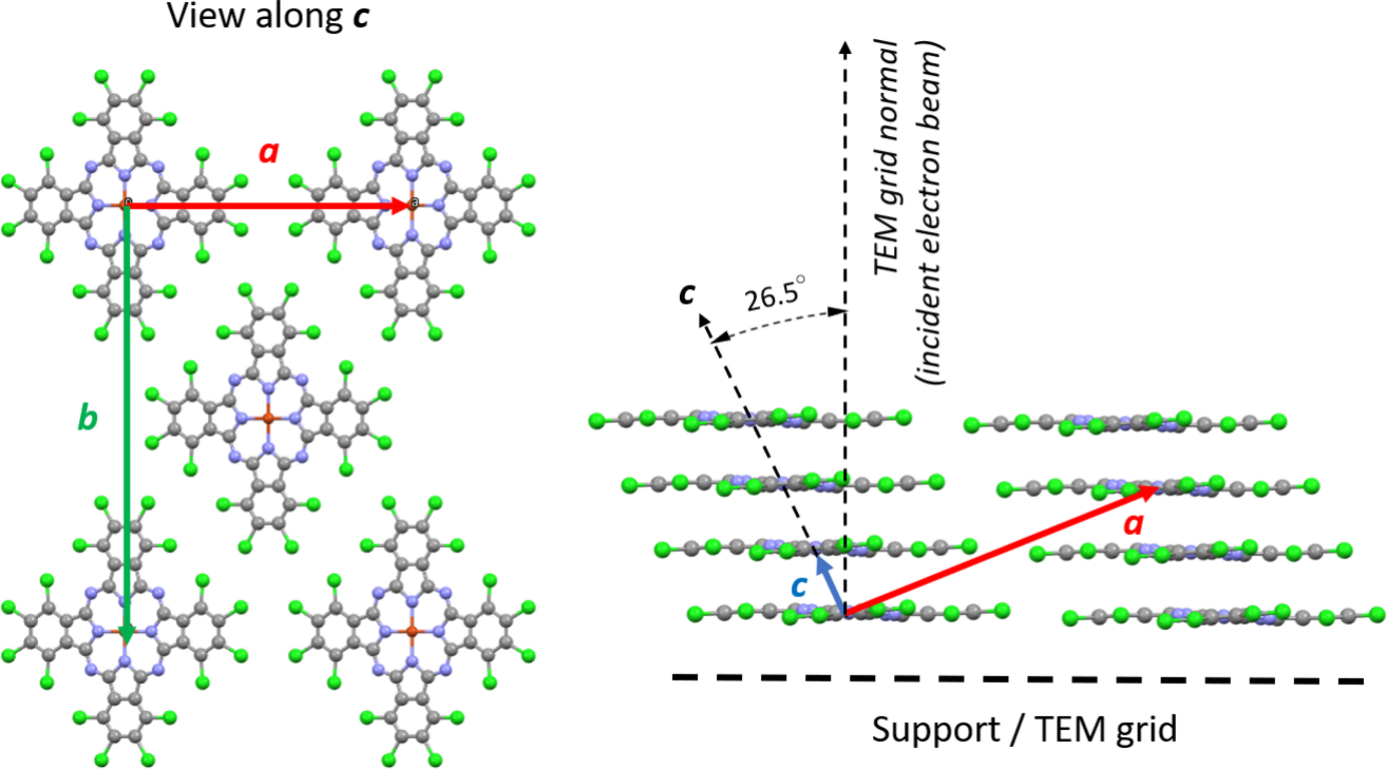
Crystalline structure of CuPcCl_16_ viewed along **c** (left) and **b** (right). In the **b** projection some molecules within the unit cell are omitted for clarity.

The crystal structure of CuPcCl_16_ contains layers of flat molecules, stacked along an inclined axis (Fig. 6 ▸). When viewed along the *c* axis, the shape of the molecule is seen to be slightly contracted along **a** due to the projection. In the sample preparation procedure used, the molecules are aligned flat on the supporting film. The angle between the molecular normal vector and the crystallographic *c* axis can be calculated from the crystal structure and is 26.5°. The main crystallographic axis [001] hence lies only 26.5° away from normal incidence on the TEM grid and can easily be reached by sample tilting. Note that this is a rather unusual scenario for electron crystallographic investigation. More commonly, the crystal direction associated with the longest crystallographic axis is the least developed, meaning the least amount of crystal growth occurs in this direction. As a result, this axis often aligns with the beam incidence, and due to the limited tilt range of TEM grids, it is rarely observed experimentally [unless specialized sample preparation protocols are used (*e.g.* Wennmacher *et al.*, 2019 ▸)]. In the case of CuPcCl_16_, the crystals adopt this rather unusual morphology and orientation due to the epitaxy on a KCl crystal.

**Figure 7:**
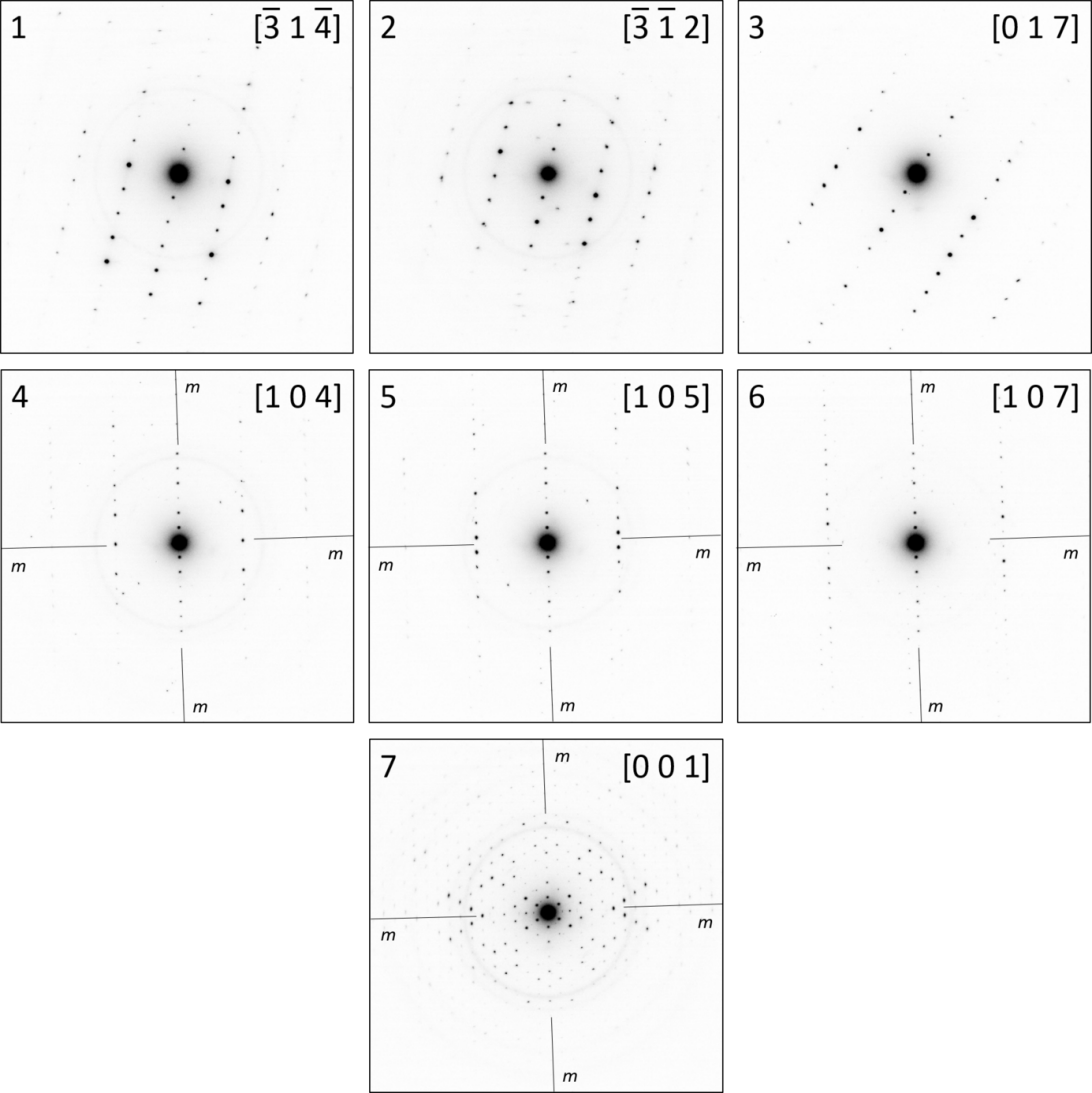
Seven zonal patterns of CuPcCl_16_ used for lattice parameters determination. The zone pattern 7 displays *cmm* symmetry with two mirror planes – vertical and horizontal. Vertical mirror planes are indicated in patterns 4, 5, 6 and 7.

Seven zone patterns (Fig. 7 ▸) were evaluated for unit-cell determination (Table 1 ▸). In each of these zonal patterns, the lengths of two basis vectors and the angles between them were extracted and used as input for *PIEP*. A step-by-step guide with detailed explanations of the procedure is presented in Section S3.2 in the supporting information.

Pattern 7 contains the two longest vectors and was therefore chosen by the program to represent the initial [001] zone. No reflection intensity values are provided to the program; the symmetry is automatically estimated solely from the metric of the provided basis vectors and the angle between them. As *d*_1_ and *d*_2_ have slightly different lengths, symmetry *cmm* was not assigned to this pattern, so a full 3D search was initiated. For the search range and resolution, *V*_max_ = 1000 Å^3^ and a fractional increment *f* of 0.025 have been specified, and the program reports that 786 cells will be generated in 12 volume layers between *V*_min_ = 763 Å^3^ and *V*_max_ = 1000 Å^3^. *V*_min_ is calculated as the smallest unit cell possible for a given [001] zone. Essentially, it is the area of the base of the unit cell for the cell search [Fig. 5 ▸(*c*)]. The combination of the *V*_max_ = 1000 Å^3^ and a fractional increment of 0.025 gives 12 values for unit-cell volume: (763.0, 782.1, 801.6, 821.7, 842.2, 863.3, 884. 8, 907.0, 929.6, 952.9, 976.7, 1001.1).

The unit-cell search took less than 1 s (Intel processor 2.1 GHz dual core), 23 solutions were returned (reduced setting of unit cells), sorted by figure of merit *R*. The five solutions with the best *R* factors are shown in Table 2 ▸.

The solution with the lowest figure of merit *R* (0.85) has a cell volume of 828.5 Å^3^, which is close to the volume of a single molecule of 875.05 Å^3^ (see above). These values match well the metric of the known reduced primitive cell of *a* = 3.833, *b* = 15.688, *c* = 15.688 Å, α = 111.39°, β = 92.84°, γ = 92.84°. Delaunay reduction implemented in *PIEP* suggested an *A*-centred monoclinic cell with the parameters *a* = 3.817, *b* = 25.567, *c* = 17.329 Å, α = 88.70°, β = 95.35°, γ = 90.20°. A transformation with the matrix [001; 010; 100] yields a *C*-centred monoclinic cell with the parameters *a* = 17.329, *b* = 25.567, *c* = 3.817 Å, α = 89.80°, β = 95.35°, γ = 91.30°, *V* = 1683.5 Å^3^, matching well the expected values.

We then imposed symmetry *cmm* on pattern 7 by setting the lengths of the two vectors equal to their average value (producing pattern 8; see the supporting information). This situation corresponds to the case presented in Fig. 5 ▸(*c*), where a 2D search within three planes is necessary to determine the unit-cell metric. Significantly fewer search sets (197) were generated, resulting in three solutions. The best solution, *a* = 3.82, *b* = 15.44, *c* = 15.44 Å, α = 111.8°, β = 92.7°, γ = 92.7°, yielded a *C*-centred monoclinic cell with a metric of *a* = 17.3087, *b* = 25.5637, *c* = 3.8176 Å, α = 90.000°, β = 94.74°, γ = 90.00°, which is nearly identical to the best solution obtained from a 3D search.

The obtained unit-cell parameters were used to index all diffraction patterns in the data set (Table 1 ▸). Remarkably, zones 4, 5 and 6 effectively represent a tilt series around the b* axis. If the angular relationship between these zones was known, the unit cell could have been determined using the Vainshtein method (Vainshtein, 1964 ▸). However, without knowledge of the zones’ mutual orientation, additional sections of reciprocal space (zones in different orientations) are needed to fix the cell. In this case, this is achieved by the first three zones and the zone number 7.

The zone index vectors of the patterns forming a Vainshtein tilt series are coplanar. Therefore, a unit cell generally cannot be determined from a set of zone patterns forming a Vainshtein tilt series (unless information on the mutual orientation of the zones is provided). For this reason, when dealing with an unknown unit cell, a series of patterns with a similar basis vector should be avoided.

**Figure 8:**
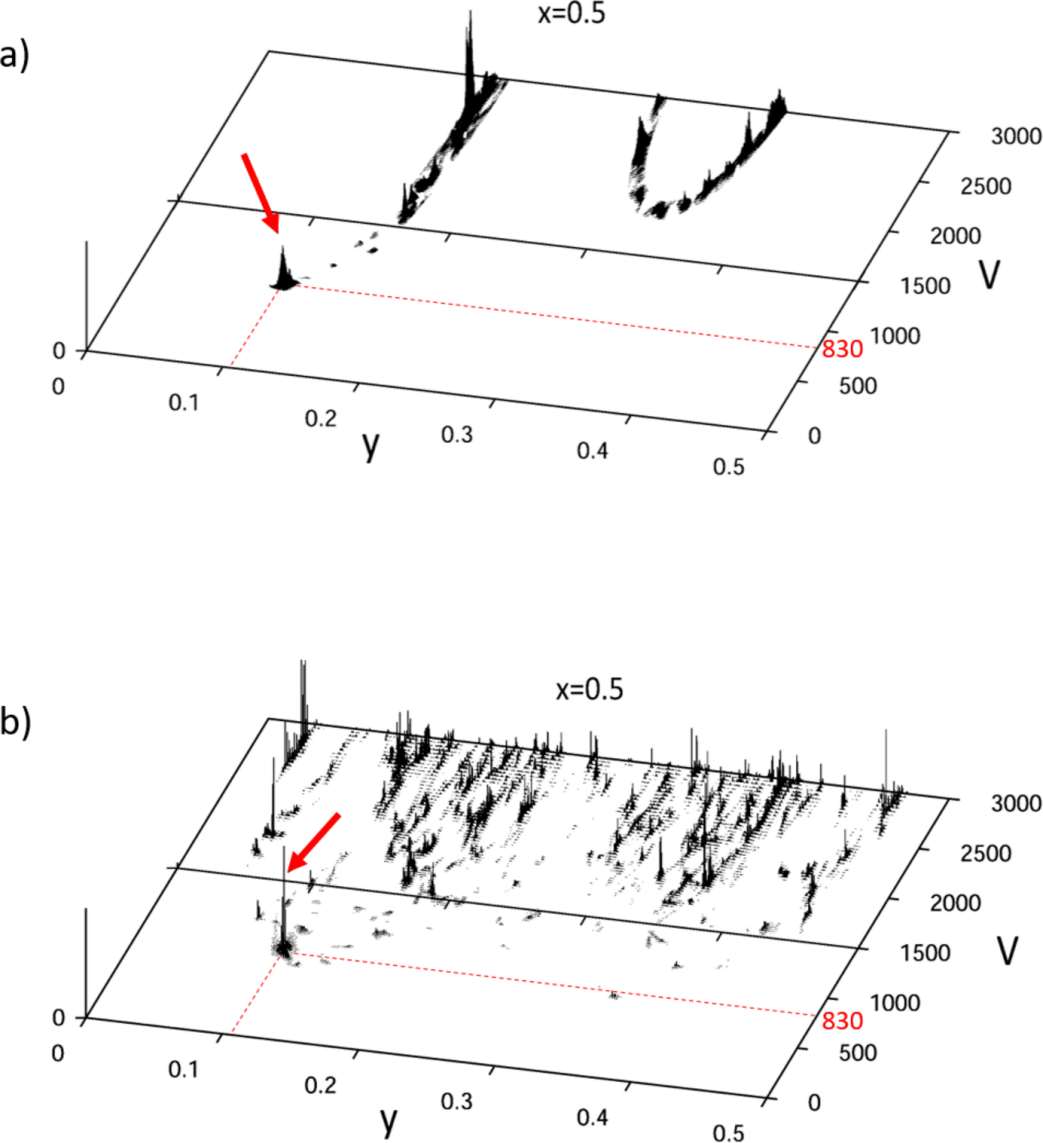
Inverse FOM surface (1/*R* values) within the (*y*, *V*) plane for *x* = 0.5 for 2D scan using all seven patterns (*a*) and (*b*) 3D search with five high-index patterns. The sharp peak at the volume of 830 Å^3^ corresponding to the best solution found is marked by red arrows. More solutions will emerge with increase in volume. The correct solution should have a high prominence at reasonably low volume values.

The incorporation of the long axes and low-index [001] zone with high symmetry (*cmm*), and correspondingly the reduction of the search space to 2D [Fig. 5 ▸(*c*)] significantly helps the algorithm to find the correct solution. A plane in the 2D solution space is shown in Fig. 8 ▸(*a*). Here, ‘inverse FOM’ *F*_inv_ = 1/*R* values are plotted in the (*y*, *V*) plane for *x* = 0.5. The sharp peak at the volume of 830 Å^3^ corresponds to the best solution found.

Practically, main zones with long axes are rarely present in a dataset for the reasons outlined above. We therefore repeated the unit-cell determination, with the [001] zone being excluded. In the absence of the zone number 7, zone number 4 with symmetry 2*mm* was chosen by the program to serve as the initial zone, still running a 2D search. The clipped dataset of six patterns produced the best solution (*R* = 0.55) with a primitive unit cell with the parameters *a* = 3.76, *b* = 15.23, *c* = 15.50 Å, α = 112.3°, β = 93.3°, γ = 93.5°, *V* = 816.1 Å^3^. The Delaunay-reduced *A*-centred cell had the parameters of *a* = 3.76, *b* = 25.52, *c* = 17.12 Å, α = 88.89°, β = 96.10°, γ = 89.93°. The subsequent transformation (as discussed above) resulted in the *C*-centred cell with *a* = 17.41, *b* = 25.52, *c* = 3.76 Å, β = 96.10°, matching the expected values. Thus, the dataset without the main zone also produced a correct unit-cell basis.

To enforce a 3D search with fewer patterns, we further reduced the dataset and removed pattern number 4 previously picked as the initial zone. The obtained dataset only contained five high-index zones. The best solution (*R* = 0.50) had a primitive unit cell, with parameters of *a* = 3.77, *b* = 15.23, *c* = 15.47 Å, α = 112.4°, β = 93.3°, γ = 93.7°, still matching the expected values.

The task of determining the correct set of lattice parameters is essentially equivalent to identifying the appropriate maximum on the 1/*R* surface. The figure-of-merit plane containing the optimal solution is shown in Fig. 8 ▸(*b*). While many intense peaks appear at high-volume values, the correct solution corresponds to the prominent peak at the smallest reasonable volume (*V* = 830 Å^3^). With the expulsion of the low-index and high-symmetry zones, the dimensionality of the search becomes higher, and the surface becomes noisy. These factors determine the success of the search routine.

### 3.2. Lysozyme

We then moved on to a serial crystallography dataset of lysozyme. These data presented a particular challenge for several reasons. (i) The experimental setup produced patterns with elliptical distortion of 2.3%, with the long axis at an angle of 85° with respect to the horizontal axis (Bücker *et al.*, 2021 ▸; Brázda *et al.*, 2022 ▸). However, this distortion is constant and hence could be corrected for by approximately assuming a camera length increased by 2% along the vertical axis. (ii) The long unit-cell axes of the protein crystals, in conjunction with peak broadening due to mosaicity, finite beam coherence and a large detector pixel size (9-pixel peak distance along a* and b*) yield a low sampling of the diffraction patterns, limiting the accuracy of vector length measurements, and therefore the performance of the GM algorithm.

**Figure 9:**
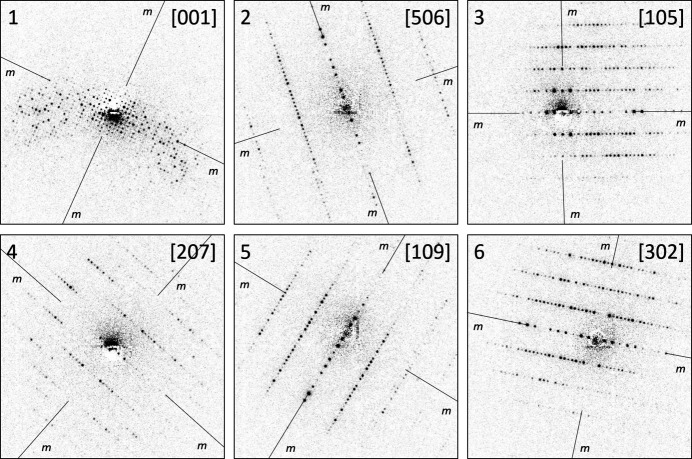
Random orientation zone patterns of lysozyme used for lattice parameters determination. Background subtraction as described by Bücker *et al.* (2021 ▸) has been applied. Vertical mirror planes are indicated in all patterns.

We initially selected six prominent zones with high apparent symmetry (Table 3 ▸, Fig. 9 ▸). One of these zones was the [001] pattern, with fourfold symmetry. The other patterns (numbers 2–6, Table 3 ▸) contained a main axis with evident extinctions, and all had symmetry 2*mm*. Although the experimentally measured angles deviated from 90° (87.796° in pattern number 1), we fixed them to 90°, as dictated by the symmetry, to boost the performance of the algorithm.

Selection of these zones would be a typical strategy for pattern selection in a situation without any prior knowledge of the cell metric. Patterns 2–6 are likely to represent a tilt series around the main axis; therefore, these alone cannot fix a lattice. Here, the main [001] zone is essential for the unit-cell determination.

**Figure 10:**
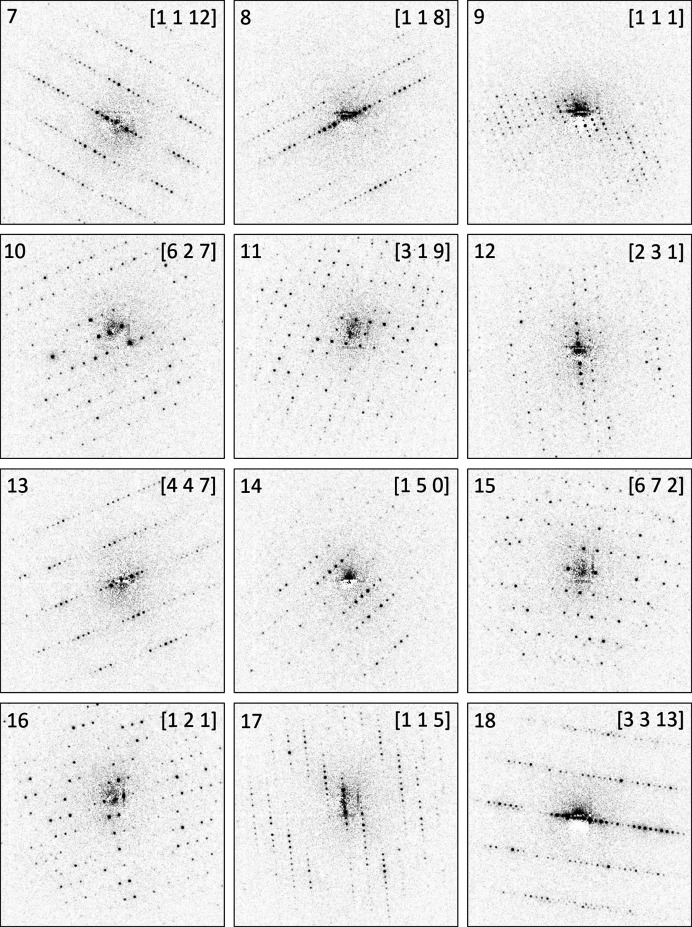
Additional ED zone patterns of lysozyme, indexed based on the found unit-cell metric.

The incorporation of the [001] zone initiated a 1D search, thanks to its fourfold symmetry. The solutions are given in Table 4 ▸. The best solution had a tetragonal metric with *a* = 79.06, *c* = 38.22 Å, matching the expected parameters very well. With these parameters, first the initial set of patterns could be indexed (patterns 1–6). The first pattern was indexed as [001], as expected; the other patterns all contain the 010 axis as a common axis and form a tilt series (Table 3 ▸). These lattice parameters were used to index an additional 12 patterns with symmetry *p*1, which were selected from the pattern pool, but not used for the lattice parameters determination. The results of the indexing procedure are shown in Fig. 10 ▸.

During the first run, we imposed tetragonal symmetry on the main zone and thus initiated 1D search. We then decided to increase the dimensionality of the search. A simple exclusion of the main [001] zone would leave us with five zone patterns forming a tilt series (patterns 2–6). To fix the lattice, we added the [111] zone (No. 9, Fig. 10 ▸). This set of zone patterns initiated a 2D scan. The best solution with a figure of merit of 0.6 had unit-cell parameters of *a* = 38.48, *b* = 78.65, *c* = 78.99 Å, α = 90.0°, β = 90.0°, γ = 91.4° and a volume of 238994.4 Å^3^, again close to the expected tetragonal metric.

Alternatively, we retained the main [001] in the dataset, set the angle between the vectors to the measured value of 87.796°, and then performed cell determination using the six patterns (1–6). The symmetry of the main zone was classified as *cmm*, with |**a**| = |**b**|, initiating a 2D search [Fig. 5 ▸(*c*)]. The best solution, with a figure of merit of 1.01, produced a somewhat distorted unit cell but with a still recognizable metric: *a* = 34.52, *b* = 79.24, *c* = 79.24 Å, α = 92.0°, β = 93.1°, γ = 93.1°.

### 3.3. GRGDS

**Figure 11:**
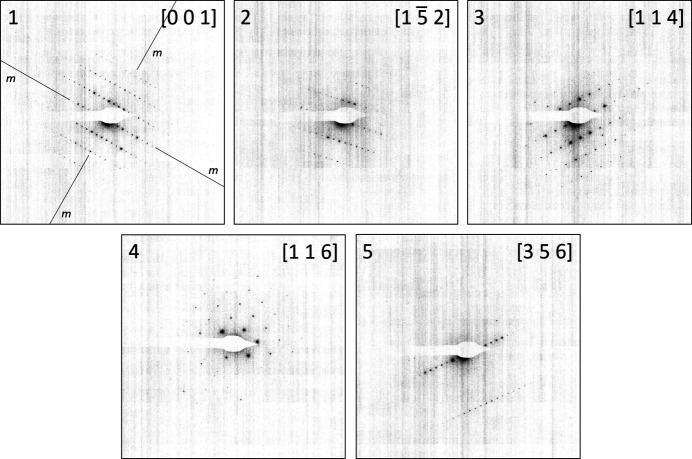
ED zone patterns of GRGDS peptide used for the lattice parameters determination. Vertical mirror planes are indicated in the first pattern.

We then moved on to a material with unknown crystal structure to see whether our procedure could give a reasonable suggestion for a unit-cell metric. Five patterns with a clear periodic pattern were selected as input for *PIEP* (Fig. 11 ▸). One of the patterns had symmetry *cmm*, all others had *p*1, and no extinctions were seen in the patterns. Vectors determined from all five patterns (Table 5 ▸) were input into *PIEP*. A protocol of the interactive session in the program is given in Section S5.2 in the supporting information.

The volume search range was set to [0 1500], the maximal limit being slightly larger than the doubled molecular volume. The pattern with symmetry *cmm* was selected as the a*–b*-defining plane, initiating 2D search. The best five solutions are presented in Table 6 ▸. The solution with the best figure of merit had a cell volume of 1195.6 Å^3^, matching the expected volume of two GRGDS molecules (2 × 585 = 1170 Å^3^). Delaunay reduction suggested a monoclinic *A*-centred unit cell with the lattice parameters of *a* = 19.466, *b* = 4.4446, *c* = 28.6756 Å, α = 90.02°, β = 105.47°, γ = 90.00°, which was transformed to standard settings using the transformation matrix [001; 010; 100]. The final unit cell is monoclinic *C*-centred with the lattice parameters of *a* = 28.6756, *b* = 4.4446, *c* = 19.466 Å, α = 90.00°, β = 105.47°, γ = 90.02°, and a volume of 2391.1 Å^3^. The unit cell should then contain four molecules of GRGDS. As GRGDS is a chiral molecule, the only possible space group would be the Sohncke *C*2 group (No. 5) with *Z* = 4, *Z*′ = 1.

The obtained unit cell was used to index the five zones. The indices of the reflections are given in Table 5 ▸. The first pattern was indexed as the main [001] zone, with the vectors having 200 and 110 indices, reflections 100 and 010 being absent due to the *C*-centring reflections condition: *h* + *k* = 2*n*. All other zones had relatively high indices. We tried to exclude the *cmm* zone from the dataset and run a 3D search; however, this did not produce any reasonable solution.

Recently, we determined the crystal structure of GRGDS from 3D ED data. The structure crystallizes in the *C*2 space group and forms a co-crystal with tri­fluoro­acetic acid (TFA). The lattice parameters, *a* = 29.231, *b* = 4.546, *c* = 19.640 Å and β = 106.70°, align closely with those determined using *PIEP*.

## 4. Discussion

The GM algorithm requires a few zonal patterns with the highest symmetry and the shortest vectors between the reflections as input. In this study, we manually selected patterns matching these criteria. Obviously, manually searching through a dataset containing thousands of patterns is impractical, but this procedure can be easily automated. The initial step in serial data reduction involves extracting the coordinates of the peaks from the patterns. One possible approach to select the most prominent zonal patterns involves autocorrelation of the peak positions, extracting the two shortest linearly independent vectors, constructing a 2D net from these basis vectors, and comparing the positions of the nodes of the created lattice with the initial peak positions on the pattern. If these match, it is likely that a regular 2D net of reflections is present. However, if many unmatched positions exist, the pattern may represent a high-index section of reciprocal space. The low-index pattern can then be further sorted based on the length of the vectors it contains. Alternatively, given the large number of patterns and the ease of generating ED patterns from a given structure, machine learning approaches could likely be applied to the task of selecting the most prominent zonal patterns.

The performance of the GM algorithm is driven by its intelligent incorporation of symmetry detected in ED patterns and by its code being specially optimized for speed, enabling even 3D searches to be completed within mere seconds. High symmetry reduces the dimensionality of the scan, thereby decreasing the solution space and, consequently, the computation time.

Finding the correct solution essentially involves identifying a prominent maximum within the solution space or plane (Fig. 8 ▸). The default volume fraction increment of 0.25 results in a relatively coarse scan grid. If finding a stable solution proves unsuccessful, the increment can be reduced, and the scan repeated within a smaller volume range. The target volume can be estimated either from the higher Laue zones present in the patterns or from the estimated molecular volume, as demonstrated above.

Naturally, a noisy search space (surface) will destabilize the procedure. Therefore, the errors in the data caused by diverse physical factors, such as distortion in diffraction patterns or slight off-tilt of zone orientations, are critical for the performance.

Typical issues arising in usage of the GM algorithm and how to handle them are as follows:

(i) Alien pattern(s) from *e.g.* contaminants or different polymorphs hidden among regular patterns, which will erroneously trigger rejection of correct solutions; suspicious patterns hence need to be removed.

(ii) Inaccurate geometry of the vectors in the diffraction patterns, *e.g.* due to elliptical distortion. In this case, one should increase the error margins on the diffraction pattern vectors or apply corrections for known distortions (Bücker *et al.*, 2021 ▸; Brázda *et al.*, 2022 ▸).

(iii) Incorrect search range (*z* search) of the unit-cell volume. If no satisfactory unit cell is found, one should increase the search range of the volume.

(iv) Symmetry of the zone [001] is overestimated due to a pseudo-mirror plane, triggering the 1D or 2D scan strategies. If this is suspected, one should enforce the general 3D scan strategy via a dedicated option, or choose a different pattern to define the initial [001] zone.

Table 7 ▸ lists all expected and determined unit-cell parameters. For both monoclinic centred structures, CuPcCl_16_ and GRGDS, the corresponding primitive unit cell is provided. For GRGDS, the primitive unit cell was calculated from the experimentally obtained *C*-centred lattice using the transformation matrix [010; 0.5 0.5 0; 001].

For both small-molecule compounds, the average error in the angles of the determined unit cell is below 1°, with a maximum of 1.2° for γ of GRGDS. For the lysozyme structure, which has large lattice parameters and lower accuracy in experimentally measured angles (not precisely in-zone patterns), the error in the angle is significantly higher, reaching up to 3°.

Estimating the accuracy of the length parameters is more challenging. The absolute lengths of the unit-cell vectors are often influenced by camera constant errors. For this reason, *PIEP* separates the camera constant and the ratios of vector lengths when calculating the figure of merit for a solution. We have chosen to adopt the same approach. To assess the accuracy of the obtained unit-cell metrics relative to expected values, while factoring out camera constant errors, we use the following figure of merit:
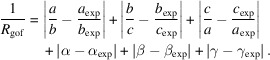


It is possible to develop a weighting scheme for length and angular errors and to develop a qualitative criterion for 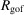 to correspond to a ‘correct’ unit cell. However, for simplicity, we decided to give equal weight to both parts, leaving this important aspect for further studies.

The *PIEP* program is written in legacy Fortran, which provides excellent performance and is still used in core components of many crystallographic programs and general scientific packages and provides a friendly text-based interactive interface. *PIEP* can be compiled without specific efforts on modern computing hardware running Windows, macOS or Linux, using *GCC* or Microsoft Fortran compilers. While the declining use of Fortran may be somewhat of a hurdle for integration with more contemporary computing environments, successful integrations of this type are not uncommon, such as *CCSL* (https://www.ill.eu/sites/ccsl/html/ccsldoc.html) integrated in *DASH* (David *et al.*, 2006 ▸).

In addition to an interfacing layer based on keystroke emulation (as implemented, for example, in the indexing sub-programs of *CrystFEL*; White *et al.*, 2012 ▸, 2016 ▸), wrapper codes such as *F2PY* (https://numpy.org/doc/stable/f2py/) could offer a promising approach for interfacing with Python. This could allow the integration of *PIEP* into serial electron crystallography packages such as *diffractem* (Bücker *et al.*, 2021 ▸) or *Instamatic* (Smeets *et al.*, 2018 ▸). Alternatively, and this is likely to be a more straightforward approach, the GM algorithm can be re-implemented within another software framework.

## 5. Conclusion

Here we present an old program *PIEP* (Program for Interpreting Electron diffraction Patterns) written by one of the authors, Gerhard Miehe, in the early 1990s. The program has a robust and well designed algorithm (the GM algorithm) for unit-cell parameters determination from a set of randomly oriented zonal ED patterns, a problem with renewed relevance in the context of the development of serial electron crystallography.

*PIEP* operates with zonal diffraction patterns, which are planar sections of reciprocal space with all reflections forming a regular 2D net. These patterns inherently lack 3D information, making them particularly challenging to index using existing X-ray serial crystallography approaches. We anticipate that these patterns can be automatically identified within an electron serial crystallography dataset and used to determine unit-cell parameters using the GM algorithm. These parameters can then be used to index the entire dataset.

We demonstrate the performance of *PIEP* for lattice parameters determination of known materials with moderate lengths of crystallographic axes, copper perchlorophthalocyanine (CuPcCl_16_) and lysozyme protein crystals, challenging the algorithm by long lattice vectors. We ran the program in low-dimensional search mode, corresponding to the symmetry of the material and increased the dimensionality of the search by expulsion of the high-symmetry zone patterns. In all situations, *PIEP* was able to reliably find the correct solution and index all provided zone patterns. Finally, we applied the procedure to unit-cell determination of the GRGDS peptide with unknown structure. Also, here, the algorithm gave a reasonable suggestion for the unit-cell metric, so that even the space group could be suggested.

Determining unit-cell parameters from a set of randomly oriented ED patterns is a bottleneck in the analysis of serial ED data. The GM algorithm could hence represent a working solution for this crucial step of the analysis pipeline.

## 6. Related literature

The following reference is cited in the supporting information: Kabsch (1993 ▸).

## 7. Program availability

The program is written in Fortran IV, can be compiled under Microsoft Windows, macOS and Linux, and runs in a terminal window with *ca* 130 commands available. The program as well as a list of commands is available at Zenodo: DOI 10.5281/zenodo.7859089. 

## Supplementary Material

Supporting information. DOI: 10.1107/S2053273325000300/lu5038sup1.pdf

## Figures and Tables

**Table 1:** ED zonal data of CuPcCl_16_ used for the lattice parameters determination procedure The zone index was determined based on the unit cell found.

No.	*d*_1_ (Å)	*d*_2_ (Å)	φ (°)	Symmetry	*hkl* (*d*_1_)	*hkl* (*d*_2_)	Zone index
1	7.59	3.75	93.3	*p*1	130	111	[314]
2	7.59	3.55	74.5	*p*1	130	111	[312]
3	8.51	2.62	95.6	*p*1	200	171	[017]
4	12.76	2.97	89.4	2*mm*	020	401	[104]
5	12.75	2.65	96.5	*p*1	020	511	[105]
6	12.75	2.15	85.9	*p*1	020	711	[107]
7	14.15	14.45	68.0	*cmm*	110	110	[001]

**Table 2:** Best solutions for the lattice parameters determination of CuPcCl_16_

No.	*R*	*a* (Å)	*b* (Å)	*c* (Å)	α (°)	β (°)	γ (°)	*V* (Å^3^)
1	0.85	3.82	15.28	15.60	111.7	93.1	92.9	841.8
2	1.03	4.01	15.27	15.58	112.0	90.7	91.5	884.2
3	1.08	3.91	15.31	15.58	68.1	89.8	85.6	862.7
4	1.32	3.92	15.34	15.58	111.8	90.7	95.7	841.8
5	1.46	4.54	15.26	15.62	111.9	94.2	90.0	1000.0

**Table 3:** ED zonal data used for the lattice parameters determination of lysozyme The zone index was determined based on the unit cell found.

No.	*d*_1_ (Å)	*d*_2_ (Å)	φ (°)	Symmetry	*hkl* (*d*_1_)	*hkl* (*d*_2_)	Zone index
1	79.06	79.06	90	4	010	100	[001]
2	77.48	6.46	90	2*mm*	010	605	[506]
3	78.99	14.68	90	2*mm*	010	501	[105]
4	77.12	9.53	90	2*mm*	010	702	[207]
5	78.20	8.52	90	2*mm*	010	901	[109]
6	79.64	12.09	90	2*mm*	010	203	[302]

**Table 4:** Best solutions for the lattice parameters determination of lysozyme, 1D search

No.	*R*	*a* (Å)	*b* (Å)	*c* (Å)	α (°)	β (°)	γ (°)	*V* (Å^3^)
1	0.80	38.22	79.06	79.06	90.0	90.0	90.0	238898.3
2	0.91	28.93	79.06	79.06	90.0	90.0	90.0	180852.8
3	0.94	44.48	79.06	79.06	90.0	90.0	90.0	278068.6
4	0.99	47.99	79.06	79.06	90.0	90.0	90.0	300000.0
5	1.02	30.43	79.06	79.06	90.0	90.0	90.0	190241.3

**Table 5:** ED zonal data used for the lattice parameters determination of GRGDS The zone index was determined based on the unit cell found.

No.	*d*_1_ (Å)	*d*_2_ (Å)	φ (°)	Symmetry	*hkl* (*d*_1_)	*hkl* (*d*_2_)	Zone index
1	13.82	4.39	80.9	*cmm*	200	110	[001]
2	12.94	3.91	85.6	*p*1	201	112	[152]
3	7.10	4.42	80.8	*p*1	401	110	[114]
4	4.76	4.40	80.5	*p*1	601	110	[116]
5	12.99	1.47	89.1	*p*1	201	132	[356]

**Table 6:** Best solutions for the lattice parameters determination of GRGDS

No.	*R*	*a* (Å)	*b* (Å)	*c* (Å)	α (°)	β (°)	γ (°)	*V* (Å^3^)
1	0.84	4.44	14.51	19.47	105.3	90.0	98.8	1195.6
2	0.99	4.44	14.49	20.47	105.0	90.0	98.8	1257.4
3	1.14	4.44	14.42	21.53	76.1	90.0	81.2	1322.4
4	1.16	7.82	13.21	14.36	86.5	74.2	76.9	1390.8
5	1.17	4.44	14.52	18.98	74.6	90.0	81.2	1165.8

**Table 7:** List of expected and determined unit-cell parameters

	No. of zones	*a* (Å)	*b* (Å)	*c* (Å)	α (°)	β (°)	γ (°)	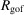
CuPcCl_16_		3.833	15.688	15.688	111.39	92.84	92.84	–
3D	7	3.82	15.28	15.60	111.7	93.1	92.9	1.50
2D, *cmm*	7	3.82	15.44	15.44	111.8	92.7	92.7	1.34
2D, 2*mm*	6	3.76	15.23	15.50	112.3	93.3	93.5	0.48
3D	5	3.77	15.23	15.47	112.4	93.3	93.7	0.42
								
Lysozyme		77.51	77.51	37.42	90	90	90	–
1D, 4*mm*	6	79.06	79.06	38.22	90	90	90	289
2D, 2*mm*	6	38.48	78.65	78.99	90.0	90.0	91.4	0.23
2D, *cmm*	6	34.52	79.24	79.24	92.0	93.1	93.1	0.09
								
GRGDS		4.546	14.791	19.640	106.496	90	98.84	–
2D, *cmm*	5	4.44	14.51	19.47	105.3	90.0	98.8	0.76
