# Supplementary material for: Unit-cell parameters determination from a set of independent electron diffraction zonal patterns
Source: Acta Crystallogr A Found Adv. 2025 Jan 31;81(Pt 2):124–36. doi: 10.1107/S2053273325000300 (PMC11873814; doi:10.1107/S2053273325000300)
Supplement: Supplementary file 1 [file a-81-00124-sup1.pdf]

FOUNDATIONS
ADVANCES

Volume 81 (2025)

Supporting information for article:

Unit-cell parameter determination from a set of independent electron diffraction zonal patterns

Tatiana E. Gorelik, Gerhard Miehe, Robert Bücker and Kaname Yoshida

S1. Reduction of electron diffraction patterns

S1.1. Manual selection of basis vectors

A script for basis vectors extraction was written in MATLAB. The script allows the user to select three reflections corresponding to the vectors $\boldsymbol{r_1}$, $\boldsymbol{r_2}$, $\boldsymbol{r_1}+\boldsymbol{r_2}$, thus defining a 2D lattice. The vicinity of the positions of the lattice nodes is scanned for a reflection. If one is found, it is added to a pool of spots, which is then used for the least-square refinement of the basis vectors. As required by PIEP, the lengths of $\boldsymbol{r_1}$, $\boldsymbol{r_2}$ and an angle φ between the vectors were calculated.
The script is available at ZENODO 10.5281/zenodo.7863651

S1.2. Auto correlation of patterns

As an alternative to directly picking peak distances from diffraction patterns, autocorrelation patterns can be computed from either the raw pixel values or the Bragg spot positions found by a peak-finding routine. Such a pattern can be regarded as a histogram of all pair-wise peak distances and hence contains all peaks of the original pattern, but rejects noise in the images, and yields better-defined spots that allow for a more accurate extraction of vectors, especially if sub-pixel accurate peak-finding is used (Jiang et al., 2011; Kabsch 1993). Also, autocorrelation patterns are inherently unaffected by systematic absences, which further simplifies the unambiguous definition of low-resolution vectors. Especially for noisy or under-sampled data, defining the input vectors for PIEP using an autocorrelation pattern instead of the direct pattern may be advantageous. In Figure S1, an example is shown, where the package *diffractem* (Bücker et al., 2021) was used for computation of the autocorrelation pattern from previously found peaks.
An inherent problem of the autocorrelation method is the propensity for displaying spurious peaks, which can arise due to Bragg spots from an additional crystal, or traces of higher-index Laue zones.

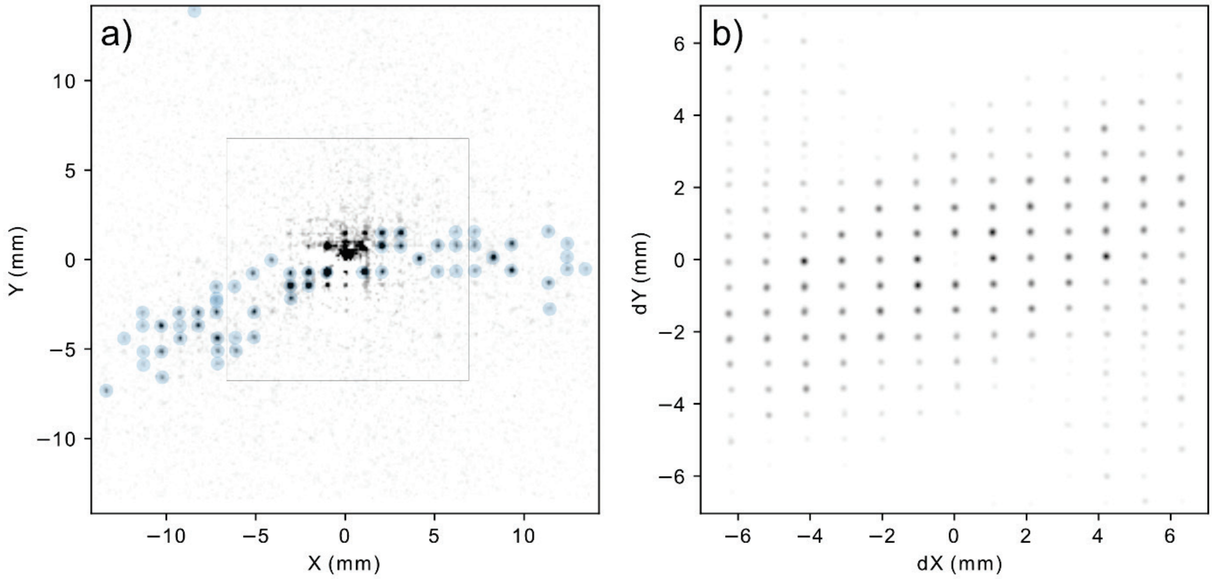

Figure S1: Autocorrelation pattern. A) Typical diffraction pattern from lysozyme with Bragg reflections found by a peak-finding algorithm overlaid as blue dots, assuming the experimental detector distance of 1580 mm. B) Autocorrelation pattern computed from the found spot positions in a) within a pair-wise distance range as indicated by the box overlay in a).

S2. List of typical commands used for communication with PIEP

pg – "pattern get" – get a pattern from the file sad.dat into the working area.

ap – "append pattern" – append the pattern to the list in the working area.

pc – "prepare cell" – set the parameters for a cell determination run.

dc – "determine cell" – run the cell search procedure.

de – Delaunay reduction.

mv – matrix- and vector operations.

m1 – read matrix 1.

ma – apply matrix 1 to current cell parameters.

i – index a given pattern with the unit cell parameters found.

ax – exclude a pattern from a list.

S3. PIEP run for unit cell parameters determination for $CuPcCl_{16}$

S3.1. Input file definition (sad.dat), input file (sad.dat) for $CuPcCl_{16}$

This file contains measured values for the camera constant, the measured lengths of two vectors in each diffraction pattern and angles between the vectors in the form:

Line 1:	Pattern running number | title
Line 2:	Camera constant (here in [pix·Å])| error of the camera constant (here 5%) | measured length of the vector $\boldsymbol{r_1}$ (here in [pix]) | error of $\boldsymbol{r_1}$ (here 3%) | measured length of the vector $\boldsymbol{r_2}$ (here in [pix]) | error of $\boldsymbol{r_2}$ (here 3%)

Line 3:	Measured length of the vector $\boldsymbol{r_{12}}=\boldsymbol{r_1}-\boldsymbol{r_2}$ (here in [pix]), is not essential if the angle between the two vectors is provided | error of $\boldsymbol{r_{12}}$ (here 3%) | measured angle φ, [°] between $\boldsymbol{r_1}-\boldsymbol{r_2}$ | error of φ, [°] (here 3%) | optional: measured length of the vector $\boldsymbol{r_{21}}=\boldsymbol{r_1}+\boldsymbol{r_2}$ | optional: error of $\boldsymbol{r_{21}}$ | optional: radius of zero-order Laue zone
Line 4:	Optional: difference radius 1st order - radius zero order Laue zone | optional: type of specimen holder | optional: goniometer angle 1 | optional: goniometer angle 2 | TEM high voltage in V

All numeric SAD data except for high voltage and goniometer angles require an estimated error. d-values and wavelength are calculated in Å, , high voltage must be given in V (not kV!). The units of SAD data are related to the units of the camera constant (see section 6 below).

Input file (sad.dat) for $CuPcCl_{16}$

```
   1 CuPc pattern 19
1100.0000  55.0000 145.0000   4.3500 293.2600   8.7978
 334.5031   0.0000  93.2800   2.5000     0.00     0.00     0.00
     0.00     5.00     0.00     5.00  300000.
   2 CuPc pattern 20
1100.0000  55.0000 144.8900   4.3467 310.2700   9.3081
 305.4153   0.0000  74.5300   2.5000     0.00     0.00     0.00
     0.00     5.00     0.00     5.00  300000.
   3 CuPc pattern 24
1100.0000  55.0000 129.2900   3.8787 419.2200  12.5766
 450.5573   0.0000  95.5800   2.5000     0.00     0.00     0.00
     0.00     5.00     0.00     5.00  300000.
   4 CuPc pattern 29
1100.0000  55.0000  86.2100   2.5863 370.6000  11.1180
 379.5663   0.0000  89.3670   2.5000     0.00     0.00     0.00
     0.00     5.00     0.00     5.00  300000.
   5 CuPc pattern 30
1100.0000  55.0000  86.2600   2.5878 414.9200  12.4476
 433.3040   0.0000  96.5400   2.5000     0.00     0.00     0.00
     0.00     5.00     0.00     5.00  300000.
   6 CuPc pattern 31
1100.0000  55.0000  86.2600   2.5878 511.5500  15.3465
 512.6692   0.0000  85.9100   2.5000     0.00     0.00     0.00
     0.00     5.00     0.00     5.00  300000.
   7 CuPc pattern 32
1100.0000  55.0000  77.7300   2.3319  76.1300   2.2839
  86.0699   0.0000  68.0200   2.5000     0.00     0.00     0.00
     0.00     5.00     0.00     5.00  300000.
   8 CuPc pattern 32i
1100.0000  55.0000  76.9300   2.3079  76.9300   2.3079
  86.0699   0.0000  68.0200   2.5000     0.00     0.00     0.00
     0.00     5.00     0.00     5.00  300000.
END$
```

S3.2. Communication protocol with PIEP

Commands are printed in red.

```
##################################
=======       P I E P      =======
======= VERSION 14-jun-17 =======
```

```
                #################################

 default parameters from file? (def.=yes)

 parameter-file piep.par            ? (blank), otherwise name

 ------------------------------------------------------------------------
  cell parameter file assigned: cell.dat             ,     1 sets
  1st set read, unit: 20, file: cell.dat
 ------------------------------------------------------------------------
  SAD data file: unit 30, file: sad.dat              ,     7 sets
  1st set loaded
 ------------------------------------------------------------------------

dummy
 dir.lc.:  10.0000  10.0000  10.0000  90.00  90.00  90.00, V(P):  1000.0 cub.
 rec.lc.: 0.100000 0.100000 0.100000  90.00  90.00  90.00, SG. P (***)

 CuPc pattern 19
   r1: 145.00 +- 4.35; ang.:   93.28 +-  2.50;   L0:    0.00; wgt(angle): 0.6
   r2: 293.26 +- 8.80; c.c.: 1100.00 +- 55.00; L1-0:    0.00; wgt(r1/r2): 0.8
   r3: 334.50 +- 0.00; volt:     300000.     ; V(P):     0.0; wgt(c.c.) : 0.3
 mul.:    2, cent.:P, rl: 0, rewind: Y, nr:   199
*
pg
 consec. # of data set? 0 or <0: next set (   2 )
1
dummy
 dir.lc.:  10.0000  10.0000  10.0000  90.00  90.00  90.00, V(P):  1000.0 cub.
 rec.lc.: 0.100000 0.100000 0.100000  90.00  90.00  90.00, SG. P (***)

 CuPc pattern 19
   r1: 145.00 +- 4.35; ang.:   93.28 +-  2.50;   L0:    0.00; wgt(angle): 0.6
   r2: 293.26 +- 8.80; c.c.: 1100.00 +- 55.00; L1-0:    0.00; wgt(r1/r2): 0.8
   r3: 334.50 +- 0.00; volt:     300000.     ; V(P):     0.0; wgt(c.c.) : 0.3
 mul.:    2, cent.:P, rl: 0, rewind: Y, nr:   199
*
ap
  free:      1  2  3  4  5  6  7  8  9 10 11 12 13 14 15 16 17 18 19 20
 which number?
1
   #         d1   s/d(%)    d2  s/d(%)   d3     ang. sig.     V      d-m  seq.
   1        7.5862 3.00   3.7509 3.00   3.2885  93.3  2.5      0.0   5.339  1
*
pg
 consec. # of data set? 0 or <0: next set (   2 )
2
dummy
 dir.lc.:  10.0000  10.0000  10.0000  90.00  90.00  90.00, V(P):  1000.0 cub.
 rec.lc.: 0.100000 0.100000 0.100000  90.00  90.00  90.00, SG. P (***)

 CuPc pattern 20
   r1: 144.89 +- 4.35; ang.:   74.53 +-  2.50;   L0:    0.00; wgt(angle): 0.6
   r2: 310.27 +- 9.31; c.c.: 1100.00 +- 55.00; L1-0:    0.00; wgt(r1/r2): 0.8
   r3: 305.42 +- 0.00; volt:     300000.     ; V(P):     0.0; wgt(c.c.) : 0.3
 mul.:    2, cent.:P, rl: 0, rewind: Y, nr:   199
*
ap
  free:      2  3  4  5  6  7  8  9 10 11 12 13 14 15 16 17 18 19 20
 which number?
2
   #         d1   s/d(%)    d2  s/d(%)   d3     ang. sig.     V      d-m  seq.
   1        7.5862 3.00   3.7509 3.00   3.2885  93.3  2.5      0.0   5.339  1
   2        7.5920 3.00   3.5453 3.00   3.6017  74.5  2.5      0.0   5.285  2
*
pg
 consec. # of data set? 0 or <0: next set (   3 )
3
dummy
 dir.lc.:  10.0000  10.0000  10.0000  90.00  90.00  90.00, V(P):  1000.0 cub.
```

```
 rec.lc.: 0.100000 0.100000 0.100000  90.00  90.00  90.00, SG. P (***)

 CuPc pattern 24
   r1: 129.29 +- 3.88; ang.:   95.58 +-  2.50;   L0:    0.00; wgt(angle): 0.6
   r2: 419.22 +-12.58; c.c.: 1100.00 +- 55.00; L1-0:    0.00; wgt(r1/r2): 0.8
   r3: 450.56 +- 0.00; volt:     300000.     ; V(P):     0.0; wgt(c.c.) : 0.3
 mul.:    2, cent.:P, rl: 0, rewind: Y, nr:   199
*
ap
  free:      3   4   5   6   7   8   9 10 11 12 13 14 15 16 17 18 19 20
 which number?
3
  #         d1   s/d(%)    d2  s/d(%)    d3     ang. sig.     V      d-m  seq.
  1        7.5862 3.00   3.7509 3.00   3.2885  93.3  2.5      0.0  5.339  1
  2        7.5920 3.00   3.5453 3.00   3.6017  74.5  2.5      0.0  5.285  2
  3        8.5080 3.00   2.6239 3.00   2.4414  95.6  2.5      0.0  4.736  3
*
pg
 consec. # of data set? 0 or <0: next set (   4 )
4
dummy
 dir.lc.:  10.0000  10.0000  10.0000  90.00  90.00  90.00, V(P):  1000.0 cub.
 rec.lc.: 0.100000 0.100000 0.100000  90.00  90.00  90.00, SG. P (***)

 CuPc pattern 29
   r1:  86.21 +- 2.59; ang.:   89.37 +-  2.50;   L0:    0.00; wgt(angle): 0.6
   r2: 370.60 +-11.12; c.c.: 1100.00 +- 55.00; L1-0:    0.00; wgt(r1/r2): 0.8
   r3: 379.57 +- 0.00; volt:     300000.     ; V(P):     0.0; wgt(c.c.) : 0.3
 mul.:    2, cent.:P, rl: 0, rewind: Y, nr:   199
*
ap
  free:      4   5   6   7   8   9 10 11 12 13 14 15 16 17 18 19 20
 which number?
4
  #         d1   s/d(%)    d2  s/d(%)    d3     ang. sig.     V      d-m  seq.
  1        7.5862 3.00   3.7509 3.00   3.2885  93.3  2.5      0.0  5.339  2
  2        7.5920 3.00   3.5453 3.00   3.6017  74.5  2.5      0.0  5.285  3
  3        8.5080 3.00   2.6239 3.00   2.4414  95.6  2.5      0.0  4.736  4
  4       12.7595 3.00   2.9682 3.00   2.8980  89.4  2.5      0.0  6.154  1
*
pg
 consec. # of data set? 0 or <0: next set (   5 )
5
dummy
 dir.lc.:  10.0000  10.0000  10.0000  90.00  90.00  90.00, V(P):  1000.0 cub.
 rec.lc.: 0.100000 0.100000 0.100000  90.00  90.00  90.00, SG. P (***)

 CuPc pattern 30
   r1:  86.26 +- 2.59; ang.:   96.54 +-  2.50;   L0:    0.00; wgt(angle): 0.6
   r2: 414.92 +-12.45; c.c.: 1100.00 +- 55.00; L1-0:    0.00; wgt(r1/r2): 0.8
   r3: 433.30 +- 0.00; volt:     300000.     ; V(P):     0.0; wgt(c.c.) : 0.3
 mul.:    2, cent.:P, rl: 0, rewind: Y, nr:   199
*
ap
  free:      5   6   7   8   9 10 11 12 13 14 15 16 17 18 19 20
 which number?
5
  #         d1   s/d(%)    d2  s/d(%)    d3     ang. sig.     V      d-m  seq.
  1        7.5862 3.00   3.7509 3.00   3.2885  93.3  2.5      0.0  5.339  3
  2        7.5920 3.00   3.5453 3.00   3.6017  74.5  2.5      0.0  5.285  4
  3        8.5080 3.00   2.6239 3.00   2.4414  95.6  2.5      0.0  4.736  5
  4       12.7595 3.00   2.9682 3.00   2.8980  89.4  2.5      0.0  6.154  1
  5       12.7521 3.00   2.6511 3.00   2.5386  96.5  2.5      0.0  5.833  2
*
pg
 consec. # of data set? 0 or <0: next set (   6 )
6
dummy
 dir.lc.:  10.0000  10.0000  10.0000  90.00  90.00  90.00, V(P):  1000.0 cub.
 rec.lc.: 0.100000 0.100000 0.100000  90.00  90.00  90.00, SG. P (***)
```

```
 CuPc pattern 31
   r1:  86.26 +- 2.59; ang.:   85.91 +-  2.50;   L0:    0.00; wgt(angle): 0.6
   r2: 511.55 +-15.35; c.c.: 1100.00 +- 55.00; L1-0:    0.00; wgt(r1/r2): 0.8
   r3: 512.67 +- 0.00; volt:     300000.     ; V(P):     0.0; wgt(c.c.) : 0.3
 mul.:    2, cent.:P, rl: 0, rewind: Y, nr:   199
*
ap
  free:      6  7  8  9 10 11 12 13 14 15 16 17 18 19 20
 which number?
6
  #         d1   s/d(%)    d2  s/d(%)    d3     ang. sig.     V      d-m  seq.
  1        7.5862 3.00   3.7509 3.00   3.2885  93.3  2.5      0.0  5.339  3
  2        7.5920 3.00   3.5453 3.00   3.6017  74.5  2.5      0.0  5.285  4
  3        8.5080 3.00   2.6239 3.00   2.4414  95.6  2.5      0.0  4.736  6
  4       12.7595 3.00   2.9682 3.00   2.8980  89.4  2.5      0.0  6.154  1
  5       12.7521 3.00   2.6511 3.00   2.5386  96.5  2.5      0.0  5.833  2
  6       12.7521 3.00   2.1503 3.00   2.1456  85.9  2.5      0.0  5.243  5
*
pg
 consec. # of data set? 0 or <0: next set (   7 )
7
dummy
 dir.lc.:  10.0000  10.0000  10.0000  90.00  90.00  90.00, V(P):  1000.0 cub.
 rec.lc.: 0.100000 0.100000 0.100000  90.00  90.00  90.00, SG. P (***)

 CuPc pattern 32
   r1:  77.73 +- 2.33; ang.:   68.02 +-  2.50;   L0:    0.00; wgt(angle): 0.6
   r2:  76.13 +- 2.28; c.c.: 1100.00 +- 55.00; L1-0:    0.00; wgt(r1/r2): 0.8
   r3:  86.07 +- 0.00; volt:     300000.     ; V(P):     0.0; wgt(c.c.) : 0.3
 mul.:    2, cent.:P, rl: 0, rewind: Y, nr:   199
*
ap
  free:      7  8  9 10 11 12 13 14 15 16 17 18 19 20
 which number?
7
  #         d1   s/d(%)    d2  s/d(%)    d3     ang. sig.     V      d-m  seq.
  1        7.5862 3.00   3.7509 3.00   3.2885  93.3  2.5      0.0  5.339  4
  2        7.5920 3.00   3.5453 3.00   3.6017  74.5  2.5      0.0  5.285  5
  3        8.5080 3.00   2.6239 3.00   2.4414  95.6  2.5      0.0  4.736  7
  4       12.7595 3.00   2.9682 3.00   2.8980  89.4  2.5      0.0  6.154  2
  5       12.7521 3.00   2.6511 3.00   2.5386  96.5  2.5      0.0  5.833  3
  6       12.7521 3.00   2.1503 3.00   2.1456  85.9  2.5      0.0  5.243  6
  7       14.1515 3.00  14.4490 3.00  12.7803  68.0  2.5      0.0 14.849  1
*
pc
  #         d1   s/d(%)    d2  s/d(%)    d3     ang. sig.     V      d-m  seq.
  1        7.5862 3.00   3.7509 3.00   3.2885  93.3  2.5      0.0  5.339  3
  2        7.5920 3.00   3.5453 3.00   3.6017  74.5  2.5      0.0  5.285  4
  3        8.5080 3.00   2.6239 3.00   2.4414  95.6  2.5      0.0  4.736  6
  4       12.7595 3.00   2.9682 3.00   2.8980  89.4  2.5      0.0  6.154  1
  5       12.7521 3.00   2.6511 3.00   2.5386  96.5  2.5      0.0  5.833  2
  6       12.7521 3.00   2.1503 3.00   2.1456  85.9  2.5      0.0  5.243  5
  7       14.1515 3.00  14.4490 3.00  12.7803  68.0  2.5      0.0 14.849  0

 a*,b*-defining:  7;
       sequence:       4;    5;    1;    2;    6;    3;

   1st: new a*,b* defining number (0: no changes)
   2nd: <0: enforce full grid, >0: * sigma (2.31mm) = "wall thickness"


 V(P)(min), V(P)(max)? (calc.: min:     0. max:     0., mean:     0.)
 def.:     0.     0.
0 1000
    763.  1000.
 factor for default increment (0.025), def.:1., max:6; <0 : increment

   0.025
```

```
      786 sets within  12 layers, p:  0.233 -  0.305
*
dc
  mult.<mu>: 2; <rl>: 0;  1st layer: V: 762.9; n:   56; p:  0.233
  ok?


      23 solutions stored,  R :     0.85 -   3.50, incl. equiv.:      219
      R      a      b      c      al     be     ga       x       y        V     int.
   1  0.85  3.82 15.28 15.60  111.7  93.1  92.9  0.300  0.200   841.8  1.00
   2  1.03  4.01 15.27 15.58  112.0  90.7  91.5  0.083  0.100   884.2  0.91
   3  1.08  3.91 15.31 15.58   68.1  89.8  85.6  0.100  0.300   862.7  0.99
   4  1.32  3.82 15.34 15.58  111.8  90.7  95.7  0.200  0.400   841.8  0.68
   5  1.46  4.54 15.26 15.62  111.9  94.2  90.0  0.250  0.000  1000.0  0.18
   6  1.50  3.63 15.37 15.59   68.0  88.8  83.2  0.100  0.500   801.3  0.76
   7  1.63  3.91 15.26 15.60   68.0  87.1  90.0  0.200  0.000   862.7  0.80
   8  1.77  4.42 15.28 15.68   67.9  83.6  87.2 -0.333  0.167   975.7  0.31
   9  1.84  4.54 15.28 15.59  111.8  91.7  92.8  0.167  0.167  1000.0  0.22
  10  1.87  4.11 15.31 15.58   68.1  89.5  85.4  0.083  0.300   906.3  0.31
  11  1.92  3.63 15.28 15.68   67.9  83.7  87.3 -0.400  0.200   801.3  0.27
  12  1.94  4.21 15.26 15.58  112.0  90.8  91.3  0.083  0.083   928.8  0.60
  13  1.97  4.11 15.26 15.62  111.9  93.8  90.0  0.250  0.000   906.3  0.45
  14  1.97  4.42 15.26 15.62   68.1  85.9  90.0  0.250  0.000   975.7  0.39
  15  2.13  3.82 15.34 15.61  111.5  93.5  95.7  0.400  0.400   841.8  0.28
  16  2.13  4.32 15.30 15.58   68.1  89.8  86.0  0.083  0.250   952.0  0.39
  17  2.16  4.32 15.26 15.62  111.9  94.0  90.0  0.250  0.000   952.0  0.14
  18  2.38  3.72 15.26 15.62   68.1  85.9  90.0  0.300  0.000   821.3  0.13
  19  2.43  4.32 15.28 15.59   67.9  87.7  87.3 -0.083  0.167   952.0  0.27
  20  2.48  4.21 15.33 15.61   67.8  86.7  84.7 -0.083  0.333   928.8  0.13
  21  2.74  4.54 15.28 15.71  111.4  97.2  92.8  0.500  0.167  1000.0  0.06
  22  2.85  4.54 15.34 15.59  111.6  92.0  95.7  0.250  0.333  1000.0  0.06
  23  3.50  3.63 15.37 15.65   67.6  84.6  83.2 -0.400  0.500   801.3  0.05
*
de
 one line: # of transf. to be loaded (0: list all transf.)
           write matrix? (0:no, >0:yes)
           minim. symmetry? (0(=def.):trk.,1:mcl.,2:orh.,3:tet.,4:hex.,5:cub.)
0
       1        P     3.817     15.280     15.605   111.75    93.14    92.86
       2    mcl A     3.817     25.567     17.329    88.70    95.35    90.20
       3    mcl P    15.280      3.817     15.605    93.14   111.75    92.86
       4    mcl A     3.817     25.567     17.329    91.30    84.65    90.20
       5    mcl A     3.817     25.567     17.329    88.70    84.65    89.80
       6    mcl A     3.817     25.567     17.329    91.30    95.35    89.80
*
de
 one line: # of transf. to be loaded (0: list all transf.)
           write matrix? (0:no, >0:yes)
           minim. symmetry? (0(=def.):trk.,1:mcl.,2:orh.,3:tet.,4:hex.,5:cub.)
2
                  -1.00  0.00 -0.00;   0.00  1.00 -1.00;   0.00 -1.00 -1.00;

 dir.lc.:   3.8175  25.5672  17.3289  88.70  95.35  90.20, V(A):  1683.5 trik
 rec.lc.: 0.263102 0.039123 0.057974  91.28  84.65  89.92, SG. A (***)
*
mv
 h  for help

 current cell parameters:
     3.817    25.567    17.329     88.70    95.35    90.20

  matrix 1                               matrix 2
  0.00000     0.00000     1.00000        1.00000     0.00000     0.00000
  0.00000    -1.00000     0.00000        0.00000     1.00000     0.00000
 -1.00000     0.00000     0.00000        0.00000     0.00000     1.00000
 det1: -1.000000                        det2:  1.000000

  vektor:  0.000     0.000     1.000

   v ,m1,m2,i1,i2,mi,mm,vm,mv,vv,vs,ma,mr,l ,en; h  for help
```

```
m1
 matrix 1?
0 0 1 0 -1 0 1 0 0

  matrix 1                              matrix 2
  0.00000     0.00000     1.00000        1.00000     0.00000     0.00000
  0.00000    -1.00000     0.00000        0.00000     1.00000     0.00000
  1.00000     0.00000     0.00000        0.00000     0.00000     1.00000
 det1:  1.000000                        det2:  1.000000

   v ,m1,m2,i1,i2,mi,mm,vm,mv,vv,vs,ma,mr,l ,en; h  for help
ma
 current cell parameters:
     3.817    25.567    17.329     88.70   95.35   90.20
 apply matrix 1?

 transformed cell parameters:
    17.329    25.567     3.817     89.80   95.35   91.30
  replace current cell parameters?

 dir.lc.:  17.3289  25.5672   3.8175  89.80  95.35  91.30, V(A):  1683.5 trik
 rec.lc.: 0.057974 0.039123 0.263102  90.08  84.65  88.72, SG. A (***)
 centering (col.1) (or space group)? (P,A,B,C,R,I,F)
C
 space group: C (***)
 h  for help

 current cell parameters:
    17.329    25.567     3.817     89.80   95.35   91.30

  matrix 1                              matrix 2
  0.00000     0.00000     1.00000        1.00000     0.00000     0.00000
  0.00000    -1.00000     0.00000        0.00000     1.00000     0.00000
  1.00000     0.00000     0.00000        0.00000     0.00000     1.00000
 det1:  1.000000                        det2:  1.000000

  vektor:  0.000     0.000     1.000

   v ,m1,m2,i1,i2,mi,mm,vm,mv,vv,vs,ma,mr,l ,en; h  for help
en
current cell:  17.3289  25.5672   3.8175   89.798   95.35   91.30
               check centering!
*
pg
 consec. # of data set? 0 or <0: next set (   8 )
1
-DC-
 dir.lc.:  17.3289  25.5672   3.8175  89.80  95.35  91.30, V(C):  1683.5 trik
 rec.lc.: 0.057974 0.039123 0.263102  90.08  84.65  88.72, SG. C (***)

 CuPc pattern 19
   r1: 145.00 +- 4.35; ang.:   93.28 +-  2.50;   L0:    0.00; wgt(angle): 0.6
   r2: 293.26 +- 8.80; c.c.: 1100.00 +- 55.00; L1-0:    0.00; wgt(r1/r2): 0.8
   r3: 334.50 +- 0.00; volt:     300000.     ; V(C):     0.0; wgt(c.c.) : 0.3
 mul.:    2, cent.:C, rl: 0, rewind: Y, nr:   199
*
i
       6 solution(s)
                                        L0:   0.0             300kV
   h1 k1 l1    r1  h2 k2 l2     r2  angle   L1-0    c.c.  ---- errors ----   R  mul
  obs.:    145.00            293.26  93.3    0.0 1100.0 ang. r1/r2% c.c.%
 ------------------------------------------------------------------------------
   1  3  0145.139  1 -1 -1293.121   94.1 2037.9 1099.0 -0.8   0.14 -0.09  0.62
                                                    [     -3      1     -4]
  -1  3  0144.736  0  0  1293.524   92.5 2099.8 1115.6  0.8 -0.27  1.42  1.14
                                                    [      3      1      0]
   1  3  0146.468  0  0 -1291.792   92.3 2068.8 1109.0  1.0  1.51  0.82  2.06
                                                    [     -3      1      0]
   1 -3  0143.239  1  1 -1295.021   94.6 2064.5 1104.1 -1.4 -1.82  0.37  2.38
                                                    [      3      1      4]
```

```
   2 -2  0146.954  0  2  1291.306  94.6 2683.2 1061.7 -1.3  2.01 -3.48  3.44  2
(  1 -1  0         1  1  1;   2,  0;  -1,  1)      [    -1    -1     2]
   2  2  0148.917  0 -2  1289.343  95.2 2635.7 1053.7 -1.9  4.01 -4.21  5.62  2
(  1  1  0         1 -1  1;   2,  0;  -1,  1)      [     1    -1    -2]
*
pg
 consec. # of data set? 0 or <0: next set (   2 )
2
-DC-
 dir.lc.:  17.3289  25.5672   3.8175  89.80  95.35  91.30, V(C):  1683.5 trik
 rec.lc.: 0.057974 0.039123 0.263102  90.08  84.65  88.72, SG. C (***)

 CuPc pattern 20
   r1: 144.89 +- 4.35; ang.:   74.53 +-  2.50;   L0:    0.00; wgt(angle): 0.6
   r2: 310.27 +- 9.31; c.c.: 1100.00 +- 55.00; L1-0:    0.00; wgt(r1/r2): 0.8
   r3: 305.42 +- 0.00; volt:     300000.     ; V(C):     0.0; wgt(c.c.) : 0.3
 mul.:    2, cent.:C, rl: 0, rewind: Y, nr:   199
*
i
       4 solution(s)
                                       L0:   0.0             300kV
   h1 k1 l1   r1  h2 k2 l2    r2  angle   L1-0   c.c.  ---- errors ----  R  mul
  obs.:    144.89          310.27  74.5    0.0 1100.0 ang. r1/r2% c.c.%
 ------------------------------------------------------------------------------
   1 -3  0145.079  1 -1  1310.081  75.2 2084.4 1118.3 -0.6  0.19  1.66  1.03
                                                   [    -3    -1     2]
   1  3  0146.752  1  1  1308.408  74.9 2053.0 1111.2 -0.4  1.88  1.02  2.04
                                                   [     3    -1    -2]
   1 -3  0146.044  0 -2  1309.116  72.8 2121.1 1125.7  1.8  1.17  2.34  2.69
                                                   [    -3    -1    -2]
   1  3  0147.893  0  2  1307.267  72.9 2091.5 1119.8  1.6  3.02  1.80  3.95
                                                   [     3    -1     2]
*
pg
 consec. # of data set? 0 or <0: next set (   3 )
3
-DC-
 dir.lc.:  17.3289  25.5672   3.8175  89.80  95.35  91.30, V(C):  1683.5 trik
 rec.lc.: 0.057974 0.039123 0.263102  90.08  84.65  88.72, SG. C (***)

 CuPc pattern 24
   r1: 129.29 +- 3.88; ang.:   95.58 +-  2.50;   L0:    0.00; wgt(angle): 0.6
   r2: 419.22 +-12.58; c.c.: 1100.00 +- 55.00; L1-0:    0.00; wgt(r1/r2): 0.8
   r3: 450.56 +- 0.00; volt:     300000.     ; V(C):     0.0; wgt(c.c.) : 0.3
 mul.:    2, cent.:C, rl: 0, rewind: Y, nr:   199
*
i
       2 solution(s)
                                       L0:   0.0             300kV
   h1 k1 l1   r1  h2 k2 l2    r2  angle   L1-0   c.c.  ---- errors ----  R  mul
  obs.:    129.29          419.22  95.6    0.0 1100.0 ang. r1/r2% c.c.%
 ------------------------------------------------------------------------------
   2  0  0127.813 -1 -7  1420.697  96.0 1826.1 1102.3 -0.4 -1.50  0.21  1.49
                                                   [     0    -1    -7]
   2  0  0128.434 -1  7  1420.076  94.1 1838.2 1107.7  1.5 -0.87  0.70  1.78
                                                   [     0    -1     7]
*
pg
 consec. # of data set? 0 or <0: next set (   4 )
4
-DC-
 dir.lc.:  17.3289  25.5672   3.8175  89.80  95.35  91.30, V(C):  1683.5 trik
 rec.lc.: 0.057974 0.039123 0.263102  90.08  84.65  88.72, SG. C (***)

 CuPc pattern 29
   r1:  86.21 +- 2.59; ang.:   89.37 +-  2.50;   L0:    0.00; wgt(angle): 0.6
   r2: 370.60 +-11.12; c.c.: 1100.00 +- 55.00; L1-0:    0.00; wgt(r1/r2): 0.8
   r3: 379.57 +- 0.00; volt:     300000.     ; V(C):     0.0; wgt(c.c.) : 0.3
 mul.:    2, cent.:C, rl: 0, rewind: Y, nr:   199
*
```

```
i
       1 solution(s)
                                     L0:   0.0            300kV
   h1 k1 l1   r1  h2 k2 l2    r2  angle   L1-0   c.c.  ---- errors ----  R  mul
  obs.:     86.21          370.60  89.4    0.0 1100.0 ang. r1/r2% c.c.%
 -------------------------------------------------------------------------------
   0  2  0 86.684  4  0 -1370.126  89.0 2382.1 1107.8  0.3  0.68  0.71  0.95
                                               [    -1     0    -4]
*
pg
 consec. # of data set? 0 or <0: next set (   5 )
5
-DC-
 dir.lc.:  17.3289  25.5672   3.8175  89.80  95.35  91.30, V(C):  1683.5 trik
 rec.lc.: 0.057974 0.039123 0.263102  90.08  84.65  88.72, SG. C (***)

 CuPc pattern 30
   r1:  86.26 +- 2.59; ang.:   96.54 +-  2.50;   L0:    0.00; wgt(angle): 0.6
   r2: 414.92 +-12.45; c.c.: 1100.00 +- 55.00; L1-0:    0.00; wgt(r1/r2): 0.8
   r3: 433.30 +- 0.00; volt:     300000.     ; V(C):     0.0; wgt(c.c.) : 0.3
 mul.:    2, cent.:C, rl: 0, rewind: Y, nr:   199
*
i
       2 solution(s)
                                     L0:   0.0            300kV
   h1 k1 l1   r1  h2 k2 l2    r2  angle   L1-0   c.c.  ---- errors ----  R  mul
  obs.:     86.26          414.92  96.5    0.0 1100.0 ang. r1/r2% c.c.%
 -------------------------------------------------------------------------------
   0  2  0 86.402 -5 -1  1414.778  97.0 2247.5 1104.2 -0.5  0.20  0.39  0.57
                                               [     1     0     5]
   0 -2  0 86.676 -5  1  1414.504  94.9 2254.6 1107.7  1.6  0.58  0.70  1.63
                                               [    -1     0    -5]
*
pg
 consec. # of data set? 0 or <0: next set (   6 )
6
-DC-
 dir.lc.:  17.3289  25.5672   3.8175  89.80  95.35  91.30, V(C):  1683.5 trik
 rec.lc.: 0.057974 0.039123 0.263102  90.08  84.65  88.72, SG. C (***)

 CuPc pattern 31
   r1:  86.26 +- 2.59; ang.:   85.91 +-  2.50;   L0:    0.00; wgt(angle): 0.6
   r2: 511.55 +-15.35; c.c.: 1100.00 +- 55.00; L1-0:    0.00; wgt(r1/r2): 0.8
   r3: 512.67 +- 0.00; volt:     300000.     ; V(C):     0.0; wgt(c.c.) : 0.3
 mul.:    2, cent.:C, rl: 0, rewind: Y, nr:   199
*
i
       2 solution(s)
                                     L0:   0.0            300kV
   h1 k1 l1   r1  h2 k2 l2    r2  angle   L1-0   c.c.  ---- errors ----  R  mul
  obs.:     86.26          511.55  85.9    0.0 1100.0 ang. r1/r2% c.c.%
 -------------------------------------------------------------------------------
   0  2  0 86.350 -7  1  1511.460  86.3 2016.4 1103.6 -0.4  0.12  0.33  0.45
                                               [     1     0     7]
   0  2  0 86.097  7  1 -1511.713  84.0 2010.4 1100.3  1.9 -0.22  0.03  1.33
                                               [    -1     0    -7]
*
pg
 consec. # of data set? 0 or <0: next set (   2 )
7
-DC-
 dir.lc.:  17.3289  25.5672   3.8175  89.80  95.35  91.30, V(C):  1683.5 trik
 rec.lc.: 0.057974 0.039123 0.263102  90.08  84.65  88.72, SG. C (***)

 CuPc pattern 32
   r1:  77.73 +- 2.33; ang.:   68.02 +-  2.50;   L0:    0.00; wgt(angle): 0.6
   r2:  76.13 +- 2.28; c.c.: 1100.00 +- 55.00; L1-0:    0.00; wgt(r1/r2): 0.8
   r3:  86.07 +- 0.00; volt:     300000.     ; V(C):     0.0; wgt(c.c.) : 0.3
 mul.:    2, cent.:C, rl: 0, rewind: Y, nr:   199
*
```

```
i
      2 solution(s)
                                      L0:   0.0            300kV
   h1  k1  l1    r1   h2  k2  l2     r2   angle   L1-0    c.c.   ---- errors ----   R   mul
  obs.:      77.73           76.13   68.0     0.0 1100.0 ang. r1/r2% c.c.%
 ---------------------------------------------------------------------------------------
   1   1   0 77.730   1  -1   0 76.130   68.0 5696.6 1100.0 -0.0 -0.00   0.00   0.00
                                                    [      0      0     -1]
   1  -1   0 76.130   1   1   0 77.730   68.0 5696.6 1100.0 -0.0 -4.16   0.00   3.33
                                                    [      0      0      1]
*
```

S3.3. Exchanging the pattern number 7 by the pattern number 8

```
                 #################################
                 =======      P I E P      =======
                 ======= VERSION 14-jun-17 =======
                 #################################

 default parameters from file? (def.=yes)

 parameter-file piep.par            ? (blank), otherwise name

 -------------------------------------------------------------------------
  cell parameter file assigned: cell.dat            ,    61 sets
  1st set read, unit: 20, file: cell.dat
 -------------------------------------------------------------------------
  SAD data file: unit 30, file: sad.dat             ,     8 sets
  1st set loaded
 -------------------------------------------------------------------------

ort
 dir.lc.:   5.2060  19.0120  26.8850   90.00   90.00   90.00, V(P):  2661.0 orth
 rec.lc.: 0.192086 0.052598 0.037195   90.00   90.00   90.00, SG. P (***)

 CuPc pattern 19
   r1: 145.00 +- 4.35; ang.:   93.28 +-  2.50;   L0:    0.00; wgt(angle): 0.6
   r2: 293.26 +- 8.80; c.c.: 1100.00 +- 55.00; L1-0:    0.00; wgt(r1/r2): 0.8
   r3: 334.50 +- 0.00; volt:     300000.     ; V(P):     0.0; wgt(c.c.) : 0.3
 mul.:    2, cent.:P, rl: 0, rewind: Y, nr:   199
*
ap
  free:      1  2  3  4  5  6  7  8  9 10 11 12 13 14 15 16 17 18 19 20
 which number?
1
  #        d1   s/d(%)    d2  s/d(%)   d3     ang. sig.     V      d-m   seq.
  1       7.5862 3.00   3.7509 3.00   3.2885  93.3  2.5      0.0   5.339  1
*
pg
 consec. # of data set? 0 or <0: next set (   2 )
2
ort
 dir.lc.:   5.2060  19.0120  26.8850   90.00   90.00   90.00, V(P):  2661.0 orth
 rec.lc.: 0.192086 0.052598 0.037195   90.00   90.00   90.00, SG. P (***)

 CuPc pattern 20
   r1: 144.89 +- 4.35; ang.:   74.53 +-  2.50;   L0:    0.00; wgt(angle): 0.6
   r2: 310.27 +- 9.31; c.c.: 1100.00 +- 55.00; L1-0:    0.00; wgt(r1/r2): 0.8
   r3: 305.42 +- 0.00; volt:     300000.     ; V(P):     0.0; wgt(c.c.) : 0.3
 mul.:    2, cent.:P, rl: 0, rewind: Y, nr:   199
*
ap
  free:      2  3  4  5  6  7  8  9 10 11 12 13 14 15 16 17 18 19 20
 which number?
2
  #        d1   s/d(%)    d2  s/d(%)   d3     ang. sig.     V      d-m   seq.
  1       7.5862 3.00   3.7509 3.00   3.2885  93.3  2.5      0.0   5.339  1
```

```
  2        7.5920 3.00   3.5453 3.00   3.6017  74.5  2.5      0.0  5.285  2
*
pg
 consec. # of data set? 0 or <0: next set (   3 )
3
ort
 dir.lc.:   5.2060  19.0120  26.8850  90.00  90.00  90.00, V(P):  2661.0 orth
 rec.lc.: 0.192086 0.052598 0.037195  90.00  90.00  90.00, SG. P (***)

 CuPc pattern 24
   r1: 129.29 +- 3.88; ang.:   95.58 +-  2.50;   L0:    0.00; wgt(angle): 0.6
   r2: 419.22 +-12.58; c.c.: 1100.00 +- 55.00; L1-0:    0.00; wgt(r1/r2): 0.8
   r3: 450.56 +- 0.00; volt:     300000.     ; V(P):     0.0; wgt(c.c.) : 0.3
 mul.:    2, cent.:P, rl: 0, rewind: Y, nr:   199
*
ap
  free:      3  4  5  6  7  8  9 10 11 12 13 14 15 16 17 18 19 20
 which number?
3
  #         d1   s/d(%)    d2  s/d(%)   d3     ang. sig.     V      d-m  seq.
  1        7.5862 3.00   3.7509 3.00   3.2885  93.3  2.5      0.0  5.339  1
  2        7.5920 3.00   3.5453 3.00   3.6017  74.5  2.5      0.0  5.285  2
  3        8.5080 3.00   2.6239 3.00   2.4414  95.6  2.5      0.0  4.736  3
*
pg
 consec. # of data set? 0 or <0: next set (   4 )
4
ort
 dir.lc.:   5.2060  19.0120  26.8850  90.00  90.00  90.00, V(P):  2661.0 orth
 rec.lc.: 0.192086 0.052598 0.037195  90.00  90.00  90.00, SG. P (***)

 CuPc pattern 29
   r1:  86.21 +- 2.59; ang.:   89.37 +-  2.50;   L0:    0.00; wgt(angle): 0.6
   r2: 370.60 +-11.12; c.c.: 1100.00 +- 55.00; L1-0:    0.00; wgt(r1/r2): 0.8
   r3: 379.57 +- 0.00; volt:     300000.     ; V(P):     0.0; wgt(c.c.) : 0.3
 mul.:    2, cent.:P, rl: 0, rewind: Y, nr:   199
*
ap
  free:      4  5  6  7  8  9 10 11 12 13 14 15 16 17 18 19 20
 which number?
4
  #         d1   s/d(%)    d2  s/d(%)   d3     ang. sig.     V      d-m  seq.
  1        7.5862 3.00   3.7509 3.00   3.2885  93.3  2.5      0.0  5.339  2
  2        7.5920 3.00   3.5453 3.00   3.6017  74.5  2.5      0.0  5.285  3
  3        8.5080 3.00   2.6239 3.00   2.4414  95.6  2.5      0.0  4.736  4
  4       12.7595 3.00   2.9682 3.00   2.8980  89.4  2.5      0.0  6.154  1
*
pg
 consec. # of data set? 0 or <0: next set (   5 )
5
ort
 dir.lc.:   5.2060  19.0120  26.8850  90.00  90.00  90.00, V(P):  2661.0 orth
 rec.lc.: 0.192086 0.052598 0.037195  90.00  90.00  90.00, SG. P (***)

 CuPc pattern 30
   r1:  86.26 +- 2.59; ang.:   96.54 +-  2.50;   L0:    0.00; wgt(angle): 0.6
   r2: 414.92 +-12.45; c.c.: 1100.00 +- 55.00; L1-0:    0.00; wgt(r1/r2): 0.8
   r3: 433.30 +- 0.00; volt:     300000.     ; V(P):     0.0; wgt(c.c.) : 0.3
 mul.:    2, cent.:P, rl: 0, rewind: Y, nr:   199
*
ap
  free:      5  6  7  8  9 10 11 12 13 14 15 16 17 18 19 20
 which number?
5
  #         d1   s/d(%)    d2  s/d(%)   d3     ang. sig.     V      d-m  seq.
  1        7.5862 3.00   3.7509 3.00   3.2885  93.3  2.5      0.0  5.339  3
  2        7.5920 3.00   3.5453 3.00   3.6017  74.5  2.5      0.0  5.285  4
  3        8.5080 3.00   2.6239 3.00   2.4414  95.6  2.5      0.0  4.736  5
  4       12.7595 3.00   2.9682 3.00   2.8980  89.4  2.5      0.0  6.154  1
  5       12.7521 3.00   2.6511 3.00   2.5386  96.5  2.5      0.0  5.833  2
```

```
*
pg
 consec. # of data set? 0 or <0: next set (    6 )
6
ort
 dir.lc.:    5.2060  19.0120  26.8850   90.00   90.00   90.00, V(P):  2661.0 orth
 rec.lc.: 0.192086 0.052598 0.037195   90.00   90.00   90.00, SG. P (***)

 CuPc pattern 31
   r1:  86.26 +- 2.59; ang.:   85.91 +-  2.50;   L0:    0.00; wgt(angle): 0.6
   r2: 511.55 +-15.35; c.c.: 1100.00 +- 55.00; L1-0:    0.00; wgt(r1/r2): 0.8
   r3: 512.67 +- 0.00; volt:     300000.     ; V(P):      0.0; wgt(c.c.) : 0.3
 mul.:    2, cent.:P, rl: 0, rewind: Y, nr:   199
*
ap
  free:      6  7  8  9 10 11 12 13 14 15 16 17 18 19 20
 which number?
6
  #        d1   s/d(%)    d2  s/d(%)    d3     ang. sig.      V      d-m  seq.
  1        7.5862 3.00   3.7509 3.00   3.2885  93.3  2.5      0.0  5.339  3
  2        7.5920 3.00   3.5453 3.00   3.6017  74.5  2.5      0.0  5.285  4
  3        8.5080 3.00   2.6239 3.00   2.4414  95.6  2.5      0.0  4.736  6
  4       12.7595 3.00   2.9682 3.00   2.8980  89.4  2.5      0.0  6.154  1
  5       12.7521 3.00   2.6511 3.00   2.5386  96.5  2.5      0.0  5.833  2
  6       12.7521 3.00   2.1503 3.00   2.1456  85.9  2.5      0.0  5.243  5
*
pg
 consec. # of data set? 0 or <0: next set (    7 )
8
ort
 dir.lc.:    5.2060  19.0120  26.8850   90.00   90.00   90.00, V(P):  2661.0 orth
 rec.lc.: 0.192086 0.052598 0.037195   90.00   90.00   90.00, SG. P (***)

 CuPc pattern 32i
   r1:  76.93 +- 2.31; ang.:   68.02 +-  2.50;   L0:    0.00; wgt(angle): 0.6
   r2:  76.93 +- 2.31; c.c.: 1100.00 +- 55.00; L1-0:    0.00; wgt(r1/r2): 0.8
   r3:  86.06 +- 0.00; volt:     300000.     ; V(P):      0.0; wgt(c.c.) : 0.3
 mul.:    2, cent.:P, rl: 0, rewind: Y, nr:   199
*
ap
  free:      7  8  9 10 11 12 13 14 15 16 17 18 19 20
 which number?
7
  #        d1   s/d(%)    d2  s/d(%)    d3     ang. sig.      V      d-m  seq.
  1        7.5862 3.00   3.7509 3.00   3.2885  93.3  2.5      0.0  5.339  4
  2        7.5920 3.00   3.5453 3.00   3.6017  74.5  2.5      0.0  5.285  5
  3        8.5080 3.00   2.6239 3.00   2.4414  95.6  2.5      0.0  4.736  7
  4       12.7595 3.00   2.9682 3.00   2.8980  89.4  2.5      0.0  6.154  2
  5       12.7521 3.00   2.6511 3.00   2.5386  96.5  2.5      0.0  5.833  3
  6       12.7521 3.00   2.1503 3.00   2.1456  85.9  2.5      0.0  5.243  6
  7 <     14.2987 3.00  14.2987 3.00  12.7818  68.0  2.5      0.0 14.849  1
*
pc
  #        d1   s/d(%)    d2  s/d(%)    d3     ang. sig.      V      d-m  seq.
  1        7.5862 3.00   3.7509 3.00   3.2885  93.3  2.5      0.0  5.339  3
  2        7.5920 3.00   3.5453 3.00   3.6017  74.5  2.5      0.0  5.285  4
  3        8.5080 3.00   2.6239 3.00   2.4414  95.6  2.5      0.0  4.736  6
  4       12.7595 3.00   2.9682 3.00   2.8980  89.4  2.5      0.0  6.154  1
  5       12.7521 3.00   2.6511 3.00   2.5386  96.5  2.5      0.0  5.833  2
  6       12.7521 3.00   2.1503 3.00   2.1456  85.9  2.5      0.0  5.243  5
  7 <     14.2987 3.00  14.2987 3.00  12.7818  68.0  2.5      0.0 14.849  0

 a*,b*-defining:  7;<
       sequence:       4;    5;    1;    2;    6;    3;

   1st: new a*,b* defining number (0: no changes)
   2nd: <0: enforce full grid, >0: * sigma (2.31mm) = "wall thickness"


 V(P)(min), V(P)(max)? (calc.: min:      0. max:      0., mean:      0.)
```

```
 def.:     0.     0.
0 1000
    763.  1000.
 factor for default increment (0.025), def.:1., max:6; <0 : increment

    0.025

       197 sets within  12 layers, p:  0.233 -  0.305
*
dc
  mult.<mu>: 2; <rl>: 0;  1st layer: V: 762.8; n:   14; p:  0.233
  ok?


        3 solutions stored,  R :     1.08 -   2.53, incl. equiv.:        32
       R      a     b     c     al     be    ga      x      y      V    int.
   1  1.08  3.82 15.44 15.44  111.8  92.7  92.7  0.000  0.375   841.7  1.00
   2  1.61  4.01 15.42 15.42  111.9  91.5  91.5  0.000  0.200   884.2  0.41
   3  2.53  4.32 15.43 15.43  111.9  91.6  91.6  0.000  0.200   952.0  0.08
*

de
 one line: # of transf. to be loaded (0: list all transf.)
           write matrix? (0:no, >0:yes)
           minim. symmetry? (0(=def.):trk.,1:mcl.,2:orh.,3:tet.,4:hex.,5:cub.)
0
       1       P     3.818    15.436    15.436   111.80   92.66   92.66
       2   mcl A     3.818    25.564    17.309    90.00   94.74   90.00
       3   mcl P    15.436     3.818    15.436    92.66  111.80   92.66
       4   mcl A     3.818    25.564    17.309    90.00   85.26   90.00
*
de
 one line: # of transf. to be loaded (0: list all transf.)
           write matrix? (0:no, >0:yes)
           minim. symmetry? (0(=def.):trk.,1:mcl.,2:orh.,3:tet.,4:hex.,5:cub.)
2
                  -1.00  0.00 -0.00;   0.00  1.00 -1.00;   0.00 -1.00 -1.00;

 dir.lc.:   3.8176  25.5637  17.3087  90.00  94.74  90.00, V(A):  1683.4 mcl.
 rec.lc.: 0.262844 0.039118 0.057973  90.00  85.26  90.00, SG. A (***)
*
mv
 h  for help

 current cell parameters:
     3.818    25.564    17.309     90.00   94.74   90.00

  matrix 1                               matrix 2
  1.00000     0.00000     0.00000        1.00000     0.00000     0.00000
  0.00000     1.00000     0.00000        0.00000     1.00000     0.00000
  0.00000     0.00000     1.00000        0.00000     0.00000     1.00000
 det1:  1.000000                        det2:  1.000000

  vektor:  1.000     0.000     0.000

   v ,m1,m2,i1,i2,mi,mm,vm,mv,vv,vs,ma,mr,l ,en; h  for help
m1
 matrix 1?
0 0 1 0 -1 0 1 0 0

  matrix 1                               matrix 2
  0.00000     0.00000     1.00000        1.00000     0.00000     0.00000
  0.00000    -1.00000     0.00000        0.00000     1.00000     0.00000
  1.00000     0.00000     0.00000        0.00000     0.00000     1.00000
 det1:  1.000000                        det2:  1.000000

   v ,m1,m2,i1,i2,mi,mm,vm,mv,vv,vs,ma,mr,l ,en; h  for help
ma
 current cell parameters:
     3.818    25.564    17.309     90.00   94.74   90.00
```

```
 apply matrix 1?

 transformed cell parameters:
    17.309    25.564     3.818     90.00   94.74   90.00
  replace current cell parameters?

 dir.lc.:  17.3087  25.5637   3.8176  90.00  94.74  90.00, V(A):  1683.4 mcl.
 rec.lc.: 0.057973 0.039118 0.262844  90.00  85.26  90.00, SG. A (***)
 centering (col.1) (or space group)? (P,A,B,C,R,I,F)
C
 space group: C (***)
 h  for help

 current cell parameters:
    17.309    25.564     3.818     90.00   94.74   90.00

  matrix 1                              matrix 2
  0.00000     0.00000     1.00000        1.00000     0.00000     0.00000
  0.00000    -1.00000     0.00000        0.00000     1.00000     0.00000
  1.00000     0.00000     0.00000        0.00000     0.00000     1.00000
 det1:  1.000000                        det2:  1.000000

  vektor:  1.000     0.000     0.000

   v ,m1,m2,i1,i2,mi,mm,vm,mv,vv,vs,ma,mr,l ,en; h  for help
en
current cell:  17.3087  25.5637   3.8176   90.000   94.74   90.00
               check centering!
*
```

S4. PIEP run for unit cell parameters determination for lysozyme

S4.1. Input file (sad.dat) for lysozyme

```
  1 P.2.0434 [001]
  719.420   35.971   9.0992   0.2730   9.0992   0.2730
  12.8682   0.0000  90.0000   2.5000     0.00     0.00     0.00
     0.00     5.00     0.00     5.00  200000.
  2 P.2.0038
  719.420   35.971   9.2851   0.2786 111.3000   3.3390
 111.6866   0.0000  90.0000   2.5000     0.00     0.00     0.00
     0.00     5.00     0.00     5.00  200000.
  3 P.2.0063
  719.420   35.971   9.1075   0.2732  49.0000   1.4700
  49.8392   0.0000  90.0000   2.5000     0.00     0.00     0.00
     0.00     5.00     0.00     5.00  200000.
  4 P.2.1152
  719.420   35.971   9.3286   0.2799  75.5000   2.2650
  76.0741   0.0000  90.0000   2.5000     0.00     0.00     0.00
     0.00     5.00     0.00     5.00  200000.
  5 P.2.0836
  719.420   35.971   9.1996   0.2760  84.4000   2.5320
  84.8999   0.0000  90.0000   2.5000     0.00     0.00     0.00
     0.00     5.00     0.00     5.00  200000.
  6 P.2.0974
  719.420   35.971   9.0334   0.2710  59.5000   1.7850
  60.1818   0.0000  90.0000   2.5000     0.00     0.00     0.00
     0.00     5.00     0.00     5.00  200000.
  7 P.2.1064
  719.420   35.971  12.9200   0.3876  80.1000   2.4030
  81.1353   0.0000  90.0000   2.5000     0.00     0.00     0.00
     0.00     5.00     0.00     5.00  200000.
  8 P.2.0007
  719.420   35.971  13.3156   0.3995  56.4000   1.6921
  57.8826   0.0000  89.7000   2.5000     0.00     0.00     0.00
     0.00     5.00     0.00     5.00  200000.
  9 P.2.0028
```

```
  719.420   35.971  12.9533   0.3886  20.8768   0.6263
  21.1133   0.0000  73.0300   2.5000     0.00     0.00     0.00
     0.00     5.00     0.00     5.00  200000.
  10 P.2.0103
  719.420   35.971  28.4829   0.8545  42.4902   1.2747
  49.6215   0.0000  86.3427   2.5000     0.00     0.00     0.00
     0.00     5.00     0.00     5.00  200000.
  11 P.2.0345
  719.420   35.971  28.7452   0.8624  33.1950   0.9959
  37.4685   0.0000  74.0536   2.5000     0.00     0.00     0.00
     0.00     5.00     0.00     5.00  200000.
  12 P.2.0379
  719.420   35.971  23.1055   0.6932  27.8886   0.8367
  33.0743   0.0000  80.2735   2.5000     0.00     0.00     0.00
     0.00     5.00     0.00     5.00  200000.
  13 P.2.0610
  719.420   35.971  12.8534   0.3856  86.9843   2.6095
  87.2000   0.0000  86.7276   2.5000     0.00     0.00     0.00
     0.00     5.00     0.00     5.00  200000.
  14 P.2.0753
  719.420   35.971  19.0276   0.5708  46.5243   1.3957
  49.8649   0.0000  88.7038   2.5000     0.00     0.00     0.00
     0.00     5.00     0.00     5.00  200000.
  15 P.2.0806
  719.420   35.971  31.5591   0.9468  50.5429   1.5163
  57.4893   0.0000  85.5857   2.5000     0.00     0.00     0.00
     0.00     5.00     0.00     5.00  200000.
  16 P.2.0872
  719.420   35.971  22.3492   0.6705  38.8657   1.1660
  42.1672   0.0000  82.3268   2.5000     0.00     0.00     0.00
     0.00     5.00     0.00     5.00  200000.
  17 P.2.1099
  719.420   35.971  13.0585   0.3918  38.0952   1.1429
  38.6243   0.0000  82.4963   2.5000     0.00     0.00     0.00
     0.00     5.00     0.00     5.00  200000.
  18 P.2.1149
  719.420   35.971  12.7300   0.3819 101.5000   3.0450
 101.6940   0.0000  87.2800   2.5000     0.00     0.00     0.00
     0.00     5.00     0.00     5.00  200000.
END$
```

S4.2. Communication protocol with PIEP

```
                ##################################
                =======      P I E P      =======
                ======= VERSION 14-jun-17 =======
                ##################################

 default parameters from file? (def.=yes)

 parameter-file piep.par            ? (blank), otherwise name

 -----------------------------------------------------------------------
  cell parameter file assigned: cell.dat            ,    21 sets
  1st set read, unit: 20, file: cell.dat
 -----------------------------------------------------------------------
  SAD data file: unit 30, file: sad.dat             ,    18 sets
  1st set loaded
 -----------------------------------------------------------------------

dummy
 dir.lc.:  10.0000  10.0000  10.0000  90.00  90.00  90.00, V(F):  1000.0 cub.
 rec.lc.: 0.100000 0.100000 0.100000  90.00  90.00  90.00, SG. F (***)

P.2.0434 [001]
   r1:   9.10 +- 0.27; ang.:   90.00 +-  2.50;   L0:    0.00; wgt(angle): 0.6
   r2:   9.10 +- 0.27; c.c.:  719.42 +- 35.97; L1-0:    0.00; wgt(r1/r2): 0.8
```

```
   r3:  12.87 +- 0.00; volt:     200000.     ; V(F):     0.0; wgt(c.c.) : 0.3
 mul.:    2, cent.:F, rl: 0, rewind: Y, nr:   199
*
ap
  free:       1  2  3  4  5  6  7  8  9 10 11 12 13 14 15 16 17 18 19 20
 which number?
1
  #         d1   s/d(%)    d2  s/d(%)   d3     ang. sig.     V      d-m  seq.
  1 4     79.0641 3.00  79.0641 3.00  55.9068  90.0  2.5      0.0 79.064  1
*
pg
 consec. # of data set? 0 or <0: next set (   2 )

dummy
 dir.lc.:  10.0000  10.0000  10.0000  90.00  90.00  90.00, V(F):  1000.0 cub.
 rec.lc.: 0.100000 0.100000 0.100000  90.00  90.00  90.00, SG. F (***)

P.2.0038
   r1:   9.29 +- 0.28; ang.:   90.00 +-  2.50;   L0:    0.00; wgt(angle): 0.6
   r2: 111.30 +- 3.34; c.c.:  719.42 +- 35.97; L1-0:    0.00; wgt(r1/r2): 0.8
   r3: 111.69 +- 0.00; volt:     200000.     ; V(F):     0.0; wgt(c.c.) : 0.3
 mul.:    2, cent.:F, rl: 0, rewind: Y, nr:   199
*
ap
  free:       2  3  4  5  6  7  8  9 10 11 12 13 14 15 16 17 18 19 20
 which number?
2
  #         d1   s/d(%)    d2  s/d(%)   d3     ang. sig.     V      d-m  seq.
  1 4     79.0641 3.00  79.0641 3.00  55.9068  90.0  2.5      0.0 79.064  1
  2 L     77.4811 3.00   6.4638 3.00   6.4414  90.0  2.5      0.0 22.379  2
*
pg
 consec. # of data set? 0 or <0: next set (   3 )

dummy
 dir.lc.:  10.0000  10.0000  10.0000  90.00  90.00  90.00, V(F):  1000.0 cub.
 rec.lc.: 0.100000 0.100000 0.100000  90.00  90.00  90.00, SG. F (***)

P.2.0063
   r1:   9.11 +- 0.27; ang.:   90.00 +-  2.50;   L0:    0.00; wgt(angle): 0.6
   r2:  49.00 +- 1.47; c.c.:  719.42 +- 35.97; L1-0:    0.00; wgt(r1/r2): 0.8
   r3:  49.84 +- 0.00; volt:     200000.     ; V(F):     0.0; wgt(c.c.) : 0.3
 mul.:    2, cent.:F, rl: 0, rewind: Y, nr:   199
*
ap
  free:       3  4  5  6  7  8  9 10 11 12 13 14 15 16 17 18 19 20
 which number?
3
  #         d1   s/d(%)    d2  s/d(%)   d3     ang. sig.     V      d-m  seq.
  1 4     79.0641 3.00  79.0641 3.00  55.9068  90.0  2.5      0.0 79.064  1
  2 L     77.4811 3.00   6.4638 3.00   6.4414  90.0  2.5      0.0 22.379  3
  3 L     78.9920 3.00  14.6820 3.00  14.4348  90.0  2.5      0.0 34.055  2
*
pg
 consec. # of data set? 0 or <0: next set (   4 )

dummy
 dir.lc.:  10.0000  10.0000  10.0000  90.00  90.00  90.00, V(F):  1000.0 cub.
 rec.lc.: 0.100000 0.100000 0.100000  90.00  90.00  90.00, SG. F (***)

P.2.1152
   r1:   9.33 +- 0.28; ang.:   90.00 +-  2.50;   L0:    0.00; wgt(angle): 0.6
   r2:  75.50 +- 2.27; c.c.:  719.42 +- 35.97; L1-0:    0.00; wgt(r1/r2): 0.8
   r3:  76.07 +- 0.00; volt:     200000.     ; V(F):     0.0; wgt(c.c.) : 0.3
 mul.:    2, cent.:F, rl: 0, rewind: Y, nr:   199
*
ap
  free:       4  5  6  7  8  9 10 11 12 13 14 15 16 17 18 19 20
 which number?
4
```

```
  #         d1   s/d(%)    d2  s/d(%)   d3      ang. sig.     V      d-m   seq.
  1 4     79.0641 3.00  79.0641 3.00  55.9068  90.0  2.5      0.0 79.064  1
  2 L     77.4811 3.00   6.4638 3.00   6.4414  90.0  2.5      0.0 22.379  4
  3 L     78.9920 3.00  14.6820 3.00  14.4348  90.0  2.5      0.0 34.055  2
  4 L     77.1198 3.00   9.5287 3.00   9.4568  90.0  2.5      0.0 27.108  3
*
pg
 consec. # of data set? 0 or <0: next set (   5 )

dummy
 dir.lc.:  10.0000  10.0000  10.0000  90.00  90.00  90.00, V(F):  1000.0 cub.
 rec.lc.: 0.100000 0.100000 0.100000  90.00  90.00  90.00, SG. F (***)

P.2.0836
   r1:   9.20 +- 0.28; ang.:   90.00 +-  2.50;   L0:    0.00; wgt(angle): 0.6
   r2:  84.40 +- 2.53; c.c.:  719.42 +- 35.97; L1-0:    0.00; wgt(r1/r2): 0.8
   r3:  84.90 +- 0.00; volt:     200000.     ; V(F):     0.0; wgt(c.c.) : 0.3
 mul.:    2, cent.:F, rl: 0, rewind: Y, nr:   199
*
ap
  free:      5   6   7   8   9 10 11 12 13 14 15 16 17 18 19 20
 which number?
5
  #         d1   s/d(%)    d2  s/d(%)   d3      ang. sig.     V      d-m   seq.
  1 4     79.0641 3.00  79.0641 3.00  55.9068  90.0  2.5      0.0 79.064  1
  2 L     77.4811 3.00   6.4638 3.00   6.4414  90.0  2.5      0.0 22.379  5
  3 L     78.9920 3.00  14.6820 3.00  14.4348  90.0  2.5      0.0 34.055  2
  4 L     77.1198 3.00   9.5287 3.00   9.4568  90.0  2.5      0.0 27.108  3
  5 L     78.2012 3.00   8.5239 3.00   8.4737  90.0  2.5      0.0 25.818  4
*
pg
 consec. # of data set? 0 or <0: next set (   6 )

dummy
 dir.lc.:  10.0000  10.0000  10.0000  90.00  90.00  90.00, V(F):  1000.0 cub.
 rec.lc.: 0.100000 0.100000 0.100000  90.00  90.00  90.00, SG. F (***)

P.2.0974
   r1:   9.03 +- 0.27; ang.:   90.00 +-  2.50;   L0:    0.00; wgt(angle): 0.6
   r2:  59.50 +- 1.78; c.c.:  719.42 +- 35.97; L1-0:    0.00; wgt(r1/r2): 0.8
   r3:  60.18 +- 0.00; volt:     200000.     ; V(F):     0.0; wgt(c.c.) : 0.3
 mul.:    2, cent.:F, rl: 0, rewind: Y, nr:   199
*
ap
  free:      6   7   8   9 10 11 12 13 14 15 16 17 18 19 20
 which number?
6
  #         d1   s/d(%)    d2  s/d(%)   d3      ang. sig.     V      d-m   seq.
  1 4     79.0641 3.00  79.0641 3.00  55.9068  90.0  2.5      0.0 79.064  1
  2 L     77.4811 3.00   6.4638 3.00   6.4414  90.0  2.5      0.0 22.379  6
  3 L     78.9920 3.00  14.6820 3.00  14.4348  90.0  2.5      0.0 34.055  2
  4 L     77.1198 3.00   9.5287 3.00   9.4568  90.0  2.5      0.0 27.108  4
  5 L     78.2012 3.00   8.5239 3.00   8.4737  90.0  2.5      0.0 25.818  5
  6 L     79.6400 3.00  12.0911 3.00  11.9541  90.0  2.5      0.0 31.031  3
*
pc
  #         d1   s/d(%)    d2  s/d(%)   d3      ang. sig.     V      d-m   seq.
  1 4     79.0641 3.00  79.0641 3.00  55.9068  90.0  2.5      0.0 79.064  0
  2 L     77.4811 3.00   6.4638 3.00   6.4414  90.0  2.5      0.0 22.379  5
  3 L     78.9920 3.00  14.6820 3.00  14.4348  90.0  2.5      0.0 34.055  1
  4 L     77.1198 3.00   9.5287 3.00   9.4568  90.0  2.5      0.0 27.108  3
  5 L     78.2012 3.00   8.5239 3.00   8.4737  90.0  2.5      0.0 25.818  4
  6 L     79.6400 3.00  12.0911 3.00  11.9541  90.0  2.5      0.0 31.031  2

 a*,b*-defining:  1;4
       sequence:       3;L  6;L  4;L  5;L  2;L

   1st: new a*,b* defining number (0: no changes)
   2nd: <0: enforce full grid, >0: * sigma (0.27mm) = "wall thickness"
```

```
             ****** warning: with pattern  1 a*,b*-defining
             ****** all cells will be tetragonal or cubic


 V(P)(min), V(P)(max)? (calc.: min:     0. max:     0., mean:     0.)
 def.:     0.     0.
0 300000
  84651.300000.
 factor for default increment (0.025), def.:1., max:6; <0 : increment

    0.025

       102 sets within  51 layers, p:  0.171 -  0.607
*
dc
  mult.<mu>: 2; <rl>: 0;  1st layer: V:******; n:    2; p:  0.171
  ok?


       11 solutions stored,  R :     0.80 -   2.24, incl. equiv.:        26
       R      a     b     c      al     be     ga      x       y       V    int.
   1  0.80 38.22 79.06 79.06   90.0  90.0  90.0  0.000  0.000238898.3  0.86
   2  0.91 28.93 79.06 79.06   90.0  90.0  90.0  0.000  0.000180852.8  0.79
   3  0.94 44.48 79.06 79.06   90.0  90.0  90.0  0.000  0.000278068.6  0.82
   4  0.99 47.99 79.06 79.06   90.0  90.0  90.0  0.000  0.000300000.0  0.41
   5  1.02 30.43 79.06 79.06   90.0  90.0  90.0  0.000  0.000190241.3  0.40
   6  1.07 35.42 81.02 81.02   87.3  77.4  77.4  0.500  0.500221433.7  1.00
   7  1.25 17.89 79.06 79.06   90.0  90.0  90.0  0.000  0.000111819.9  0.67
   8  1.49 33.67 79.06 79.06   90.0  90.0  90.0  0.000  0.000210505.8  0.62
   9  1.54 32.01 79.06 79.06   90.0  90.0  90.0  0.000  0.000200117.2  0.60
  10  2.06 28.93 80.38 80.38   88.1  79.6  79.6  0.500  0.500180852.8  0.26
  11  2.24 23.63 79.06 79.06   90.0  90.0  90.0  0.000  0.000147709.0  0.24
*
de
 one line: # of transf. to be loaded (0: list all transf.)
           write matrix? (0:no, >0:yes)
           minim. symmetry? (0(=def.):trk.,1:mcl.,2:orh.,3:tet.,4:hex.,5:cub.)
0
       1       P    38.217    79.064    79.064    90.00   90.00   90.00
       2-  tet P    79.064    79.064    38.217    90.00   90.00   90.00
       3.  orh P    38.217    79.064    79.064    90.00   90.00   90.00
       4.  orh A    38.217   111.814   111.814    90.00   90.00   90.00
       5   mcl P    38.217    79.064    79.064    90.00   90.00   90.00
       6   mcl P    79.064    38.217    79.064    90.00   90.00   90.00
       7   mcl A    38.217   111.814   111.814    90.00   90.00   90.00
*
de
 one line: # of transf. to be loaded (0: list all transf.)
           write matrix? (0:no, >0:yes)
           minim. symmetry? (0(=def.):trk.,1:mcl.,2:orh.,3:tet.,4:hex.,5:cub.)
2
                   0.00  1.00  0.00;   0.00  0.00  1.00;   1.00  0.00  0.00;

 dir.lc.:  79.0641  79.0641  38.2168  90.00  90.00  90.00, V(P):238898.3 tetr
 rec.lc.: 0.012648 0.012648 0.026166  90.00  90.00  90.00, SG. P (***)
*
pg
 consec. # of data set? 0 or <0: next set (   7 )
1
-DC-
 dir.lc.:  79.0641  79.0641  38.2168  90.00  90.00  90.00, V(P):238898.3 tetr
 rec.lc.: 0.012648 0.012648 0.026166  90.00  90.00  90.00, SG. P (***)

P.2.0434  [001]
   r1:   9.10 +- 0.27; ang.:   90.00 +-  2.50;   L0:    0.00; wgt(angle): 0.6
   r2:   9.10 +- 0.27; c.c.:  719.42 +- 35.97; L1-0:    0.00; wgt(r1/r2): 0.8
   r3:  12.87 +- 0.00; volt:     200000.     ; V(P):     0.0; wgt(c.c.) : 0.3
 mul.:    2, cent.:P, rl: 0, rewind: Y, nr:   199
*
i
```

```
       1 solution(s)
                                               L0:   0.0            200kV
   h1 k1 l1   r1  h2 k2 l2    r2  angle   L1-0    c.c.  ---- errors ----  R  mul
  obs.:      9.10            9.10  90.0    0.0  719.4 ang. r1/r2% c.c.%
 ----------------------------------------------------------------------------
   0  1  0  9.099  1  0  0  9.099  90.0 1039.8  719.4  0.0  0.00  0.00  0.00
                                                   [     0     0    -1]
*
pg
 consec. # of data set? 0 or <0: next set (   2 )

-DC-
 dir.lc.:  79.0641  79.0641  38.2168  90.00  90.00  90.00, V(P):238898.3 tetr
 rec.lc.: 0.012648 0.012648 0.026166  90.00  90.00  90.00, SG. P (***)

P.2.0038
   r1:   9.29 +- 0.28; ang.:   90.00 +-  2.50;   L0:    0.00; wgt(angle): 0.6
   r2: 111.30 +- 3.34; c.c.:  719.42 +- 35.97; L1-0:    0.00; wgt(r1/r2): 0.8
   r3: 111.69 +- 0.00; volt:     200000.     ; V(P):     0.0; wgt(c.c.) : 0.3
 mul.:    2, cent.:P, rl: 0, rewind: Y, nr:   199
*
i
       5 solution(s)
                                               L0:   0.0            200kV
   h1 k1 l1   r1  h2 k2 l2    r2  angle   L1-0    c.c.  ---- errors ----  R  mul
  obs.:      9.29          111.30  90.0    0.0  719.4 ang. r1/r2% c.c.%
 ----------------------------------------------------------------------------
   0  1  0  9.306  6  0  5111.279  90.0  307.4  735.7  0.0  0.24  2.27  0.87
                                                   [     5     0    -6]
   0  1  0  9.151 12  0  1111.434  90.0  299.5  723.5  0.0 -1.57  0.57  1.43
                                                   [     1     0   -12]
   0  1  0  9.117  9  0  4111.468  90.0  297.7  720.8  0.0 -1.98  0.20  1.64
                                                   [     4     0    -9]
   0  1  0  9.443 10  0  3111.142  90.0  314.3  746.6  0.0  1.83  3.78  2.60
                                                   [     3     0   -10]
   0  1  0  9.456 11  0  2111.129  90.0  315.1  747.6  0.0  1.98  3.92  2.76
                                                   [     2     0   -11]
*
pg
 consec. # of data set? 0 or <0: next set (   3 )

-DC-
 dir.lc.:  79.0641  79.0641  38.2168  90.00  90.00  90.00, V(P):238898.3 tetr
 rec.lc.: 0.012648 0.012648 0.026166  90.00  90.00  90.00, SG. P (***)

P.2.0063
   r1:   9.11 +- 0.27; ang.:   90.00 +-  2.50;   L0:    0.00; wgt(angle): 0.6
   r2:  49.00 +- 1.47; c.c.:  719.42 +- 35.97; L1-0:    0.00; wgt(r1/r2): 0.8
   r3:  49.84 +- 0.00; volt:     200000.     ; V(P):     0.0; wgt(c.c.) : 0.3
 mul.:    2, cent.:P, rl: 0, rewind: Y, nr:   199
*
i
       1 solution(s)
                                               L0:   0.0            200kV
   h1 k1 l1   r1  h2 k2 l2    r2  angle   L1-0    c.c.  ---- errors ----  R  mul
  obs.:      9.11           49.00  90.0    0.0  719.4 ang. r1/r2% c.c.%
 ----------------------------------------------------------------------------
   0  1  0  9.064  5  0  1 49.044  90.0  445.1  716.6  0.0 -0.57 -0.39  0.58
                                                   [     1     0    -5]
*
pg
 consec. # of data set? 0 or <0: next set (   4 )

-DC-
 dir.lc.:  79.0641  79.0641  38.2168  90.00  90.00  90.00, V(P):238898.3 tetr
 rec.lc.: 0.012648 0.012648 0.026166  90.00  90.00  90.00, SG. P (***)

P.2.1152
   r1:   9.33 +- 0.28; ang.:   90.00 +-  2.50;   L0:    0.00; wgt(angle): 0.6
   r2:  75.50 +- 2.27; c.c.:  719.42 +- 35.97; L1-0:    0.00; wgt(r1/r2): 0.8
```

```
   r3:  76.07 +- 0.00; volt:     200000.     ; V(P):     0.0; wgt(c.c.) : 0.3
 mul.:    2, cent.:P, rl: 0, rewind: Y, nr:   199
*
i
       4 solution(s)
                                       L0:   0.0             200kV
   h1 k1 l1   r1  h2 k2 l2    r2  angle   L1-0    c.c.  ---- errors ----  R  mul
  obs.:      9.33            75.50  90.0    0.0   719.4 ang. r1/r2% c.c.%
 ------------------------------------------------------------------------------
   0  1  0  9.290  7  0  2 75.539  90.0  372.1  734.5  0.0 -0.47  2.09  1.00
                                                      [     2     0    -7]
   0  1  0  9.158  8  0  1 75.671  90.0  363.8  724.0  0.0 -2.08  0.64  1.85
                                                      [     1     0    -8]
   0  1  0  9.087  1  0  4 75.742  90.0  359.5  718.4  0.0 -2.95 -0.14  2.40
                                                      [     4     0    -1]
   0  1  0  9.457  5  0  3 75.372  90.0  382.6  747.7  0.0  1.54  3.93  2.41
                                                      [     3     0    -5]
*
pg
 consec. # of data set? 0 or <0: next set (   5 )

-DC-
 dir.lc.:  79.0641  79.0641  38.2168  90.00  90.00  90.00, V(P):238898.3 tetr
 rec.lc.: 0.012648 0.012648 0.026166  90.00  90.00  90.00, SG. P (***)

P.2.0836
   r1:   9.20 +- 0.28; ang.:   90.00 +-  2.50;   L0:    0.00; wgt(angle): 0.6
   r2:  84.40 +- 2.53; c.c.:  719.42 +- 35.97; L1-0:    0.00; wgt(r1/r2): 0.8
   r3:  84.90 +- 0.00; volt:     200000.     ; V(P):     0.0; wgt(c.c.) : 0.3
 mul.:    2, cent.:P, rl: 0, rewind: Y, nr:   199
*
i
       3 solution(s)
                                       L0:   0.0             200kV
   h1 k1 l1   r1  h2 k2 l2    r2  angle   L1-0    c.c.  ---- errors ----  R  mul
  obs.:      9.20            84.40  90.0    0.0   719.4 ang. r1/r2% c.c.%
 ------------------------------------------------------------------------------
   0  1  0  9.145  9  0  1 84.454  90.0  343.7  723.1  0.0 -0.66  0.51  0.68
                                                      [     1     0    -9]
   0  1  0  9.039  7  0  3 84.561  90.0  337.6  714.6  0.0 -1.95 -0.66  1.76
                                                      [     3     0    -7]
   0  1  0  9.354  8  0  2 84.246  90.0  503.5  739.5  0.0  1.84  2.80  2.31  2
(  0  1  0         4  0  1;   1,  0;    0,  2)        [     1     0    -4]
*
pg
 consec. # of data set? 0 or <0: next set (   6 )

-DC-
 dir.lc.:  79.0641  79.0641  38.2168  90.00  90.00  90.00, V(P):238898.3 tetr
 rec.lc.: 0.012648 0.012648 0.026166  90.00  90.00  90.00, SG. P (***)

P.2.0974
   r1:   9.03 +- 0.27; ang.:   90.00 +-  2.50;   L0:    0.00; wgt(angle): 0.6
   r2:  59.50 +- 1.78; c.c.:  719.42 +- 35.97; L1-0:    0.00; wgt(r1/r2): 0.8
   r3:  60.18 +- 0.00; volt:     200000.     ; V(P):     0.0; wgt(c.c.) : 0.3
 mul.:    2, cent.:P, rl: 0, rewind: Y, nr:   199
*
i
       2 solution(s)
                                       L0:   0.0             200kV
   h1 k1 l1   r1  h2 k2 l2    r2  angle   L1-0    c.c.  ---- errors ----  R  mul
  obs.:      9.03            59.50  90.0    0.0   719.4 ang. r1/r2% c.c.%
 ------------------------------------------------------------------------------
   0  1  0  9.113  2  0  3 59.421  90.0  407.6  720.5  0.0  1.01  0.15  0.85
                                                      [     3     0    -2]
   0  1  0  9.150  5  0  2 59.383  90.0  410.2  723.4  0.0  1.48  0.56  1.35
                                                      [     2     0    -5]
*
pg
 consec. # of data set? 0 or <0: next set (   7 )
```

```
-DC-
 dir.lc.:  79.0641  79.0641  38.2168  90.00  90.00  90.00, V(P):238898.3 tetr
 rec.lc.: 0.012648 0.012648 0.026166  90.00  90.00  90.00, SG. P (***)

P.2.1064
   r1:  12.92 +- 0.39; ang.:   90.00 +-  2.50;   L0:    0.00; wgt(angle): 0.6
   r2:  80.10 +- 2.40; c.c.:  719.42 +- 35.97; L1-0:    0.00; wgt(r1/r2): 0.8
   r3:  81.14 +- 0.00; volt:     200000.     ; V(P):     0.0; wgt(c.c.) : 0.3
 mul.:    2, cent.:P, rl: 0, rewind: Y, nr:   199
*
i
       1 solution(s)
                                                     L0:   0.0            200kV
   h1 k1 l1   r1  h2 k2 l2    r2  angle   L1-0   c.c.  ---- errors ----   R  mul
  obs.:     12.92           80.10  90.0    0.0  719.4 ang. r1/r2% c.c.%
 -------------------------------------------------------------------------------
  -1  1  0 12.963  6  6  1 80.057  90.0  297.8  724.7  0.0  0.39  0.74  0.53
                                                  [     1     1   -12]
*
pg
 consec. # of data set? 0 or <0: next set (   8 )

-DC-
 dir.lc.:  79.0641  79.0641  38.2168  90.00  90.00  90.00, V(P):238898.3 tetr
 rec.lc.: 0.012648 0.012648 0.026166  90.00  90.00  90.00, SG. P (***)

P.2.0007
   r1:  13.32 +- 0.40; ang.:   89.70 +-  2.50;   L0:    0.00; wgt(angle): 0.6
   r2:  56.40 +- 1.69; c.c.:  719.42 +- 35.97; L1-0:    0.00; wgt(r1/r2): 0.8
   r3:  57.88 +- 0.00; volt:     200000.     ; V(P):     0.0; wgt(c.c.) : 0.3
 mul.:    2, cent.:P, rl: 0, rewind: Y, nr:   199
*
i
       2 solution(s)
                                                     L0:   0.0            200kV
   h1 k1 l1   r1  h2 k2 l2    r2  angle   L1-0   c.c.  ---- errors ----   R  mul
  obs.:     13.32           56.40  89.7    0.0  719.4 ang. r1/r2% c.c.%
 -------------------------------------------------------------------------------
  -1  1  0 13.256  4  4  1 56.459  90.0  366.8  741.1 -0.3 -0.55  3.01  1.53
                                                  [     1     1    -8]
  -1  1  0 13.431  3  3  2 56.284  90.0  529.9  750.9 -0.3  1.07  4.38  2.35  2
( -1  1  0         2  1  1;   1,  0;   1,  2)     [     1     1    -3]
*
pg
 consec. # of data set? 0 or <0: next set (   9 )

-DC-
 dir.lc.:  79.0641  79.0641  38.2168  90.00  90.00  90.00, V(P):238898.3 tetr
 rec.lc.: 0.012648 0.012648 0.026166  90.00  90.00  90.00, SG. P (***)

P.2.0028
   r1:  12.95 +- 0.39; ang.:   73.03 +-  2.50;   L0:    0.00; wgt(angle): 0.6
   r2:  20.88 +- 0.63; c.c.:  719.42 +- 35.97; L1-0:    0.00; wgt(r1/r2): 0.8
   r3:  21.11 +- 0.00; volt:     200000.     ; V(P):     0.0; wgt(c.c.) : 0.3
 mul.:    2, cent.:P, rl: 0, rewind: Y, nr:   199
*
i
       2 solution(s)
                                                     L0:   0.0            200kV
   h1 k1 l1   r1  h2 k2 l2    r2  angle   L1-0   c.c.  ---- errors ----   R  mul
  obs.:     12.95           20.88  73.0    0.0  719.4 ang. r1/r2% c.c.%
 -------------------------------------------------------------------------------
   1  1  0 12.889  1  0  1 20.942  72.1  592.1  720.6  1.0 -0.81  0.16  1.27
                                                  [     1    -1    -1]

                                              1 equivalent solution(s) not listed
*
pg
 consec. # of data set? 0 or <0: next set (  10 )
```

```
-DC-
 dir.lc.:  79.0641  79.0641  38.2168  90.00  90.00  90.00, V(P):238898.3 tetr
 rec.lc.: 0.012648 0.012648 0.026166  90.00  90.00  90.00, SG. P (***)

 P.2.0103
   r1:  28.48 +- 0.85; ang.:   86.34 +-  2.50;   L0:    0.00; wgt(angle): 0.6
   r2:  42.49 +- 1.27; c.c.:  719.42 +- 35.97; L1-0:    0.00; wgt(r1/r2): 0.8
   r3:  49.62 +- 0.00; volt:     200000.     ; V(P):     0.0; wgt(c.c.) : 0.3
 mul.:    2, cent.:P, rl: 0, rewind: Y, nr:   199
*
i
       6 solution(s)
                                        L0:   0.0             200kV
   h1 k1 l1   r1  h2 k2 l2    r2  angle  L1-0    c.c.  ---- errors ----  R  mul
  obs.:     28.48           42.49  86.3    0.0  719.4 ang. r1/r2% c.c.%
 ------------------------------------------------------------------------------
  -1  3  0 28.534  2  1  2 42.439  86.1  267.6  713.4  0.2  0.30 -0.83  0.61
                                               [    6     2    -7]
   1 -3  0 28.865  4  1  1 42.108  86.1  273.2  721.7  0.3  2.24  0.32  2.05
                                               [   -3    -1    13]
  -1  2  1 28.638  4  0  1 42.335  88.8  290.0  743.3 -2.5  0.91  3.31  3.21
                                               [    2     5    -8]
  -2  1  1 27.838  3  3  1 43.135  84.9  275.9  722.5  1.5 -3.79  0.43  4.03
                                               [   -2     5    -9]

                                     2 equivalent solution(s) not listed
*
pg
 consec. # of data set? 0 or <0: next set (  11 )

-DC-
 dir.lc.:  79.0641  79.0641  38.2168  90.00  90.00  90.00, V(P):238898.3 tetr
 rec.lc.: 0.012648 0.012648 0.026166  90.00  90.00  90.00, SG. P (***)

 P.2.0345
   r1:  28.75 +- 0.86; ang.:   74.05 +-  2.50;   L0:    0.00; wgt(angle): 0.6
   r2:  33.19 +- 1.00; c.c.:  719.42 +- 35.97; L1-0:    0.00; wgt(r1/r2): 0.8
   r3:  37.47 +- 0.00; volt:     200000.     ; V(P):     0.0; wgt(c.c.) : 0.3
 mul.:    2, cent.:P, rl: 0, rewind: Y, nr:   199
*
i
       2 solution(s)
                                        L0:   0.0             200kV
   h1 k1 l1   r1  h2 k2 l2    r2  angle  L1-0    c.c.  ---- errors ----  R  mul
  obs.:     28.75           33.19  74.1    0.0  719.4 ang. r1/r2% c.c.%
 ------------------------------------------------------------------------------
   1  3  0 28.777  3  0  1 33.163  74.9  311.5  719.5 -0.9  0.21  0.01  0.68
                                               [    3    -1    -9]

                                     1 equivalent solution(s) not listed
*
pg
 consec. # of data set? 0 or <0: next set (  12 )

-DC-
 dir.lc.:  79.0641  79.0641  38.2168  90.00  90.00  90.00, V(P):238898.3 tetr
 rec.lc.: 0.012648 0.012648 0.026166  90.00  90.00  90.00, SG. P (***)

 P.2.0379
   r1:  23.11 +- 0.69; ang.:   80.27 +-  2.50;   L0:    0.00; wgt(angle): 0.6
   r2:  27.89 +- 0.84; c.c.:  719.42 +- 35.97; L1-0:    0.00; wgt(r1/r2): 0.8
   r3:  33.07 +- 0.00; volt:     200000.     ; V(P):     0.0; wgt(c.c.) : 0.3
 mul.:    2, cent.:P, rl: 0, rewind: Y, nr:   199
*
i
       1 solution(s)
                                        L0:   0.0             200kV
   h1 k1 l1   r1  h2 k2 l2    r2  angle  L1-0    c.c.  ---- errors ----  R  mul
  obs.:     23.11           27.89  80.3    0.0  719.4 ang. r1/r2% c.c.%
```

```
 ------------------------------------------------------------------------------
  -1 -1  1 23.016  2  1  1 27.978  80.3  382.4  726.1 -0.1 -0.71  0.94  0.89
                                                [    -2     3     1]
*
pg
 consec. # of data set? 0 or <0: next set (  13 )

-DC-
 dir.lc.:  79.0641  79.0641  38.2168  90.00  90.00  90.00, V(P):238898.3 tetr
 rec.lc.: 0.012648 0.012648 0.026166  90.00  90.00  90.00, SG. P (***)

 P.2.0610
   r1:  12.85 +- 0.39; ang.:   86.73 +-  2.50;   L0:    0.00; wgt(angle): 0.6
   r2:  86.98 +- 2.61; c.c.:  719.42 +- 35.97; L1-0:    0.00; wgt(r1/r2): 0.8
   r3:  87.20 +- 0.00; volt:     200000.     ; V(P):     0.0; wgt(c.c.) : 0.3
 mul.:    2, cent.:P, rl: 0, rewind: Y, nr:   199
*
i
       2 solution(s)
                                          L0:   0.0             200kV
   h1 k1 l1   r1  h2 k2 l2    r2  angle   L1-0    c.c.  ---- errors ----   R  mul
  obs.:     12.85            86.98  86.7    0.0   719.4 ang. r1/r2% c.c.%
 ------------------------------------------------------------------------------
   1 -1  0 12.740  4  3  4 87.098  85.8  278.6  712.2  0.9 -1.02 -1.00  1.67
                                                [    -4    -4     7]
   1 -1  0 12.997  7  6  1 86.840  85.7  287.5  726.6  1.0  1.28  1.00  1.94
                                                [    -1    -1    13]
*
pg
 consec. # of data set? 0 or <0: next set (  14 )

-DC-
 dir.lc.:  79.0641  79.0641  38.2168  90.00  90.00  90.00, V(P):238898.3 tetr
 rec.lc.: 0.012648 0.012648 0.026166  90.00  90.00  90.00, SG. P (***)

 P.2.0753
   r1:  19.03 +- 0.57; ang.:   88.70 +-  2.50;   L0:    0.00; wgt(angle): 0.6
   r2:  46.52 +- 1.40; c.c.:  719.42 +- 35.97; L1-0:    0.00; wgt(r1/r2): 0.8
   r3:  49.86 +- 0.00; volt:     200000.     ; V(P):     0.0; wgt(c.c.) : 0.3
 mul.:    2, cent.:P, rl: 0, rewind: Y, nr:   199
*
i
       2 solution(s)
                                          L0:   0.0             200kV
   h1 k1 l1   r1  h2 k2 l2    r2  angle   L1-0    c.c.  ---- errors ----   R  mul
  obs.:     19.03            46.52  88.7    0.0   719.4 ang. r1/r2% c.c.%
 ------------------------------------------------------------------------------
   0  0  1 18.920  5  1  0 46.632  90.0  321.7  723.1 -1.3 -0.80  0.51  1.57
                                                [    -1     5     0]
   0  0  1 19.185  4  3  0 46.367  90.0  329.3  733.2 -1.3  1.16  1.91  2.28
                                                [    -3     4     0]
*
pg
 consec. # of data set? 0 or <0: next set (  15 )

-DC-
 dir.lc.:  79.0641  79.0641  38.2168  90.00  90.00  90.00, V(P):238898.3 tetr
 rec.lc.: 0.012648 0.012648 0.026166  90.00  90.00  90.00, SG. P (***)

 P.2.0806
   r1:  31.56 +- 0.95; ang.:   85.59 +-  2.50;   L0:    0.00; wgt(angle): 0.6
   r2:  50.54 +- 1.52; c.c.:  719.42 +- 35.97; L1-0:    0.00; wgt(r1/r2): 0.8
   r3:  57.49 +- 0.00; volt:     200000.     ; V(P):     0.0; wgt(c.c.) : 0.3
 mul.:    2, cent.:P, rl: 0, rewind: Y, nr:   199
*
i
       3 solution(s)
                                          L0:   0.0             200kV
   h1 k1 l1   r1  h2 k2 l2    r2  angle   L1-0    c.c.  ---- errors ----   R  mul
  obs.:     31.56            50.54  85.6    0.0   719.4 ang. r1/r2% c.c.%
```

```
 ------------------------------------------------------------------------------
   2  2 -1 31.994  3  2  2 50.108  85.7  238.1  721.9 -0.1  2.23  0.34  1.96
                                                [     6    -7    -2]
   2  2 -1 30.786  4  1  2 51.316  86.0  222.0  694.6 -0.4 -4.00 -3.45  4.46
                                                [     5    -8    -6]
  -2  2  1 32.271  4  3  1 49.831  83.1  242.5  728.1  2.5  3.65  1.21  4.78
                                                [    -1     6   -14]
*
pg
 consec. # of data set? 0 or <0: next set (  16 )

-DC-
 dir.lc.:  79.0641  79.0641  38.2168  90.00  90.00  90.00, V(P):238898.3 tetr
 rec.lc.: 0.012648 0.012648 0.026166  90.00  90.00  90.00, SG. P (***)

 P.2.0872
   r1:  22.35 +- 0.67; ang.:   82.33 +-  2.50;   L0:    0.00; wgt(angle): 0.6
   r2:  38.87 +- 1.17; c.c.:  719.42 +- 35.97; L1-0:    0.00; wgt(r1/r2): 0.8
   r3:  42.17 +- 0.00; volt:     200000.     ; V(P):     0.0; wgt(c.c.) : 0.3
 mul.:    2, cent.:P, rl: 0, rewind: Y, nr:   199
*
i
       3 solution(s)
                                          L0:   0.0           200kV
   h1 k1 l1   r1  h2 k2 l2    r2  angle   L1-0    c.c.  ---- errors ----   R  mul
  obs.:     22.35           38.87  82.3    0.0  719.4 ang. r1/r2% c.c.%
 ------------------------------------------------------------------------------
  -1  0  1 21.792  3  2  1 39.423  82.3  497.9  749.8  0.0 -3.95  4.23  4.44  2
( -1  0  1         1  1  1;   1,  0;  -1,  2)    [    -1     2    -1]
   0  1 -1 21.907  4  1  0 39.308  83.9  354.8  753.8 -1.6 -3.13  4.77  4.91
                                                [     1    -4    -4]

                                    1 equivalent solution(s) not listed
*
pg
 consec. # of data set? 0 or <0: next set (  17 )

-DC-
 dir.lc.:  79.0641  79.0641  38.2168  90.00  90.00  90.00, V(P):238898.3 tetr
 rec.lc.: 0.012648 0.012648 0.026166  90.00  90.00  90.00, SG. P (***)

 P.2.1099
   r1:  13.06 +- 0.39; ang.:   82.50 +-  2.50;   L0:    0.00; wgt(angle): 0.6
   r2:  38.10 +- 1.14; c.c.:  719.42 +- 35.97; L1-0:    0.00; wgt(r1/r2): 0.8
   r3:  38.62 +- 0.00; volt:     200000.     ; V(P):     0.0; wgt(c.c.) : 0.3
 mul.:    2, cent.:P, rl: 0, rewind: Y, nr:   199
*
i
       3 solution(s)
                                          L0:   0.0           200kV
   h1 k1 l1   r1  h2 k2 l2    r2  angle   L1-0    c.c.  ---- errors ----   R  mul
  obs.:     13.06           38.10  82.5    0.0  719.4 ang. r1/r2% c.c.%
 ------------------------------------------------------------------------------
   1 -1  0 12.985  3  2  1 38.169  80.2  435.7  726.0  2.3 -0.76  0.91  2.25
                                                [    -1    -1     5]
   1  1  0 12.757  1  0  2 38.397  80.4  422.9  713.2  2.1 -3.13 -0.87  4.00
                                                [     2    -2    -1]

                                    1 equivalent solution(s) not listed
*
pg
 consec. # of data set? 0 or <0: next set (  18 )

-DC-
 dir.lc.:  79.0641  79.0641  38.2168  90.00  90.00  90.00, V(P):238898.3 tetr
 rec.lc.: 0.012648 0.012648 0.026166  90.00  90.00  90.00, SG. P (***)

 P.2.1149
   r1:  12.73 +- 0.38; ang.:   87.28 +-  2.50;   L0:    0.00; wgt(angle): 0.6
   r2: 101.50 +- 3.05; c.c.:  719.42 +- 35.97; L1-0:    0.00; wgt(r1/r2): 0.8
```

```
   r3: 101.69 +- 0.00; volt:     200000.     ; V(P):     0.0; wgt(c.c.) : 0.3
 mul.:    2, cent.:P, rl: 0, rewind: Y, nr:   199
*
i
       4 solution(s)
                                      L0:   0.0             200kV
   h1 k1 l1    r1  h2 k2 l2    r2  angle  L1-0   c.c.  ---- errors ----  R  mul
  obs.:     12.73          101.50  87.3    0.0  719.4 ang. r1/r2% c.c.%
 ----------------------------------------------------------------------------
   1 -1  0 12.895  7  6  3101.335  86.4  263.0  720.9  0.9  1.45  0.20  1.78
                                               [     -3    -3     13]
   1 -1  0 12.627  6  5  4101.603  86.4  254.4  706.0  0.8 -0.91 -1.87  1.79
                                               [     -4    -4     11]
   1 -1  0 12.600  8  7  2101.630  86.4  253.6  704.4  0.8 -1.16 -2.09  2.05
                                               [     -2    -2     15]
   1 -1  0 12.520  4  3  5101.710  86.5  251.1  699.9  0.8 -1.87 -2.71  2.80
                                               [     -5    -5      7]
*
ap
  free:      7  8  9 10 11 12 13 14 15 16 17 18 19 20
 which number?
7
  #         d1   s/d(%)    d2  s/d(%)   d3     ang. sig.     V      d-m  seq.
  1 4     79.0641 3.00  79.0641 3.00  55.9068  90.0  2.5      0.0 79.064  1
  2 L     77.4811 3.00   6.4638 3.00   6.4414  90.0  2.5      0.0 22.379  6
  3 L     78.9920 3.00  14.6820 3.00  14.4348  90.0  2.5      0.0 34.055  2
  4 L     77.1198 3.00   9.5287 3.00   9.4568  90.0  2.5      0.0 27.108  4
  5 L     78.2012 3.00   8.5239 3.00   8.4737  90.0  2.5      0.0 25.818  5
  6 L     79.6400 3.00  12.0911 3.00  11.9541  90.0  2.5      0.0 31.031  3
  7 L     55.6827 3.00   8.9815 3.00   8.8669  90.0  2.5      0.0 22.363  7
*
pg
 consec. # of data set? 0 or <0: next set (   8 )

dummy
 dir.lc.:  10.0000  10.0000  10.0000  90.00  90.00  90.00, V(F):  1000.0 cub.
 rec.lc.: 0.100000 0.100000 0.100000  90.00  90.00  90.00, SG. F (***)

P.2.0007
   r1:  13.32 +- 0.40; ang.:   89.70 +-  2.50;   L0:    0.00; wgt(angle): 0.6
   r2:  56.40 +- 1.69; c.c.:  719.42 +- 35.97; L1-0:    0.00; wgt(r1/r2): 0.8
   r3:  57.88 +- 0.00; volt:     200000.     ; V(F):     0.0; wgt(c.c.) : 0.3
 mul.:    2, cent.:F, rl: 0, rewind: Y, nr:   199
*
ap
  free:      8  9 10 11 12 13 14 15 16 17 18 19 20
 which number?
8
  #         d1   s/d(%)    d2  s/d(%)   d3     ang. sig.     V      d-m  seq.
  1 4     79.0641 3.00  79.0641 3.00  55.9068  90.0  2.5      0.0 79.064  1
  2 L     77.4811 3.00   6.4638 3.00   6.4414  90.0  2.5      0.0 22.379  7
  3 L     78.9920 3.00  14.6820 3.00  14.4348  90.0  2.5      0.0 34.055  2
  4 L     77.1198 3.00   9.5287 3.00   9.4568  90.0  2.5      0.0 27.108  4
  5 L     78.2012 3.00   8.5239 3.00   8.4737  90.0  2.5      0.0 25.818  6
  6 L     79.6400 3.00  12.0911 3.00  11.9541  90.0  2.5      0.0 31.031  3
  7 L     55.6827 3.00   8.9815 3.00   8.8669  90.0  2.5      0.0 22.363  8
  8       54.0284 3.00  12.7557 3.00  12.4289  89.7  2.5      0.0 26.252  5
*
pg
 consec. # of data set? 0 or <0: next set (   9 )

dummy
 dir.lc.:  10.0000  10.0000  10.0000  90.00  90.00  90.00, V(F):  1000.0 cub.
 rec.lc.: 0.100000 0.100000 0.100000  90.00  90.00  90.00, SG. F (***)

P.2.0028
   r1:  12.95 +- 0.39; ang.:   73.03 +-  2.50;   L0:    0.00; wgt(angle): 0.6
   r2:  20.88 +- 0.63; c.c.:  719.42 +- 35.97; L1-0:    0.00; wgt(r1/r2): 0.8
   r3:  21.11 +- 0.00; volt:     200000.     ; V(F):     0.0; wgt(c.c.) : 0.3
 mul.:    2, cent.:F, rl: 0, rewind: Y, nr:   199
```

```
*
ap
  free:       9 10 11 12 13 14 15 16 17 18 19 20
 which number?
9
  #         d1   s/d(%)    d2   s/d(%)    d3     ang. sig.      V      d-m   seq.
  1 4      79.0641 3.00  79.0641 3.00  55.9068  90.0  2.5      0.0 79.064  1
  2 L      77.4811 3.00   6.4638 3.00   6.4414  90.0  2.5      0.0 22.379  8
  3 L      78.9920 3.00  14.6820 3.00  14.4348  90.0  2.5      0.0 34.055  3
  4 L      77.1198 3.00   9.5287 3.00   9.4568  90.0  2.5      0.0 27.108  5
  5 L      78.2012 3.00   8.5239 3.00   8.4737  90.0  2.5      0.0 25.818  7
  6 L      79.6400 3.00  12.0911 3.00  11.9541  90.0  2.5      0.0 31.031  4
  7 L      55.6827 3.00   8.9815 3.00   8.8669  90.0  2.5      0.0 22.363  9
  8        54.0284 3.00  12.7557 3.00  12.4289  89.7  2.5      0.0 26.252  6
  9        55.5395 3.00  34.4603 3.00  34.0743  73.0  2.5      0.0 44.733  2
*
pg
 consec. # of data set? 0 or <0: next set (  10 )

dummy
 dir.lc.:  10.0000  10.0000  10.0000  90.00  90.00  90.00, V(F):  1000.0 cub.
 rec.lc.: 0.100000 0.100000 0.100000  90.00  90.00  90.00, SG. F (***)

 P.2.0103
   r1:  28.48 +- 0.85; ang.:   86.34 +-  2.50;   L0:    0.00; wgt(angle): 0.6
   r2:  42.49 +- 1.27; c.c.:  719.42 +- 35.97; L1-0:    0.00; wgt(r1/r2): 0.8
   r3:  49.62 +- 0.00; volt:     200000.     ; V(F):     0.0; wgt(c.c.) : 0.3
 mul.:    2, cent.:F, rl: 0, rewind: Y, nr:   199
*
ax
  #         d1   s/d(%)    d2   s/d(%)    d3     ang. sig.      V      d-m   seq.
  1 4      79.0641 3.00  79.0641 3.00  55.9068  90.0  2.5      0.0 79.064  1
  2 L      77.4811 3.00   6.4638 3.00   6.4414  90.0  2.5      0.0 22.379  8
  3 L      78.9920 3.00  14.6820 3.00  14.4348  90.0  2.5      0.0 34.055  3
  4 L      77.1198 3.00   9.5287 3.00   9.4568  90.0  2.5      0.0 27.108  5
  5 L      78.2012 3.00   8.5239 3.00   8.4737  90.0  2.5      0.0 25.818  7
  6 L      79.6400 3.00  12.0911 3.00  11.9541  90.0  2.5      0.0 31.031  4
  7 L      55.6827 3.00   8.9815 3.00   8.8669  90.0  2.5      0.0 22.363  9
  8        54.0284 3.00  12.7557 3.00  12.4289  89.7  2.5      0.0 26.252  6
  9        55.5395 3.00  34.4603 3.00  34.0743  73.0  2.5      0.0 44.733  2
 exclude or (<0) include which number?
 abs. value > 20 : exclude or include all
1
  #         d1   s/d(%)    d2   s/d(%)    d3     ang. sig.      V      d-m   seq.
  1 4 e    79.0641 3.00  79.0641 3.00  55.9068  90.0  2.5      0.0  0.000  9
  2 L      77.4811 3.00   6.4638 3.00   6.4414  90.0  2.5      0.0 22.379  7
  3 L      78.9920 3.00  14.6820 3.00  14.4348  90.0  2.5      0.0 34.055  2
  4 L      77.1198 3.00   9.5287 3.00   9.4568  90.0  2.5      0.0 27.108  4
  5 L      78.2012 3.00   8.5239 3.00   8.4737  90.0  2.5      0.0 25.818  6
  6 L      79.6400 3.00  12.0911 3.00  11.9541  90.0  2.5      0.0 31.031  3
  7 L      55.6827 3.00   8.9815 3.00   8.8669  90.0  2.5      0.0 22.363  8
  8        54.0284 3.00  12.7557 3.00  12.4289  89.7  2.5      0.0 26.252  5
  9        55.5395 3.00  34.4603 3.00  34.0743  73.0  2.5      0.0 44.733  1
*
ax
  #         d1   s/d(%)    d2   s/d(%)    d3     ang. sig.      V      d-m   seq.
  1 4 e    79.0641 3.00  79.0641 3.00  55.9068  90.0  2.5      0.0  0.000  9
  2 L      77.4811 3.00   6.4638 3.00   6.4414  90.0  2.5      0.0 22.379  7
  3 L      78.9920 3.00  14.6820 3.00  14.4348  90.0  2.5      0.0 34.055  2
  4 L      77.1198 3.00   9.5287 3.00   9.4568  90.0  2.5      0.0 27.108  4
  5 L      78.2012 3.00   8.5239 3.00   8.4737  90.0  2.5      0.0 25.818  6
  6 L      79.6400 3.00  12.0911 3.00  11.9541  90.0  2.5      0.0 31.031  3
  7 L      55.6827 3.00   8.9815 3.00   8.8669  90.0  2.5      0.0 22.363  8
  8        54.0284 3.00  12.7557 3.00  12.4289  89.7  2.5      0.0 26.252  5
  9        55.5395 3.00  34.4603 3.00  34.0743  73.0  2.5      0.0 44.733  1
 exclude or (<0) include which number?
 abs. value > 20 : exclude or include all
7
  #         d1   s/d(%)    d2   s/d(%)    d3     ang. sig.      V      d-m   seq.
  1 4 e    79.0641 3.00  79.0641 3.00  55.9068  90.0  2.5      0.0  0.000  8
```

```
   2 L     77.4811 3.00   6.4638 3.00   6.4414  90.0  2.5      0.0 22.379  7
   3 L     78.9920 3.00  14.6820 3.00  14.4348  90.0  2.5      0.0 34.055  2
   4 L     77.1198 3.00   9.5287 3.00   9.4568  90.0  2.5      0.0 27.108  4
   5 L     78.2012 3.00   8.5239 3.00   8.4737  90.0  2.5      0.0 25.818  6
   6 L     79.6400 3.00  12.0911 3.00  11.9541  90.0  2.5      0.0 31.031  3
   7 L e   55.6827 3.00   8.9815 3.00   8.8669  90.0  2.5      0.0  0.000  9
   8       54.0284 3.00  12.7557 3.00  12.4289  89.7  2.5      0.0 26.252  5
   9       55.5395 3.00  34.4603 3.00  34.0743  73.0  2.5      0.0 44.733  1
 *
 ax
   #         d1   s/d(%)    d2  s/d(%)   d3      ang. sig.     V      d-m  seq.
   1 4 e   79.0641 3.00  79.0641 3.00  55.9068  90.0  2.5      0.0  0.000  8
   2 L     77.4811 3.00   6.4638 3.00   6.4414  90.0  2.5      0.0 22.379  7
   3 L     78.9920 3.00  14.6820 3.00  14.4348  90.0  2.5      0.0 34.055  2
   4 L     77.1198 3.00   9.5287 3.00   9.4568  90.0  2.5      0.0 27.108  4
   5 L     78.2012 3.00   8.5239 3.00   8.4737  90.0  2.5      0.0 25.818  6
   6 L     79.6400 3.00  12.0911 3.00  11.9541  90.0  2.5      0.0 31.031  3
   7 L e   55.6827 3.00   8.9815 3.00   8.8669  90.0  2.5      0.0  0.000  9
   8       54.0284 3.00  12.7557 3.00  12.4289  89.7  2.5      0.0 26.252  5
   9       55.5395 3.00  34.4603 3.00  34.0743  73.0  2.5      0.0 44.733  1
  exclude or (<0) include which number?
  abs. value > 20 : exclude or include all
 8
   #         d1   s/d(%)    d2  s/d(%)   d3      ang. sig.     V      d-m  seq.
   1 4 e   79.0641 3.00  79.0641 3.00  55.9068  90.0  2.5      0.0  0.000  7
   2 L     77.4811 3.00   6.4638 3.00   6.4414  90.0  2.5      0.0 22.379  6
   3 L     78.9920 3.00  14.6820 3.00  14.4348  90.0  2.5      0.0 34.055  2
   4 L     77.1198 3.00   9.5287 3.00   9.4568  90.0  2.5      0.0 27.108  4
   5 L     78.2012 3.00   8.5239 3.00   8.4737  90.0  2.5      0.0 25.818  5
   6 L     79.6400 3.00  12.0911 3.00  11.9541  90.0  2.5      0.0 31.031  3
   7 L e   55.6827 3.00   8.9815 3.00   8.8669  90.0  2.5      0.0  0.000  8
   8   e   54.0284 3.00  12.7557 3.00  12.4289  89.7  2.5      0.0  0.000  9
   9       55.5395 3.00  34.4603 3.00  34.0743  73.0  2.5      0.0 44.733  1
 *
 pc
   #         d1   s/d(%)    d2  s/d(%)   d3      ang. sig.     V      d-m  seq.
   1 4 e   79.0641 3.00  79.0641 3.00  55.9068  90.0  2.5      0.0  0.000  6
   2 L     77.4811 3.00   6.4638 3.00   6.4414  90.0  2.5      0.0 22.379  5
   3 L     78.9920 3.00  14.6820 3.00  14.4348  90.0  2.5      0.0 34.055  1
   4 L     77.1198 3.00   9.5287 3.00   9.4568  90.0  2.5      0.0 27.108  3
   5 L     78.2012 3.00   8.5239 3.00   8.4737  90.0  2.5      0.0 25.818  4
   6 L     79.6400 3.00  12.0911 3.00  11.9541  90.0  2.5      0.0 31.031  2
   7 L e   55.6827 3.00   8.9815 3.00   8.8669  90.0  2.5      0.0  0.000  7
   8   e   54.0284 3.00  12.7557 3.00  12.4289  89.7  2.5      0.0  0.000  8
   9       55.5395 3.00  34.4603 3.00  34.0743  73.0  2.5      0.0 44.733  0

  a*,b*-defining:  3;L
        sequence:       9;   6;L  4;L  5;L  2;L

    1st: new a*,b* defining number (0: no changes)
    2nd: <0: enforce full grid, >0: * sigma (0.87mm) = "wall thickness"


  V(P)(min), V(P)(max)? (calc.: min:     0. max:     0., mean:     0.)
  def.:     0.     0.
 0 300000
   36862.300000.
  factor for default increment (0.025), def.:1., max:6; <0 : increment

     0.025

      29492 sets within  84 layers, p:  0.933 -  7.596
 *
 dc
   mult.<mu>: 2; <rl>: 0;  1st layer: V:******; n:  102; p:  0.933
   ok?


       45 solutions stored,  R :     0.60 -   2.84, incl. equiv.:      434
       R      a     b     c     al    be    ga      x      y      V     int.
```

```
  1  0.60 38.48 78.65 78.99   90.0   90.0   91.4  0.000  0.173238994.4  0.44
  2  0.75 36.51 59.19 78.99   90.0   90.0 106.6  0.000  0.237163618.0  0.38
  3  0.80 39.22 78.99 81.28   90.0   93.4   90.0  0.000  0.168251378.8  0.32
  4  0.80 36.28 71.70 78.99   90.0   90.0   91.9  0.000  0.440205383.3  0.84
  5  0.83 39.92 58.84 78.99   90.0   90.0 108.0  0.000  0.244176499.1  0.49
  6  0.86 57.04 70.07 78.99   90.0   90.0 108.1  0.000  0.213300000.3  0.51
  7  0.86 61.76 66.89 78.99   90.0   90.0 113.2  0.000  0.268300000.4  0.22
  8  0.86 59.62 63.81 84.43   88.8   69.3   86.6  0.500  0.240300000.3  0.16
  9  0.89 48.12 79.61 82.58   85.7   73.1   75.2  0.500  0.424292517.2  0.41
 10  0.92 46.42 59.05 86.92  108.7 104.1   94.0  0.500  0.307216026.0  0.29
 11  0.97 44.23 59.52 86.07  107.8 101.7   99.0  0.500  0.326205383.3  0.45
 12  1.00 45.83 82.25 82.72   86.6   77.6   73.8  0.500  0.430292517.1  0.14
 13  1.00 39.51 78.99 83.07   90.0   96.1   90.0  0.000  0.165257809.6  0.39
 14  1.12 48.16 78.40 82.58   85.1   73.0   73.0  0.500  0.422285220.6  0.25
 15  1.13 46.40 79.33 82.33   85.3   73.6   73.0  0.500  0.425278106.0  0.24
 16  1.18 42.28 58.75 78.99   90.0   90.0 108.9  0.000  0.248185645.0  0.18
 17  1.20 47.04 61.58 87.01  108.8 103.2   96.8  0.500  0.305227220.2  0.33
 18  1.23 46.40 81.89 82.33   86.4   73.6   77.0  0.500  0.429292517.2  0.19
 19  1.23 55.97 57.32 84.03   70.1   87.5   82.7  0.500  0.386251378.8  0.12
 20  1.23 57.96 69.98 78.99   90.0   90.0 110.6  0.000  0.403300000.3  0.18
 21  1.27 34.44 59.50 84.41   69.4   84.4   74.0  0.500  0.232155557.2  0.18
 22  1.27 56.64 68.11 86.02   66.7   82.0   69.4  0.500  0.285285220.6  0.28
 23  1.29 59.19 64.52 84.35   87.9   69.5   84.0  0.500  0.243300000.3  0.11
 24  1.31 34.26 55.32 78.99   90.0   90.0 105.6  0.000  0.429144204.5  1.00
 25  1.35 39.99 72.22 81.48   88.7   75.8   84.8  0.500  0.431227220.1  0.06
 26  1.37 57.26 57.79 84.02   86.8   70.1   80.5  0.500  0.389257809.6  0.17
 27  1.41 45.78 61.30 87.49  110.0 104.5   92.0  0.500  0.312221552.4  0.18
 28  1.44 61.29 65.67 78.99   90.0   90.0 109.4  0.000  0.278300000.4  0.28
 29  1.55 40.20 77.30 78.99   90.0   90.0   93.2  0.000  0.177245108.4  0.10
 30  1.58 36.84 59.50 84.41   69.4   85.0   75.7  0.500  0.234167803.7  0.25
 31  1.58 37.14 81.15 88.74   88.2   82.0   76.8  0.500  0.449257809.6  0.31
 32  1.58 39.87 78.99 91.17   90.0   96.7   90.0  0.000  0.151285220.6  0.16
 33  1.60 38.45 60.36 84.56   69.1   86.8   80.9  0.500  0.228181014.3  0.11
 34  1.63 39.82 60.90 84.66   68.9   87.7   83.7  0.500  0.225190394.2  0.19
 35  1.63 60.47 61.31 84.73   68.8   89.0   87.4  0.500  0.233292517.1  0.05
 36  1.65 44.44 61.15 78.99   90.0   90.0 111.1  0.000  0.345200260.2  0.05
cont.?
n
*
```

S5. PIEP run for unit cell parameters determination for GRGDS

S5.1. Input file (sad.dat) for GRGDS

This file contains measured values for the camera constant, the measured lengths of two vectors in each diffraction pattern and angles between the vectors.

```
  1 2.32
358.9000  17.5930  25.9724   0.7792  81.7823   2.4535
 81.7925   0.0000  80.8861   2.5000     0.00     0.00     0.00
    0.00     5.00     0.00     5.00  200000.
  2 2.71
358.9000  17.5930  27.7292   0.8319  91.8230   2.7547
 93.8435   0.0000  85.5651   2.5000     0.00     0.00     0.00
    0.00     5.00     0.00     5.00  200000.
  3 3.21
358.9000  17.5930  50.5200   1.5156  81.2344   2.4370
 88.5512   0.0000  80.8163   2.5000     0.00     0.00     0.00
    0.00     5.00     0.00     5.00  200000.
  4 4.39
358.9000  17.5930  75.3997   2.2620  81.6313   2.4489
101.5291   0.0000  80.4580   2.5000     0.00     0.00     0.00
    0.00     5.00     0.00     5.00  200000.
  5 3.3
```

```
  358.9000  17.5930  27.6289   0.8289 244.3731   7.3312
  245.4994   0.0000  89.1019   2.5000     0.00     0.00     0.00
      0.00     5.00     0.00     5.00  200000.
END$
```

S5.2. Communication protocol with PIEP

```
                #################################
                =======      P I E P      =======
                ======= VERSION 14-jun-17 =======
                #################################

 default parameters from file? (def.=yes)

 parameter-file piep.par            ? (blank), otherwise name

 ------------------------------------------------------------------------
  cell parameter file assigned: cell.dat            ,    21 sets
  1st set read, unit: 20, file: cell.dat
 ------------------------------------------------------------------------
  SAD data file: unit 30, file: sad.dat             ,     5 sets
  1st set loaded
 ------------------------------------------------------------------------

dummy
 dir.lc.:  10.0000  10.0000  10.0000  90.00  90.00  90.00, V(F):  1000.0 cub.
 rec.lc.: 0.100000 0.100000 0.100000  90.00  90.00  90.00, SG. F (***)

 2.32
   r1:  25.97 +- 0.78; ang.:   80.89 +-  2.50;   L0:    0.00; wgt(angle): 0.6
   r2:  81.78 +- 2.45; c.c.:  358.90 +- 17.59; L1-0:    0.00; wgt(r1/r2): 0.8
   r3:  81.79 +- 0.00; volt:     200000.     ; V(F):     0.0; wgt(c.c.) : 0.3
 mul.:    2, cent.:F, rl: 0, rewind: Y, nr:   199
*
ap
  free:      1  2  3  4  5  6  7  8  9 10 11 12 13 14 15 16 17 18 19 20
 which number?
1
  #         d1   s/d(%)    d2  s/d(%)    d3     ang. sig.     V      d-m  seq.
  1 V     13.8185 3.00   4.3885 3.00   4.3879  80.9   2.5      0.0  7.837  1
*
pg
 consec. # of data set? 0 or <0: next set (   2 )

dummy
 dir.lc.:  10.0000  10.0000  10.0000  90.00  90.00  90.00, V(F):  1000.0 cub.
 rec.lc.: 0.100000 0.100000 0.100000  90.00  90.00  90.00, SG. F (***)

 2.71
   r1:  27.73 +- 0.83; ang.:   85.57 +-  2.50;   L0:    0.00; wgt(angle): 0.6
   r2:  91.82 +- 2.75; c.c.:  358.90 +- 17.59; L1-0:    0.00; wgt(r1/r2): 0.8
   r3:  93.84 +- 0.00; volt:     200000.     ; V(F):     0.0; wgt(c.c.) : 0.3
 mul.:    2, cent.:F, rl: 0, rewind: Y, nr:   199
*
ap
  free:      2  3  4  5  6  7  8  9 10 11 12 13 14 15 16 17 18 19 20
 which number?
2
  #         d1   s/d(%)    d2  s/d(%)    d3     ang. sig.     V      d-m  seq.
  1 V     13.8185 3.00   4.3885 3.00   4.3879  80.9   2.5      0.0  7.837  1
  2       12.9430 3.00   3.9086 3.00   3.8245  85.6   2.5      0.0  7.123  2
*
pg
 consec. # of data set? 0 or <0: next set (   3 )

dummy
```

```
 dir.lc.:  10.0000  10.0000  10.0000  90.00  90.00  90.00, V(F):  1000.0 cub.
 rec.lc.: 0.100000 0.100000 0.100000  90.00  90.00  90.00, SG. F (***)

 3.21
   r1:  50.52 +- 1.52; ang.:   80.82 +-  2.50;   L0:    0.00; wgt(angle): 0.6
   r2:  81.23 +- 2.44; c.c.:  358.90 +- 17.59; L1-0:    0.00; wgt(r1/r2): 0.8
   r3:  88.55 +- 0.00; volt:     200000.     ; V(F):     0.0; wgt(c.c.) : 0.3
 mul.:    2, cent.:F, rl: 0, rewind: Y, nr:   199
*
ap
  free:      3  4  5  6  7  8  9 10 11 12 13 14 15 16 17 18 19 20
 which number?
3
  #         d1   s/d(%)    d2  s/d(%)   d3     ang. sig.     V      d-m  seq.
  1 V     13.8185 3.00   4.3885 3.00   4.3879  80.9  2.5      0.0  7.837  1
  2       12.9430 3.00   3.9086 3.00   3.8245  85.6  2.5      0.0  7.123  2
  3        7.1041 3.00   4.4181 3.00   4.0530  80.8  2.5      0.0  5.639  3
*
pg
 consec. # of data set? 0 or <0: next set (   4 )

dummy
 dir.lc.:  10.0000  10.0000  10.0000  90.00  90.00  90.00, V(F):  1000.0 cub.
 rec.lc.: 0.100000 0.100000 0.100000  90.00  90.00  90.00, SG. F (***)

 4.39
   r1:  75.40 +- 2.26; ang.:   80.46 +-  2.50;   L0:    0.00; wgt(angle): 0.6
   r2:  81.63 +- 2.45; c.c.:  358.90 +- 17.59; L1-0:    0.00; wgt(r1/r2): 0.8
   r3: 101.53 +- 0.00; volt:     200000.     ; V(F):     0.0; wgt(c.c.) : 0.3
 mul.:    2, cent.:F, rl: 0, rewind: Y, nr:   199
*
ap
  free:      4  5  6  7  8  9 10 11 12 13 14 15 16 17 18 19 20
 which number?
4
  #         d1   s/d(%)    d2  s/d(%)   d3     ang. sig.     V      d-m  seq.
  1 V     13.8185 3.00   4.3885 3.00   4.3879  80.9  2.5      0.0  7.837  1
  2       12.9430 3.00   3.9086 3.00   3.8245  85.6  2.5      0.0  7.123  2
  3        7.1041 3.00   4.4181 3.00   4.0530  80.8  2.5      0.0  5.639  3
  4        4.7600 3.00   4.3966 3.00   3.5349  80.5  2.5      0.0  4.607  4
*
pg
 consec. # of data set? 0 or <0: next set (   5 )

dummy
 dir.lc.:  10.0000  10.0000  10.0000  90.00  90.00  90.00, V(F):  1000.0 cub.
 rec.lc.: 0.100000 0.100000 0.100000  90.00  90.00  90.00, SG. F (***)

 3.3
   r1:  27.63 +- 0.83; ang.:   89.10 +-  2.50;   L0:    0.00; wgt(angle): 0.6
   r2: 244.37 +- 7.33; c.c.:  358.90 +- 17.59; L1-0:    0.00; wgt(r1/r2): 0.8
   r3: 245.50 +- 0.00; volt:     200000.     ; V(F):     0.0; wgt(c.c.) : 0.3
 mul.:    2, cent.:F, rl: 0, rewind: Y, nr:   199
*
ap
  free:      5  6  7  8  9 10 11 12 13 14 15 16 17 18 19 20
 which number?
5
  #         d1   s/d(%)    d2  s/d(%)   d3     ang. sig.     V      d-m  seq.
  1 V     13.8185 3.00   4.3885 3.00   4.3879  80.9  2.5      0.0  7.837  1
  2       12.9430 3.00   3.9086 3.00   3.8245  85.6  2.5      0.0  7.123  2
  3        7.1041 3.00   4.4181 3.00   4.0530  80.8  2.5      0.0  5.639  3
  4        4.7600 3.00   4.3966 3.00   3.5349  80.5  2.5      0.0  4.607  4
  5       12.9900 3.00   1.4687 3.00   1.4619  89.1  2.5      0.0  4.368  5
*
pc
  #         d1   s/d(%)    d2  s/d(%)   d3     ang. sig.     V      d-m  seq.
  1 V     13.8185 3.00   4.3885 3.00   4.3879  80.9  2.5      0.0  7.837  0
  2       12.9430 3.00   3.9086 3.00   3.8245  85.6  2.5      0.0  7.123  1
  3        7.1041 3.00   4.4181 3.00   4.0530  80.8  2.5      0.0  5.639  2
```

```
  4        4.7600 3.00   4.3966 3.00   3.5349  80.5  2.5      0.0  4.607  3
  5       12.9900 3.00   1.4687 3.00   1.4619  89.1  2.5      0.0  4.368  4

 a*,b*-defining:  1;V
       sequence:       2;   3;   4;   5;

   1st: new a*,b* defining number (0: no changes)
   2nd: <0: enforce full grid, >0: * sigma (1.62mm) = "wall thickness"


 V(P)(min), V(P)(max)? (calc.: min:     0. max:     0., mean:     0.)
 def.:     0.     0.
0 1500
    251.  1500.
 factor for default increment (0.025), def.:1., max:6; <0 : increment

    0.025

      8642 sets within  72 layers, p:  0.521 -  3.116
*
dc
  mult.<mu>: 2; <rl>: 0;  1st layer: V: 250.5; n:   43; p:  0.521
  ok?


       52 solutions stored,  R :     0.84 -   4.24, incl. equiv.:       297
        R      a     b     c     al     be     ga      x      y       V    int.
   1  0.84   4.44 14.51 19.47  105.3  90.0  98.8  0.196  0.000  1195.6  0.42
   2  0.99   4.44 14.49 20.47  105.0  90.0  98.8  0.183  0.000  1257.4  0.59
   3  1.14   4.44 14.42 21.53   76.1  90.0  81.2  0.161  0.000  1322.4  0.13
   4  1.16   7.82 13.21 14.36   86.5  74.2  76.9  0.000  0.284  1390.8  0.70
   5  1.17   4.44 14.52 18.98   74.6  90.0  81.2  0.204  0.000  1165.8  0.47
   6  1.17   4.47 13.34 14.00   89.1  80.8  84.3  0.000  0.033   819.2  1.00
   7  1.33   4.44 14.10 24.42   82.9  90.0  81.0  0.071  0.000  1500.0  0.12
   8  1.40   4.44 14.10 23.82   97.1  90.0  99.0  0.074  0.000  1462.7  0.42
   9  1.41   4.44 14.34 21.53  102.6  90.0  98.9  0.145  0.000  1322.4  0.20
  10  1.43   4.44 14.59 18.51  106.4  90.0  98.7  0.222  0.000  1136.8  0.42
  11  1.50   4.58 14.70 15.65   62.0  88.4  86.5  0.500  0.074   929.2  0.64
  12  1.53   4.44 13.68 14.49  105.0  98.8  90.0  0.275  0.000   840.1  0.11
  13  1.53   4.83 15.09 15.74   61.4  87.1  84.0  0.500  0.116  1002.2  0.33
  14  1.58   4.44 14.21 24.42   80.1  90.0  81.0  0.100  0.000  1500.0  0.10
  15  1.66   5.13 14.62 15.69  116.4  95.9  97.5  0.500  0.153  1027.8  0.66
  16  1.67   6.77 14.39 15.65  114.8  97.4 100.8  0.500  0.237  1322.4  0.47
  17  1.69   4.44 13.68 14.41   76.3  81.1  90.0  0.250  0.000   840.1  0.26
  18  1.73   4.44 14.10 23.22   83.0  90.0  81.0  0.074  0.000  1426.3  0.19
  19  1.82   4.48 14.75 15.69  116.9  94.8  97.2  0.500  0.038   906.1  0.43
  20  1.86   4.44 13.34 14.27  101.3  98.9  90.0  0.211  0.000   819.2  0.33
  21  1.91   4.44 13.34 14.21   80.0  81.0  90.0  0.184  0.000   819.2  0.32
  22  1.93   6.95 15.44 15.83   60.8  85.4  80.5  0.500  0.224  1462.7  0.40
  23  1.98   8.21 13.39 14.41   85.6  73.5  74.4  0.000  0.290  1462.7  0.17
  24  2.02   7.04 14.26 15.60  114.1  97.3 102.3  0.500  0.247  1356.1  0.24
  25  2.07   4.44 14.80 18.51  108.9  90.0  98.6  0.259  0.000  1136.8  0.09
  26  2.09   5.07 14.18 15.53  115.3  94.7 100.1  0.500  0.153   977.2  0.30
  27  2.17   4.44 14.81 18.05   70.9  90.0  81.4  0.269  0.000  1108.5  0.08
  28  2.22   5.48 13.01 14.09   88.6  78.8  82.7  0.000  0.201   977.2  0.24
  29  2.23   4.44 14.15 21.00   81.5  90.0  81.0  0.100  0.000  1289.5  0.16
  30  2.24   5.19 15.14 15.86  117.6  97.0  94.9  0.500  0.152  1080.9  0.44
  31  2.25   4.44 14.96 15.52  110.7  90.0  98.5  0.341  0.000   952.9  0.31
  32  2.25   4.44 13.34 14.07   95.7  99.1  90.0  0.105  0.000   819.2  0.28
  33  2.28   4.44 14.03 24.42   94.3  90.0  99.1  0.043  0.000  1500.0  0.08
  34  2.29   7.64 14.53 15.61   62.3  84.4  77.8  0.500  0.255  1500.0  0.22
  35  2.42   5.70 13.08 14.11   89.2  78.4  86.0  0.000  0.213  1027.8  0.27
  36  2.45   5.01 14.30 15.56   62.6  85.7  80.5  0.500  0.146   977.2  0.14
 cont.?
n
*
de
 one line: # of transf. to be loaded (0: list all transf.)
           write matrix? (0:no, >0:yes)
           minim. symmetry? (0(=def.):trk.,1:mcl.,2:orh.,3:tet.,4:hex.,5:cub.)
```

```
0
      1       P     4.445    14.508    19.466   105.28    90.00    98.79
      2   mcl A    19.466     4.445    28.676    90.02   105.47    90.00
*
de
 one line: # of transf. to be loaded (0: list all transf.)
           write matrix? (0:no, >0:yes)
           minim. symmetry? (0(=def.):trk.,1:mcl.,2:orh.,3:tet.,4:hex.,5:cub.)
2
                   0.00  0.00 -1.00;   1.00  0.00  0.00;  -1.00 -2.00  0.00;

 dir.lc.:  19.4660   4.4446  28.6756  90.02 105.47  90.00, V(A):  2391.1 trik
 rec.lc.: 0.053302 0.224993 0.036183  89.98  74.53  89.99, SG. A (***)
*
mv
 h  for help

 current cell parameters:
    19.466     4.445    28.676     90.02  105.47   90.00

  matrix 1                               matrix 2
  1.00000     0.00000     0.00000        1.00000     0.00000     0.00000
  0.00000     1.00000     0.00000        0.00000     1.00000     0.00000
  0.00000     0.00000     1.00000        0.00000     0.00000     1.00000
 det1:  1.000000                        det2:  1.000000

  vektor:  1.000     0.000     0.000

   v ,m1,m2,i1,i2,mi,mm,vm,mv,vv,vs,ma,mr,l ,en; h  for help
m1
 matrix 1?
0 0 1 0 -1 0 1 0 0

  matrix 1                               matrix 2
  0.00000     0.00000     1.00000        1.00000     0.00000     0.00000
  0.00000    -1.00000     0.00000        0.00000     1.00000     0.00000
  1.00000     0.00000     0.00000        0.00000     0.00000     1.00000
 det1:  1.000000                        det2:  1.000000

   v ,m1,m2,i1,i2,mi,mm,vm,mv,vv,vs,ma,mr,l ,en; h  for help
ma
 current cell parameters:
    19.466     4.445    28.676     90.02  105.47   90.00
 apply matrix 1?

 transformed cell parameters:
    28.676     4.445    19.466     90.00  105.47   89.98
  replace current cell parameters?

 dir.lc.:  28.6756   4.4446  19.4660  90.00 105.47  89.98, V(A):  2391.1 trik
 rec.lc.: 0.036183 0.224993 0.053302  90.01  74.53  90.02, SG. A (***)
 centering (col.1) (or space group)? (P,A,B,C,R,I,F)
C
 space group: C (***)
 h  for help

 current cell parameters:
    28.676     4.445    19.466     90.00  105.47   89.98

  matrix 1                               matrix 2
  0.00000     0.00000     1.00000        1.00000     0.00000     0.00000
  0.00000    -1.00000     0.00000        0.00000     1.00000     0.00000
  1.00000     0.00000     0.00000        0.00000     0.00000     1.00000
 det1:  1.000000                        det2:  1.000000

  vektor:  1.000     0.000     0.000

   v ,m1,m2,i1,i2,mi,mm,vm,mv,vv,vs,ma,mr,l ,en; h  for help
en
current cell:  28.6756   4.4446  19.4660   90.000  105.47   89.98
```

```
               check centering!
*
pg
 consec. # of data set? 0 or <0: next set (   6 )
1
-DC-
 dir.lc.:  28.6756   4.4446  19.4660  90.00 105.47  89.98, V(C):  2391.1 trik
 rec.lc.: 0.036183 0.224993 0.053302  90.01  74.53  90.02, SG. C (***)

 2.32
   r1:  25.97 +- 0.78; ang.:   80.89 +-  2.50;   L0:    0.00; wgt(angle): 0.6
   r2:  81.78 +- 2.45; c.c.:  358.90 +- 17.59; L1-0:    0.00; wgt(r1/r2): 0.8
   r3:  81.79 +- 0.00; volt:     200000.     ; V(C):     0.0; wgt(c.c.) : 0.3
 mul.:    2, cent.:C, rl: 0, rewind: Y, nr:   199
*
i
       2 solution(s)
                                           L0:   0.0             200kV
   h1 k1 l1    r1  h2 k2 l2    r2  angle   L1-0    c.c.  ---- errors ----  R  mul
  obs.:     25.97            81.78  80.9     0.0  358.9 ang. r1/r2% c.c.%
 ------------------------------------------------------------------------------
   2  0  0 25.972  1  1  0 81.782  80.9  727.1  358.9  0.0 -0.00  0.00  0.00
                                                  [     0     0     1]
   2  0  0 25.970  1 -1  0 81.785  80.8  727.1  358.9  0.0 -0.01 -0.01  0.04
                                                  [     0     0    -1]
*
pg
 consec. # of data set? 0 or <0: next set (   2 )

-DC-
 dir.lc.:  28.6756   4.4446  19.4660  90.00 105.47  89.98, V(C):  2391.1 trik
 rec.lc.: 0.036183 0.224993 0.053302  90.01  74.53  90.02, SG. C (***)

 2.71
   r1:  27.73 +- 0.83; ang.:   85.57 +-  2.50;   L0:    0.00; wgt(angle): 0.6
   r2:  91.82 +- 2.75; c.c.:  358.90 +- 17.59; L1-0:    0.00; wgt(r1/r2): 0.8
   r3:  93.84 +- 0.00; volt:     200000.     ; V(C):     0.0; wgt(c.c.) : 0.3
 mul.:    2, cent.:C, rl: 0, rewind: Y, nr:   199
*
i
       2 solution(s)
                                           L0:   0.0             200kV
   h1 k1 l1    r1  h2 k2 l2    r2  angle   L1-0    c.c.  ---- errors ----  R  mul
  obs.:     27.73            91.82  85.6     0.0  358.9 ang. r1/r2% c.c.%
 ------------------------------------------------------------------------------
  -2  0  1 27.839  1  1  2 91.713  85.6  659.5  358.8 -0.0  0.52 -0.03  0.44
                                                  [    -1     5    -2]
  -2  0  1 27.836  1 -1  2 91.717  85.6  659.3  358.7 -0.1  0.50 -0.05  0.44
                                                  [     1     5     2]
*
pg
 consec. # of data set? 0 or <0: next set (   3 )

-DC-
 dir.lc.:  28.6756   4.4446  19.4660  90.00 105.47  89.98, V(C):  2391.1 trik
 rec.lc.: 0.036183 0.224993 0.053302  90.01  74.53  90.02, SG. C (***)

 3.21
   r1:  50.52 +- 1.52; ang.:   80.82 +-  2.50;   L0:    0.00; wgt(angle): 0.6
   r2:  81.23 +- 2.44; c.c.:  358.90 +- 17.59; L1-0:    0.00; wgt(r1/r2): 0.8
   r3:  88.55 +- 0.00; volt:     200000.     ; V(C):     0.0; wgt(c.c.) : 0.3
 mul.:    2, cent.:C, rl: 0, rewind: Y, nr:   199
*
i
       8 solution(s)
                                           L0:   0.0             200kV
   h1 k1 l1    r1  h2 k2 l2    r2  angle   L1-0    c.c.  ---- errors ----  R  mul
  obs.:     50.52            81.23  80.8     0.0  358.9 ang. r1/r2% c.c.%
 ------------------------------------------------------------------------------
  -4  0  1 50.197 -1  1  0 81.558  81.5  520.1  357.9 -0.7 -1.04 -0.29  1.32
```

```
                                                [     -1     -1     -4]
   4  0 -1 50.200  1  1  0 81.554  81.5  520.1  357.9 -0.7 -1.03 -0.28  1.33
                                                [      1     -1      4]
   4  0  0 51.175  1 -1  0 80.580  80.8  716.4  353.6 -0.0  2.10 -1.48  2.14  2
(  1  1  0        -1  1  0;   2, -2;   0, -1)   [      0      0     -1]
   4  0  0 51.179  1  1  0 80.576  80.9  716.4  353.6 -0.1  2.11 -1.48  2.17  2
( -1  1  0         1  1  0;  -2,  2;   0,  1)   [      0      0      1]
   4  0 -1 49.668  1  1 -1 82.087  80.3  511.1  354.1  0.5 -2.74 -1.34  2.92
                                                [      1      3      4]
  -4  0  1 49.665 -1  1  1 82.089  80.2  511.1  354.1  0.6 -2.75 -1.34  2.96
                                                [     -1      3     -4]
   4  0 -1 49.084  1 -1  1 82.670  83.2  498.5  349.9 -2.3 -4.63 -2.50  5.86
                                                [     -1     -5     -4]
   4  0 -1 49.089  1  1  1 82.665  83.2  498.6  350.0 -2.4 -4.62 -2.49  5.87
                                                [      1     -5      4]
*
```

pg

```
 consec. # of data set? 0 or <0: next set (    4 )

-DC-
 dir.lc.:  28.6756   4.4446  19.4660  90.00 105.47  89.98, V(C):  2391.1 trik
 rec.lc.: 0.036183 0.224993 0.053302  90.01  74.53  90.02, SG. C (***)

 4.39
   r1:  75.40 +- 2.26; ang.:   80.46 +-  2.50;   L0:    0.00; wgt(angle): 0.6
   r2:  81.63 +- 2.45; c.c.:  358.90 +- 17.59; L1-0:    0.00; wgt(r1/r2): 0.8
   r3: 101.53 +- 0.00; volt:     200000.     ; V(C):     0.0; wgt(c.c.) : 0.3
 mul.:    2, cent.:C, rl: 0, rewind: Y, nr:   199
*
```

i

```
      14 solution(s)
                                          L0:   0.0            200kV
   h1 k1 l1   r1  h2 k2 l2    r2  angle   L1-0   c.c.  ---- errors ----   R  mul
  obs.:     75.40            81.63  80.5    0.0  358.9 ang. r1/r2% c.c.%
 ------------------------------------------------------------------------------
  -6  0  1 75.173 -1  1  0 81.858  81.1  427.5  359.2 -0.7 -0.58  0.08  0.89
                                                [     -1     -1     -6]
   6  0 -1 75.178  1  1  0 81.853  81.2  427.5  359.2 -0.7 -0.57  0.09  0.90
                                                [      1     -1      6]
   6  0 -2 75.530  1  1 -1 81.501  79.1  410.7  351.6  1.3  0.33 -2.04  1.68
                                                [      1      2      3]
  -6  0  2 75.527 -1  1  1 81.504  79.1  410.7  351.6  1.4  0.32 -2.04  1.70
                                                [     -1      2     -3]
  -6  0  1 74.502 -1  1  1 82.529  81.6  419.8  356.0 -1.1 -2.29 -0.81  2.74
                                                [     -1      5     -6]
   6  0 -1 74.505  1  1 -1 82.526  81.6  419.8  356.0 -1.2 -2.28 -0.81  2.76
                                                [      1      5      6]
  -6  0  2 76.198 -1  1  0 80.833  82.0  416.2  354.7 -1.5  2.04 -1.17  2.89
                                                [     -1     -1     -3]
   6  0 -2 76.203  1  1  0 80.828  82.0  416.2  354.7 -1.5  2.05 -1.17  2.92
                                                [      1     -1      3]
   6  0 -1 73.766  1 -1  1 83.265  81.2  412.0  352.5 -0.7 -4.17 -1.79  4.29
                                                [     -1     -7     -6]
   6  0 -1 73.772  1  1  1 83.259  81.2  412.0  352.5 -0.7 -4.16 -1.79  4.30
                                                [      1     -7      6]
   4  0  2 73.659  1 -1  0 83.372  82.1  627.2  365.8 -1.7 -4.44  1.93  5.14  2
(  1  1  1         1 -1  0;   2,  2;   0,  1)   [      1      1     -2]
   4  0  2 73.664  1  1  0 83.367  82.2  627.2  365.9 -1.7 -4.43  1.94  5.15  2
(  1 -1  1         1  1  0;   2,  2;   0,  1)   [     -1      1      2]
  -2  0  4 73.166  1  1  1 83.865  78.4  420.1  355.1  2.0 -5.70 -1.07  6.11
                                                [     -2      3     -1]
  -2  0  4 73.160  1 -1  1 83.871  78.4  420.0  355.0  2.0 -5.72 -1.08  6.12
                                                [      2      3      1]
*
```

pg

```
 consec. # of data set? 0 or <0: next set (    5 )

-DC-
 dir.lc.:  28.6756   4.4446  19.4660  90.00 105.47  89.98, V(C):  2391.1 trik
 rec.lc.: 0.036183 0.224993 0.053302  90.01  74.53  90.02, SG. C (***)
```

```
 3.3
   r1:  27.63 +- 0.83; ang.:   89.10 +-  2.50;   L0:    0.00; wgt(angle): 0.6
   r2: 244.37 +- 7.33; c.c.:  358.90 +- 17.59; L1-0:    0.00; wgt(r1/r2): 0.8
   r3: 245.50 +- 0.00; volt:     200000.      ; V(C):     0.0; wgt(c.c.) : 0.3
 mul.:    2, cent.:C, rl: 0, rewind: Y, nr:   199
*
i
      12 solution(s)
                                             L0:   0.0            200kV
   h1  k1  l1    r1   h2  k2  l2     r2  angle   L1-0    c.c.  ---- errors ----   R   mul
  obs.:     27.63           244.37  89.1    0.0   358.9 ang. r1/r2% c.c.%
 -----------------------------------------------------------------------------
  -2   0   1 27.646  1 -3  2244.356  88.4  399.1  356.3  0.7  0.07 -0.73  0.71
                                                 [     3     5      6]
  -2   0   1 27.648  1  3  2244.354  88.3  399.2  356.3  0.8  0.08 -0.72  0.73
                                                 [    -3     5     -6]
   2   0  -1 27.650  9 -1  9244.352  87.2  399.3  356.3  1.9  0.09 -0.71  1.44
                                                 [    -1   -27     -2]
   2   0  -1 27.654  9  1  9244.348  87.2  399.5  356.4  1.9  0.10 -0.70  1.45
                                                 [     1   -27      2]
  -2   0   1 27.377  6  2  8244.625  88.0  556.1  352.8  1.1 -1.02 -1.69  1.99  2
( -2   0   1         3  1  4;   1,   0;    0,  2)    [    -1    11     -2]
  -2   0   1 27.371  6 -2  8244.631  88.0  555.9  352.7  1.1 -1.04 -1.71  2.00  2
( -2   0   1         3 -1  4;   1,   0;    0,  2)    [     1    11      2]
   2   0  -1 28.009  1  3  0243.993  87.7  407.4  361.0  1.4  1.52  0.58  2.22
                                                 [     3    -1      6]
  -2   0   1 28.008 -1  3  0243.994  87.7  407.3  361.0  1.4  1.52  0.57  2.24
                                                 [    -3    -1     -6]
   2   0  -1 27.272  3  3  2244.730  87.2  390.9  351.5  1.9 -1.45 -2.07  2.94
                                                 [     3    -7      6]
   2   0  -1 27.268  3 -3  2244.734  87.1  390.8  351.4  2.0 -1.46 -2.08  2.98
                                                 [    -3    -7     -6]
   2   0   0 27.013  5  1-12244.989  89.1  442.6  373.3 -0.0 -2.51  4.01  3.21
                                                 [     0    12      1]
  -2   0   0 27.013 -5  1 12244.989  89.1  442.6  373.3  0.0 -2.51  4.01  3.21
                                                 [     0    12     -1]
*
ax
  #        d1   s/d(%)    d2  s/d(%)   d3     ang. sig.     V      d-m   seq.
  1 V     13.8185 3.00   4.3885 3.00   4.3879  80.9  2.5     0.0  7.837  1
  2       12.9430 3.00   3.9086 3.00   3.8245  85.6  2.5     0.0  7.123  2
  3        7.1041 3.00   4.4181 3.00   4.0530  80.8  2.5     0.0  5.639  3
  4        4.7600 3.00   4.3966 3.00   3.5349  80.5  2.5     0.0  4.607  4
  5       12.9900 3.00   1.4687 3.00   1.4619  89.1  2.5     0.0  4.368  5
 exclude or (<0) include which number?
 abs. value > 20 : exclude or include all
1
  #        d1   s/d(%)    d2  s/d(%)   d3     ang. sig.     V      d-m   seq.
  1 V e   13.8185 3.00   4.3885 3.00   4.3879  80.9  2.5     0.0  0.000  5
  2       12.9430 3.00   3.9086 3.00   3.8245  85.6  2.5     0.0  7.123  1
  3        7.1041 3.00   4.4181 3.00   4.0530  80.8  2.5     0.0  5.639  2
  4        4.7600 3.00   4.3966 3.00   3.5349  80.5  2.5     0.0  4.607  3
  5       12.9900 3.00   1.4687 3.00   1.4619  89.1  2.5     0.0  4.368  4
*
pc
  #        d1   s/d(%)    d2  s/d(%)   d3     ang. sig.     V      d-m   seq.
  1 V e   13.8185 3.00   4.3885 3.00   4.3879  80.9  2.5     0.0  0.000  4
  2       12.9430 3.00   3.9086 3.00   3.8245  85.6  2.5     0.0  7.123  0
  3        7.1041 3.00   4.4181 3.00   4.0530  80.8  2.5     0.0  5.639  1
  4        4.7600 3.00   4.3966 3.00   3.5349  80.5  2.5     0.0  4.607  2
  5       12.9900 3.00   1.4687 3.00   1.4619  89.1  2.5     0.0  4.368  3

 a*,b*-defining:  2;
       sequence:       3;   4;   5;

   1st: new a*,b* defining number (0: no changes)
   2nd: <0: enforce full grid, >0: * sigma (1.79mm) = "wall thickness"
```

```
 V(P)(min), V(P)(max)? (calc.: min:     0. max:     0., mean:     0.)
 def.:     0.     0.
0 1500
    207.  1500.
 factor for default increment (0.025), def.:1., max:6; <0 : increment

    0.025

    274220 sets within  79 layers, p:  0.573 -  4.150
*
dc
  mult.<mu>: 2; <rl>: 0;  1st layer: V: 207.0; n:  259; p:  0.573
  ok?

 *** 52 solutions in C-memory, delete? ("n" will falsify int.!)


      100 solutions stored,  R :     0.27 -   0.64, incl. equiv.:     98941
        R      a     b     c      al     be     ga      x       y        V     int.
   1  0.27  4.48 14.46 14.48  114.1  94.0  98.7  0.420  0.131   836.5  0.00
   2  0.31  9.04 11.46 14.17   66.3  82.0  80.1 -0.113  0.414  1321.2  0.00
   3  0.32  5.27 13.80 21.60   73.2  85.5  88.4 -0.217  0.119  1500.0  0.00
   4  0.33  4.47 14.42 20.82  103.0  93.1  98.5  0.269  0.085  1288.0  0.00
   5  0.35  7.93  9.39 13.41  101.3  96.4 106.1  0.304  0.468   925.9  0.00
   6  0.38 10.03 10.34 13.60   75.6  75.9  73.7  0.256  0.377  1288.0  0.00
   7  0.41  4.47 14.52 23.63   97.1  93.5  98.8  0.239  0.073  1500.0  0.00
   8  0.41  9.36 12.14 14.17  108.2 100.1 104.3  0.407  0.409  1425.7  0.00
   9  0.42  4.47 14.13 14.39  113.4  98.4  94.9  0.420  0.134   815.5  0.00
  10  0.42  4.47 14.43 19.51  106.3  90.7  98.6  0.292  0.092  1193.6  0.00
  11  0.43  7.48 12.20 13.05   87.3  82.8  73.8  0.014  0.285  1134.5  0.00
  12  0.43  4.47 14.38 23.85   81.3  86.9  81.6  0.228  0.073  1500.0  0.00
  13  0.44  4.43 14.87 17.83  107.2  94.2  97.1  0.368  0.095  1106.0  0.00
  14  0.45  4.67 15.77 18.23   79.6  89.4  89.9  0.412  0.098  1321.2  0.00
  15  0.46  5.01 14.91 18.01   73.6  86.2  88.4  0.372  0.123  1288.0  0.00
  16  0.46  8.95 13.22 14.50  112.6  98.9 101.5  0.391  0.427  1500.0  0.00
  17  0.46  4.47 13.01 25.92   88.6  88.2  84.3 -0.011  0.073  1500.0  0.00
  18  0.46  8.01 10.70 13.10   87.9  81.0  76.4  0.000  0.329  1078.3  0.00
  19  0.46  4.52 13.91 22.22   75.8  89.5  88.2 -0.220  0.085  1355.1  0.00
  20  0.47  4.67 14.40 20.56   75.0  84.1  82.1  0.275  0.098  1321.2  0.00
  21  0.47  5.98 15.04 17.41  107.5  98.7  94.6  0.400  0.156  1462.4  0.00
  22  0.47 10.73 11.77 14.09   69.2  72.6  66.7  0.337  0.338  1500.0  0.00
  23  0.47  4.52 14.15 22.56   76.9  85.3  82.2  0.226  0.082  1390.0  0.00
  24  0.48  7.86 13.63 14.65   73.8  78.8  78.7 -0.256  0.490  1462.4  0.00
  25  0.48  9.05 12.31 14.20  113.4  92.9  98.5  0.477  0.290  1425.7  0.00
  26  0.49  4.52 15.47 21.59   86.0  89.3  84.7 -0.326  0.076  1500.0  0.00
  27  0.49  4.47 14.61 23.12   96.4  91.1  91.5 -0.261  0.073  1500.0  0.00
  28  0.49  7.16 13.33 15.52   78.1  82.5  81.4  0.163  0.213  1425.7  0.00
  29  0.49  4.48 14.52 17.95  109.8  93.1  98.7  0.333  0.102  1078.3  0.00
  30  0.49  8.95 10.37 13.01   88.4  84.4  83.4  0.139  0.408  1193.6  0.00
  31  0.50  8.08 12.35 12.99   91.0  94.9  94.5  0.141  0.462  1288.0  0.00
  32  0.50  4.57 14.39 19.29   72.2  88.2  81.2  0.292  0.100  1193.6  0.00
  33  0.52  4.57 13.32 22.89   76.7  89.9  87.1  0.134  0.088  1355.1  0.00
  34  0.52  7.88 11.48 13.88  107.9  96.2 104.3  0.343  0.482  1134.5  0.00
  35  0.52  4.73 15.93 19.58   95.8  94.7  91.2  0.389  0.092  1462.4  0.00
  36  0.53  5.90 13.58 14.88  103.8  93.8  99.8  0.229  0.197  1134.5  0.00
 cont.?
n
*
```

S6. Calibration of electron diffraction patterns – camera length, camera constant, units of measurement

In the field of materials-science transmission electron microscopy, images and diffraction patterns are traditionally calibrated using effective pixel sizes in the specimen plane (in Å or nm), or reciprocal space (in $Å^{-1}$ or nm^{-1}), respectively. It is furthermore customary to define a camera constant C:

$$C = L \cdot \lambda = R \cdot d$$

where L is the camera length (optical distance between specimen and camera), λ is the electron wavelength, R is the measured distance between the primary beam and the reflection, d is the interplanar distance of the reflection. The second identity is equivalent to Bragg's law in the small-angle limit of $sin\, 2\theta \approx tan\, 2\theta = R/L$, which is generally fulfilled for electron diffraction at realistic resolutions.
With the typically used units (mm for R and L; Å for d and λ), the camera constant C has units of [mm·Å], and the camera length L, in mm. Note that the camera length is an effective value, given by the electron-optical magnification of the projector system, and is not directly related to the distance between the sample and the detector.
In the era of digital detectors, when the pixel size is determined in Å^{-1}, it is sufficient to measure the distance in pixels and multiply it with the calibrated pixel size. In these conditions, the camera length L is measured in pixels, and the corresponding camera constant C in [pix·Å]. The camera constant C and the camera length can then be calculated as following:

$$C = \frac{1}{pixel\ size}$$

$$L = \frac{1}{\lambda \cdot pixel\ size}$$

Note, that the physical size of the pixel of the detector is not used anywhere.
In X-ray diffraction field the patterns are calibrated through the fixed distance between the crystal and the detector. In this situation, the knowledge of the physical size of the pixel on the detector becomes essential. For electron diffraction data, the effective distance to the detector D can be calculated using the same basic equation:

$$C = D \cdot \lambda = physical\ pix\ size \cdot \frac{1}{pix\ size} = \frac{physical\ pix\ size}{pix\ size}$$

S6.1. $CuPcCl_{16}$

For $CuPcCl_{16}$, the data were recorded on Gatan Ultrascan 1000XP CCD with the physical pixel size of 14µm, the nominal camera length of 600 mm, pixel size 0.00091 Å^{-1}. The data were recorded with 300kV electron beam, corresponding to the wavelength of 0.0197 Å.

The distance to detector D can be calculated as follows:

$$D = \frac{physical\ pix\ size}{\lambda \cdot pix\ size} = \frac{0.014}{0.0197 \cdot 0.00091} = 781mm$$

S6.2. Lysozyme data

The data were collected with 55 µm pixels detector, and the detector distance of 1580 mm. The electron wavelength for 200kV electron beam is 0.0251 Å. The associated pixel size can be calculated as follows:

$$pix\ size = \frac{physical\ pix\ size}{\lambda \cdot D} = \frac{0.055}{0.0251 \cdot 1580} = 0.00139\ \text{Å}^{-1}$$

Camera constant C is given by:

$$C = D \cdot \lambda = 1580 \cdot 0.0251 = 39.658\, mm \cdot \text{Å} = \frac{1}{pixel\ size} = 719.42\, pix \cdot \text{Å}$$

S6.3. GRGDS

For GRGDS data, recorded on CETA (Thermofischer) camera with the physical pixel size of 14µm, the nominal camera length was 840 mm, binning 4 was used, resulting in the pixel size of 0.002786 Å^{-1}. The data were collected at 200 kV. The distance to detector *D* and the camera constant *C* can be calculated as follows:

$$D = \frac{physical\ pix\ size}{\lambda \cdot pix\ size} = \frac{0.014 \cdot 4}{0.0251 \cdot 0.002786} = 800.8mm$$

$$C = D \cdot \lambda = 800.8 \cdot 0.0251 = 20.1\, mm \cdot \text{Å} = \frac{1}{pixel\ size} = 358.9\, pix \cdot \text{Å}$$

S7. Literature

Bücker, R., Hogan-Lamarre, P. & Miller, R. J. D. (2021). *Front. Mol. Biosci.* 8, 624264.

Jiang, L., Georgieva, D. & Abrahams, J. P. (2011). *J. Appl. Cryst*. 44, 1132–1136.

Kabsch, W. (1993). *J Appl Cryst* 26, 795–800.
